# Review of Recurrently Mutated Genes in Craniosynostosis Supports Expansion of Diagnostic Gene Panels

**DOI:** 10.3390/genes14030615

**Published:** 2023-02-28

**Authors:** Rebecca S. Tooze, Eduardo Calpena, Astrid Weber, Louise C. Wilson, Stephen R. F. Twigg, Andrew O. M. Wilkie

**Affiliations:** 1 Clinical Genetics Group, MRC Weatherall Institute of Molecular Medicine, University of Oxford, Oxford OX3 9DS, UK; 2 Liverpool Centre for Genomic Medicine, Liverpool Women’s NHS Foundation Trust, Liverpool L8 7SS, UK; 3 North East Thames Regional Genetics Service, Great Ormond Street Hospital for Children NHS Foundation Trust, London WC1N 3JH, UK

**Keywords:** craniosynostosis, PanelApp, exome/genome sequencing, gene-panels

## Abstract

Craniosynostosis, the premature fusion of the cranial sutures, affects ~1 in 2000 children. Although many patients with a genetically determined cause harbor a variant in one of just seven genes or have a chromosomal abnormality, over 60 genes are known to be recurrently mutated, thus comprising a long tail of rarer diagnoses. Genome sequencing for the diagnosis of rare diseases is increasingly used in clinical settings, but analysis of the data is labor intensive and involves a trade-off between achieving high sensitivity or high precision. PanelApp, a crowd-sourced disease-focused set of gene panels, was designed to enable prioritization of variants in known disease genes for a given pathology, allowing enhanced identification of true-positives. For heterogeneous disorders like craniosynostosis, these panels must be regularly updated to ensure that diagnoses are not being missed. We provide a systematic review of genetic literature on craniosynostosis over the last 5 years, including additional results from resequencing a 42-gene panel in 617 affected individuals. We identify 16 genes (representing a 25% uplift) that should be added to the list of bona fide craniosynostosis disease genes and discuss the insights that these new genes provide into pathophysiological mechanisms of craniosynostosis.

## 1. Introduction

Craniosynostosis, the premature fusion of one or more cranial sutures of the skull, is a clinically and genetically heterogeneous congenital anomaly, affecting approximately 1 in 2000 live births [1,2]. Despite being one of the most prevalent craniofacial abnormalities (second to cleft lip and/or cleft palate), the variability in its causes and presentation can make identifying a genetic diagnosis extremely challenging. Monogenic, polygenic [3,4], chromosomal, and environmental [5] factors have all been identified as likely causes of craniosynostosis, with a burden of over 20% of the cases originating from monogenic causes alone [6]. Although a majority of diagnoses are attributable to variants in just seven genes (*EFNB1, ERF, FGFR2, FGFR3, SMAD6, TCF12* and *TWIST1*) [6,7,8], over 60 genes are known to be recurrently mutated in craniosynostosis more rarely [9,10]. For example, in an Oxford survey of 666 individuals with craniosynostosis, pathogenic variants were identified in 20 more rarely mutated genes in 23/666 individuals (3.5%) [6]. The observation of a long tail of rarer disease-causing variants was supported by a recent exome sequencing study of patients in Norway [1]. The identification of such rare causative variants is inevitably challenging in a clinical diagnostic setting, where the need for high sensitivity (recall), which minimizes false negative calls but involves intensive effort, has to be balanced against the need for high precision (positive predictive value), which minimizes false positive calls [11]. The use of diagnostic gene panels has been adopted to address this problem.

A test case is provided by the UK’s 100,000 Genomes Project (100 kGP), delivered by Genomics England Limited (GE) between 2014 and 2019 [12]. This initiative aimed to facilitate whole genome sequencing (WGS) for 100,000 National Health Service (NHS) patients or relatives with rare diseases or cancer, with the primary aim being to return information to participants on variants with sufficient evidence for diagnostic reporting related to their primary condition [12]. To facilitate this, GE curated PanelApp, a publicly available resource containing crowdsourced and disease-focused gene panels for all rare disease groupings within the 100 kGP (https://panelapp.genomicsengland.co.uk/; accessed on 12 December 2022) [13]. Genes on each panel are traffic-light coded based on the level of confidence for diagnostic reporting in a given rare disease. Green genes are those in which there are plausible disease-causing variants (de novo or rare variants that are fully-penetrant) that affect a functional region of the gene (open reading frame for protein-coding genes) and have been identified in three or more unrelated families with a specific rare disease, or within two or more unrelated families with strong additional functional data. Full criteria for categorization of Green panel genes are provided in Table S1. Scrutiny of variants identified in Green genes aids prioritization of likely pathogenic variants and decreases the number candidates that diagnostic laboratories are required to screen. Genes that do not meet these criteria are listed as Amber or Red, corresponding to moderate or insufficient evidence for gene-disease association, respectively [13].

While the use of panels greatly facilities workflow through complex genomic datasets, their full utility is critically dependent on regular updating to take account of recent research findings, in order to maximize diagnostic sensitivity. A recent analysis of the diagnostic sensitivity achieved for craniosynostosis from the 100 kGP showed that only 47% of variants had been identified through the panel-based approach in use at that time. Although some of the missing diagnoses were attributable to a failure to call variants included in the contemporaneous Green panel (33% of missing diagnoses), an additional 22% of diagnoses were missed because the gene was considered an Amber or Red gene at the time of analysis, not taking into account more recent discoveries that indicated the gene should have been prioritized more highly [11].

Genomic diagnostics in England has now evolved from the 100 kGP initiative into the NHS England Genomic Medicine Service (www.england.nhs.uk/genomics/nhs-genomic-med-service/; accessed 23 January 2023). This service continues to rely on PanelApp to provide lists of genes to prioritize, however there appears to be no systematic mechanism to ensure that panels remain up to date. Here, we aimed to review the current genes listed as Amber or Red on PanelApp for craniosynostosis, to ascertain whether additional evidence was sufficient to promote a gene to Green status, thus flagging variants within that gene for clinical review by diagnostic laboratories. Additionally, we screened the literature for variants in genes not documented in PanelApp to provide an updated list of genes that should be monitored for further cases or that should already be considered a Green gene. We augmented this list with new data obtained from resequencing a 42-gene panel in 617 affected individuals.

## 2. Materials and Methods

### 2.1. Literature Search

Articles were searched on PubMed using “craniosynostosis” as a keyword across a 5-year period from 2018 to end of 2022 (Table 1 and Table S2), as reports prior to this date should have already been incorporated into PanelApp. All exome, genome, or panel-based analyses of patients with craniosynostosis were included and screened for variants in genes listed as Amber or Red on PanelApp (v3, accessed 12 December 2022) [13] (Table S3). Any additional gene not listed on PanelApp with a variant annotated as likely pathogenic/pathogenic was included in the analysis (Table S4). For all genes listed as Amber or Red on PanelApp, a search on PubMed was conducted using the gene name as a keyword across all time periods to identify further case reports to support gene-disease association (Table S3); some of these papers had already been considered in PanelApp. Additionally, we searched for any further published single case reports of craniosynostosis associated with a novel gene to add to the list of genes not described in PanelApp (Table S4).

### 2.2. Panel-Based Sequencing of a Cohort of Genetically Unsolved Patients with Craniosynostosis

In total, 617 samples (considering a variety of sutures fused) were screened for pathogenic variants in 42 genes (Table S2) using IDT’s hybridization and capture protocol (further details in Supplementary Materials). Probes were designed to ensure that all coding regions of the canonical transcript were captured by at least two probes (probes used to target *SOX6* and *SMAD3* are detailed in Table S5). Sequencing data were analyzed using amplimap software [27] (including mapping, coverage analysis, and variant calling), and variants were filtered on the basis of rarity (allele frequency in gnomAD [v2.1.1] below 0.000045) [8,28], CADD score (≥20, or not reported), and likely consequence (missense or more damaging).

### 2.3. Analysis of Single Cell Transcriptomic Data

For any gene in which there was new, convincing, evidence for variant pathogenicity identified in three or more individuals (from the literature or resequencing analysis), or two or more cases with additional functional evidence, the expression of the gene was analyzed from previously published single cell transcriptomic data of the mouse embryonic day (E) 15.5–17.5 coronal suture [29]. Complete methods and bioinformatic analyses are described in detail by Farmer et al., 2021.

## 3. Results

Sequencing of 42 genes in 617 unsolved samples identified ten variants considered likely pathogenic. Of these, four were identified in Green genes (three *ALX4* variants and one *MSX2*)*,* and the remaining six variants were identified in Amber genes. The Amber genes included three variants in *PRRX1* (which contributed towards the first experimental cohort of patients with craniosynostosis and variants in *PRRX1* [30], providing substantial evidence to promote this gene to Green), one splicing variant in *SMAD3* (c.206+1G>A; p.(?)), and two truncating variants in *SOX6* (Table S3, Figure S1). The two *SOX6* variants comprised a 23 bp deletion encompassing the exon 3-intron 3 boundary (c.426_445+13del; p.(?)) and a de novo stop-gain (c.1624G>T; p.(Glu542*)) (Figure S1). For the individual with the *SOX6* deletion variant, parental samples were not available for screening. While the identification of additional patients with variants in *SMAD3* would be required to promote its current Amber PanelApp status, the two variants in *SOX6* provide a significant uplift (40%) to the total number of patients currently described in the literature with craniosynostosis and contribute positively to promoting this gene to Green (Table 2).

Following a framework for evidence-based gene-disease-association classification (Table S1) [57,58], a review of the current literature on variants reported in patients with craniosynostosis alongside the results from our panel-based resequencing analysis (Table 1) suggests that an additional 16 genes should be promoted to Green status (Table 2), bringing the total number of Green genes to 81 (a 25% increase). Four of these are currently classified as Amber (*MASP1, NFIA, PRRX1,* and *SOX6*), six genes are annotated as Red (*ADAMTSL4, AHDC1, FBN1, FGF9, KAT6B,* and *NFIX*), and six genes are not included in PanelApp (*ARID1B, BCL11B, CDK13, FBXO11, IL6ST,* and *MAN2B1*). Further details on these 16 proposed new Green genes are provided in the Supplementary Materials section.

Single cell transcriptomic data from the mouse E15.5–17.5 coronal suture was analyzed to provide additional information for interpreting the pathophysiological mechanisms associated with the 16 genes identified. Nine of these genes displayed generalized expression across all cell populations, but only *Nfia*, *Nfix* and *Prrx1* were shown to be highly expressed in all clusters (Figure S2). The remaining seven genes showed specific expression patterns (Figure S3), with *Bcl11b* and *Sox6* expressed in osteogenic cells, while *Il6st* was predominately expressed in non-osteogenic cell clusters. *Masp1* was identified in the suture periosteum and *Fgf9* showed low but specific expression within cells of the suprasutural layer. *Fbn1* and *Adamtsl4* showed specific expression within cells of the ectocranial layers, although *Fbn1* was more widely expressed in the ectocranial clusters. Details on the role of each cell population in the development of the coronal suture were discussed previously [29].

## 4. Discussion

Based on the identification of three or more individuals harboring variants associated with congruent phenotypes and likely damaging variants in a common gene, to date, there are 65 genes classified as Green in PanelApp. Of those, 24 (37%) may be considered “core” craniosynostosis-associated genes, in which variants of a particular molecular type are associated with craniosynostosis in over 50% of the individuals and are considered to perturb fundamental components in the biology of cranial suturogenesis. The remaining genes are those in which craniosynostosis is a phenotype less frequently associated with pathogenic variants within that gene, although likely causally associated. This latter grouping includes some examples in which there is a wider disturbance in osteogenesis, owing to aberrant osteoblast or osteoclast activity.

Of the 16 genes presented here as newly associated craniosynostosis genes, *PRRX1* and *FGF9* may be considered as core genes. *PRRX1* encodes the mammalian paired-related homeobox 1 transcription factor, a member of the PRD class of homeobox transcription factors that regulate several aspects of embryonic development [59]. Post-natal calvarial stem cells expressing *Prrx1* have been shown to reside exclusively in the calvarial suture niche [60], suggesting a requirement for PRRX1 regarding suture patency during early development. In support, *Prrx1* has been shown to be widely expressed within the mouse coronal suture at E15.5 [29] (Figure S2). Postnatal skeletal stem cells expressing *Prrx1* were also shown to respond to WNT signaling by differentiating into osteoblasts and can regenerate bone upon heterotopic transplantation [60], supporting a key role for PRRX1 in the maintenance of the sutural mesenchyme and flanking bone fronts.

*FGF9* encodes fibroblast growth factor 9, an essential growth factor for intramembranous ossification. Positive differentiation signals emanating from osteoid (a collagenous unmineralized matrix produced by osteoblasts), alongside provision of growth factors (including *FGF9*) from the dura mater underlying the suture [61,62], stimulate osteogenesis of the surrounding bone fronts. In support, analysis of single cell data shows *Fgf9* predominantly expressed within the suprasutural layer, but also within small populations of cells occupying the outer dura mater and osteoprogenitors (Figure S3) [29]. This spatial distribution permits signaling interactions between mesenchymal populations and osteogenic cells, thus controlling differentiation and proliferation of osteoblasts to establish the growing bone fronts flanking the suture [63,64,65]. Notably, a mouse semidominant mutant (Elbow knee synostosis; *Eks*) is caused by the amino acid substitution p.(Asn143Thr) in *Fgf9* and manifests elbow joint fusions, knee joint dysplasia, and craniosynostosis [46,66,67]; importantly, variants in multiple members of the FGF receptor family cause several classical craniosynostosis syndromes, including Apert, Crouzon, Pfeiffer and Muenke syndromes [9].

The remaining 14 craniosynostosis-associated genes can be broadly divided into five categories of pathophysiology: defects in the extracellular matrix (ECM) (*ADAMTSL4* and *FBN1*); regulators of cell cycle-progression and/or genome stability (*CDK13* and *FBXO11*); chromatinopathies (*ARID1B* and *KAT6B*); bone osteogenesis, resorption, and homeostasis (*IL6ST, MAN2B1,* and *MASP1*); and abnormalities in regulators of cell fate and differentiation (*AHDC1*, *BCL11B, NFIA*, *NFIX*, and *SOX6*). Each of these categories, which are not mutually exclusive, is discussed briefly in turn (further details on each gene are described in the Supplementary Materials).

The ECM plays an important role in cell signaling by eliciting cues for cell proliferation, migration, and differentiation. For example, fibrillin-1 (*FBN1*) (an ECM glycoprotein) mediates the activation of transforming growth factor β (TGF-β) [9,40], which is known to be upregulated in osteoblasts [62]. This interaction is stabilized by ECM-binding proteins (including *ADAMTSL4*) at the cell matrix interface [68,69,70]; expression of *Adamtsl4* in the mouse coronal suture was identified within the ectocranial layers, overlapping with sites of expression of *Fbn1* (Figure S3).

Previously, hypomorphic variants in genes involved in the regulation of cell division, including *CDC45* and *RECQL4* (both Green genes) were reported in patients with craniosynostosis [9]. Members of the cyclin-dependent kinases (CDKs) regulate cell-cycle progression and gene expression through controlling cell-cycle checkpoints, and phosphorylation status and activity of splicing regulators [52]. Dysregulation of a number of CDKs promotes cell proliferation and is a hallmark of several cancers [71]. In addition, DNA damage response pathways respond to genotoxic stress induced by the presence of deleterious changes in the DNA sequence, by inducing cell cycle arrest and repair mechanisms. *FBXO11* belongs to the F-Box family of proteins which mediate protein-protein interaction for ubiquitin-mediated proteolysis [72]; *FBXO11* constitutes one subunit of an E3-ubiqitin ligase complex, and functions to recognize substrates for degradation [73], thus controlling genome stability. Both *Cdk13* and *Fbxo11* are expressed across all populations of the coronal suture (Figure S2). The importance of maintaining the balance between cell proliferation and differentiation within the suture is discussed later.

In line with recent reports, we identified chromatin modifiers as a category of pleiotropic genes associated with craniosynostosis [22,74], whereby the underlying genetic mechanism is the disruption of a component of epigenetic machinery (writers, erasers, and readers). In keeping with the heterogenous function of chromatin modifiers, the expression of *Arid1b* and *Kat6b* is generally widespread across tissues of the mouse coronal suture (Figure S2). Targets of epigenetic modification include the DNA itself through alterations to methylation status, or by modification of the DNA-associated histone proteins [75]. The effect of variation within these genes is expected to be widespread, although a defining feature of patients with chromatinopathies is usually the presence of intellectual disabilities [75].

Genes involved in immunity have previously been highlighted as important molecules in the maintenance of the balance between bone growth, mediated by osteoblasts, and resorption, mediated by osteoclasts [9]. *IL6ST* encodes GP130, a cytokine receptor and transducer of IL6-mediated cytokine signaling. Perturbations in cytokine signaling affect bone remodeling due to a defect in osteoclast differentiation; for example, biallelic loss-of-function variants in the interleukin 11 (*IL-11*) co-receptor, *IL11RA*, cause craniosynostosis, possibly owing to an osteoclast defect and subsequent failure to break down the bone matrix. In support, *Il6st* was predominantly expressed in the non-osteogenic populations above and below the suture (Figure S3). *MASP1*, which encodes the mannan-binding lectin serine protease-1, functions in the lectin pathway of complement. Osteoclast-derived complement factors were shown to stimulate osteoblast differentiation, shifting the balance towards increased bone growth potential [76,77]. Consistent with this function, *Masp1* exhibits specific expression within a pre-osteoblast population of the coronal suture (Figure S3). *MAN2B1* encodes α-D-mannosidase, which functions in the lysosomal maturation of N-linked glycoproteins. Biallelic defects in *MAN2B1* lead to multisystem accumulation of undigested oligosaccharides in the lysosomes, affecting the function of many cell types including osteogenic cells.

For the maintenance of the cranial suture, a population of undifferentiated stem cells must persist within the mid-sutural mesenchyme [9]. A disruption in the delicate balance of proliferation and differentiation, associated with increased cell proliferation and/or accelerated osteogenic differentiation, may result in craniosynostosis. The genes *AHDC1*, *BCL11B*, *NFIA*, *NFIX* and *SOX6* all encode transcription factors, the haploinsufficiency of which may perturb cell regulation in specific contexts. *SOX6* has previously been shown to function as a tumor suppressor [78,79], limiting cell proliferation; in support, we identified expression of *Sox6* within the sutural mesenchyme and progenitor cell populations surrounding the suture (Figure S3). The expression of *Bcl11b* is specifically enriched within the osteoprogenitor cell populations (Figure S3); this finding is consistent with studies in mice that have highlighted an essential role of *Bcl11b* in suture biogenesis. Complete loss of *Bcl11b* in mice was associated with increased proliferation of osteoprogenitors and premature osteoblast differentiation, leading to synostoses of facial and calvarial sutures [80]. Additionally, mutation of a critical residue involved in binding the RBBP4-MTA1 complex, p.(Arg3Ser), was shown to cause coronal craniosynostosis at post-natal day 0 [49]. Cells expressing *Ahdc1*, *Nfia*, and *Nfix* showed more generalized expression across populations of the coronal suture (Figure S2). Accordingly, these genes are known to regulate multiple embryonic lineages and pathogenic variants were shown to cause varying phenotypes, with neurodevelopmental disorder a common feature [33,39,81].

## 5. Conclusions

A literature review of single case reports, and exome-, genome-, or panel-based analyses of patients with craniosynostosis, alongside the resequencing of 617 individuals with craniosynostosis for variants in 42 genes, identified 16 genes that should be newly promoted to Green on PanelApp according to gene-disease association criteria. Inclusion of these genes will facilitate the identification of additional variants in patients recruited to the 100 kGP. We highlight two core genes (*FGF9*, *PRRX1*) in which variants may impact key aspects of cranial suture biology, but for the majority of the genes it is likely that craniosynostosis is a rare consequence of mutation. While efforts have been made to update the craniosynostosis panel over recent years, this report provides (to our knowledge) the first systematic update of genes newly implicated in craniosynostosis since 2019. Additionally, the heterogenous nature of craniosynostosis is underlined, giving significant uplift to the number of genes identified in the long tail of rarer genetic diagnoses. In genes for which only one or two patients have currently been identified with a likely pathogenic variant, we hope this work will provide a resource for the identification and analysis of additional cases in the future.

## Figures and Tables

**Table 1:** Sequencing of cohorts of patients with craniosynostosis from 2018–2022.

Cohort	Number of Probands Screened	Sequencing Technology	Phenotypes Included in the Screen ^a^	Number of Pathogenic/Likely Pathogenic Variants Identified in Each Screen Corresponding to Current PanelApp (v3) Status			
				Green	Amber	Red	Null
Australia/New Zealand (Lee et al., 2018) [14]	309	20-gene panel	Patients recruited with a range of sutures fused, with or without syndromic features	40	2	1	
Seattle (Clarke et al., 2018) [15]	397	RNA-sequencing, 61 genes screened	Single suture craniosynostosis	43	1	19	
Scandinavia (Topa et al., 2019) [16]	100	63-gene panel	Syndromic craniosynostosis (78% of the cohort), predominately coronal synostosis	66			
Yale (Timberlake et al., 2019) [17]	12	Whole exome	All syndromic, with single and multi-suture synostosis	5			4
Japan (Suzuki et al., 2020) [18] ^b^	51	Whole exome	All with trigonocephaly		4	17	
Korea (Yoon et al., 2020) [19]	110	34-gene panel	Patients recruited with syndromic or non-syndromic craniosynostosis and all sutures considered	24		1	
China (Wu et al., 2021) [20]	201	17-gene panel	Cohort consists of patients with syndromic and non-syndromic craniosynostosis	51			
Saudi Arabia (Alghamdi et al., 2021) [21]	28	Whole exome	Syndromic craniosynostosis with all sutures considered	13		2	
100 kGP (Hyder et al., 2021) [11]	114	Whole genome	Patients recruited with syndromic or non-syndromic craniosynostosis and all sutures considered	12	3	3	16
Norway (Tønne et al., 2021) [22]	381	72-gene panel	Patients recruited with syndromic or non-syndromic craniosynostosis and all sutures considered	59		4	5
China (Chen et al., 2022) [23]	264	17-gene panel (264 individuals), whole-exome sequencing (n = 102, 39%)	Patients recruited with syndromic or non-syndromic craniosynostosis and all sutures considered	143	2		4
Norway (Tønne et al., 2022) [24]	10	Whole exome	All patients with syndromic craniosynostosis that were negative in the previous Tønne screen [22]		1		4
Yale (Timberlake et al., 2023) [25]	25	Whole exome	All patients displayed lambdoid synostosis			1	14
Oxford (Tooze et al., 2022) [26]	617	42-gene panel (Table S2)	Patients recruited with syndromic or non-syndromic craniosynostosis and all sutures considered	4	6		

^a^ See Table S2 for further information. ^b^ Only variants confirmed by dideoxy-sequencing within this study were included in variant counts.

**Table 2:** Genes with sufficient evidence to be updated to Green PanelApp status.

Gene.	Current Panel	Mode of Inheritance	Broad Categories of Pathophysiology	Literature
*MASP1*	Amber	Biallelic	Bone osteogenesis, resorption, and homeostasis	Two reviews identify a prevalence of 27–31% of patients with craniosynostosis and 3MC syndrome and a variant in *MASP1* [31,32].
*NFIA*	Amber	Monoallelic	Regulator of cell fate and differentiation	Four patients identified in independent screens [19,22,23,24].
*PRRX1*	Amber	Monoallelic	Regulator of cell fate and differentiation	There are 17 patients from 14 independent families with rare heterozygous variants in *PRRX1*, predicting loss of function variants or missense variants affecting the homeodomain [30].
*SOX6*	Amber	Monoallelic	Regulator of cell fate and differentiation	Seven independent families with loss of function variants in *SOX6* and craniosynostosis; five of these are published [23,33,34] and two were identified in a screen of 617 patients without a genetic diagnosis to their craniosynostosis [this study; Table S3].
*ADAMTSL4*	Red	Biallelic	Regulator of the extracellular matrix	More than 12 cases of ectopia lentis and craniosynostosis are associated with recessive variants in *ADAMTSL4* [35].
*AHDC1*	Red	Monoallelic	Regulator of cell fate and differentiation	There are three individuals reported with bona fide craniosynostosis and variants in *AHDC1* [36,37,38], a further four individuals are described with variants in *AHDC1* and suspected craniosynostosis [22,39].
*FBN1*	Red	Monoallelic	Regulator of the extracellular matrix	There are five likely pathogenic de novo variants reported in independent families and one deletion which includes *FBN1* [19,37,40,41,42].
*FGF9*	Red	Monoallelic	Regulator of cell fate and differentiation	Three likely pathogenic variants have been reported in independent families; one variant segregates in 12 individuals in the same family and another variant was inherited from an affected father [43,44,45]. A missense substitution, p.(Asn143Thr), in murine *Fgf9* results in a phenotype similar to multiple synostoses syndrome 3, with craniosynostosis [46].
*KAT6B*	Red	Monoallelic	Chromatinopathy	Three loss of function variants have been identified in patients with craniosynostosis and a phenotype similar to Lin-Gettig syndrome [22,47].
*NFIX*	Red	Monoallelic	Regulator of cell fate and differentiation	Four families are reported with variants in *NFIX*. Only one of these variants does not affect a functional domain (p.(Met48Lys)), but it is reported in ClinVar as likely pathogenic for Malan overgrowth syndrome [22,25].
*ARID1B*	Absent	Monoallelic	Chromatinopathy	Four independent families have been described with loss-of-function variants in *ARID1B* and craniosynostosis with developmental delay [11,18,23,48].
*BCL11B*	Absent	Monoallelic	Regulator of cell fate and differentiation	There are seven families with variants in *BCL11B* and confirmed craniosynostosis [49,50,51].
*CDK13*	Absent	Monoallelic	Cell-cycle regulator/genome stability	Four independent cases identified within the literature in patients with craniosynostosis [11,22,52].
*FBXO11*	Absent	Monoallelic	Cell-cycle regulator/genome stability	Three independent cases confirmed in patients [11,53].
*IL6ST*	Absent	Biallelic	Bone osteogenesis, resorption, and homeostasis	Two cases of recessive variants in *IL6ST* and craniosynostosis with additional animal models which phenocopy the human presentation [54,55].
*MAN2B1*	Absent	Biallelic	Bone osteogenesis, resorption, and homeostasis	Three independent families with recessive variants in *MAN2B1* and craniosynostosis, although not all individuals with recessive variants in *MAN2B1* develop craniosynostosis [11,22,56].

## Data Availability

All discussed data are presented in the article or Supplementary Materials.

## Supplementary Materials

The following supporting information can be downloaded at: https://www.mdpi.com/article/10.3390/genes14030615/s1, Figure S1: Pedigree figures and dideoxy-sequencing confirmation of *SOX6* (NM_033326.3) and *SMAD3* (NM_005902.4) variants; Figure S2: Genes displaying widespread expression across cell clusters; Figure S3: Coronal suture single cell transcriptomic map and specific expression patterns of seven genes; Table S1: Criteria for a diagnostic-grade Green gene on PanelApp; Table S2: Whole exome-, genome-, or panel-based analyses of patients with craniosynostosis between 2018–2022; Table S3: PanelApp Amber and Red genes and updated evidence for their association with craniosynostosis.; Table S4: Genes not currently listed in PanelApp but with one or more variants described in a patient with craniosynostosis; Table S5: Coordinates of the probes used in the target capture analysis of *SOX6* and *SMAD3*.

## References

[1] Tønne E., Due-Tønnessen B.J., Wiig U., Stadheim B.F., Meling T.R., Helseth E., Heimdal K.R. (2020). Epidemiology of craniosynostosis in Norway. J. Neurosurg. Pediatr..

[2] Cornelissen M., Ottelander B., Rizopoulos D., van der Hulst R., Mink van der Molen A., van der Horst C., Delye H., van Veelen M.L., Bonsel G., Mathijssen I. (2016). Increase of prevalence of craniosynostosis. J. Craniomaxillofac. Surg..

[3] Justice C.M., Yagnik G., Kim Y., Peter I., Jabs E.W., Erazo M., Ye X., Ainehsazan E., Shi L., Cunningham M.L. (2012). A genome-wide association study identifies susceptibility loci for nonsyndromic sagittal craniosynostosis near BMP2 and within BBS9. Nat. Genet..

[4] Justice C.M., Cuellar A., Bala K., Sabourin J.A., Cunningham M.L., Crawford K., Phipps J.M., Zhou Y., Cilliers D., Byren J.C. (2020). A genome-wide association study implicates the BMP7 locus as a risk factor for nonsyndromic metopic craniosynostosis. Hum. Genet..

[5] Sanchez-Lara P.A., Carmichael S.L., Graham J.M., Jr Lammer E.J., Shaw G.M., Ma C., Rasmussen S.A. (2010). National Birth Defects Prevention Study: Fetal constraint as a potential risk factor for craniosynostosis. Am. J. Med. Genet. A.

[6] Wilkie A.O.M., Johnson D., Wall S.A. (2017). Clinical genetics of craniosynostosis. Curr. Opin. Pediatr..

[7] Timberlake A.T., Furey C.G., Choi J., Nelson-Williams C., Loring E., Galm A., Kahle K.T., Steinbacher D.M., Larysz D., Yale Center for Genome Analysis (2017). De novo mutations in inhibitors of Wnt, BMP, and Ras/ERK signaling pathways in non-syndromic midline craniosynostosis. Proc. Natl. Acad. Sci. USA.

[8] Calpena E., Cuellar A., Bala K., Swagemakers S.M.A., Koelling N., McGowan S.J., Phipps J.M., Balasubramanian M., Cunningham M.L., Douzgou S. (2020). SMAD6 variants in craniosynostosis: Genotype and phenotype evaluation. Genet. Med..

[9] Twigg S.R.F., Wilkie A.O.M. (2015). A Genetic-Pathophysiological Framework for Craniosynostosis. Am. J. Hum. Genet..

[10] Goos J.A.C., Mathijssen I.M.J. (2019). Genetic Causes of Craniosynostosis: An Update. Mol. Syndromol..

[11] Hyder Z., Calpena E., Pei Y., Tooze R.S., Brittain H., Twigg S.R.F., Cilliers D., Morton J.E.V., McCann E., Weber A. (2021). Evaluating the performance of a clinical genome sequencing program for diagnosis of rare genetic disease, seen through the lens of craniosynostosis. Genet. Med..

[12] Smedley D., Smith K.R., Martin A., Thomas E.A., McDonagh E.M., Cipriani V., Ellingford J.M., Arno G., Tucci A., Vandrovcova J. (2021). 100,000 Genomes Pilot on Rare-Disease Diagnosis in Health Care-Preliminary Report. N. Eng. J. Med..

[13] Martin A.R., Williams E., Foulger R.E., Leigh S., Daugherty L.C., Niblock O., Leong I.U.S., Smith K.R., Gerasimenko O., Haraldsdottir E. (2019). PanelApp crowdsources expert knowledge to establish consensus diagnostic gene panels. Nat. Genet..

[14] Lee E., Le T., Zhu Y., Elakis G., Turner A., Lo W., Venselaar H., Verrenkamp C.A., Snow N., Mowat D. (2018). A craniosynostosis massively parallel sequencing panel study in 309 Australian and New Zealand patients: Findings and recommendations. Genet. Med..

[15] Clarke C.M., Fok V.T., Gustafson J.A., Smyth M.D., Timms A.E., Frazar C.D., Smith J.D., Birgfeld C.B., Lee A., Ellenbogen R.G. (2018). Single suture craniosynostosis: Identification of rare variants in genes associated with syndromic forms. Am. J. Med. Genet. A.

[16] Topa A., Rohlin A., Andersson M.K., Fehr A., Lovmar L., Stenman G., Kolby L. (2020). NGS targeted screening of 100 Scandinavian patients with coronal synostosis. Am. J. Med. Genet. A.

[17] Timberlake A.T., Jin S.C., Nelson-Williams C., Wu R., Furey C.G., Islam B., Haider S., Loring E., Galm A., Yale Center for Genome Analysis (2019). Mutations in TFAP2B and previously unimplicated genes of the BMP, Wnt, and Hedgehog pathways in syndromic craniosynostosis. Proc. Natl. Acad. Sci. USA.

[18] Suzuki T., Suzuki T., Raveau M., Miyake N., Sudo G., Tsurusaki Y., Watanabe T., Sugaya Y., Tatsukawa T., Mazaki E. (2020). A recurrent PJA1 variant in trigonocephaly and neurodevelopmental disorders. Ann. Clin. Transl. Neurol..

[19] Yoon J.G., Hahn H.M., Choi S., Kim S.J., Aum S., Yu J.W., Park E.K., Shim K.W., Lee M.G., Kim Y.O. (2020). Molecular Diagnosis of Craniosynostosis Using Targeted Next-Generation Sequencing. Neurosurgery.

[20] Wu Y., Peng M., Chen J., Suo J., Zou S., Xu Y., Wilkie A.O.M., Zou W., Mu X., Wang S. (2021). A custom-designed panel sequencing study in 201 Chinese patients with craniosynostosis revealed novel variants and distinct mutation spectra. J. Genet. Genom..

[21] Alghamdi M., Alhumsi T.R., Altweijri I., Alkhamis W.H., Barasain O., Cardona-Londono K.J., Ramakrishnan R., Guzman-Vega F.J., Arold S.T., Ali G. (2021). Clinical and Genetic Characterization of Craniosynostosis in Saudi Arabia. Front. Pediatr..

[22] Tønne E., Due-Tønnessen B.J., Mero I.L., Wiig U.S., Kulseth M.A., Vigeland M.D., Sheng Y., von der Lippe C., Tveten K., Meling T.R. (2021). Benefits of clinical criteria and high-throughput sequencing for diagnosing children with syndromic craniosynostosis. Eur. J. Hum. Genet..

[23] Chen J., Zhang P., Peng M., Liu B., Wang X., Du S., Lu Y., Mu X., Lu Y., Wang S. (2022). An additional whole-exome sequencing study in 102 panel-undiagnosed patients: A retrospective study in a Chinese craniosynostosis cohort. Front. Genet..

[24] Tønne E., Due-Tønnessen B.J., Vigeland M.D., Amundsen S.S., Ribarska T., Asten P.M., Sheng Y., Helseth E., Gilfillan G.D., Mero I.L. (2022). Whole-exome sequencing in syndromic craniosynostosis increases diagnostic yield and identifies candidate genes in osteogenic signaling pathways. Am. J. Med. Genet. A.

[25] Timberlake A.T., Kiziltug E., Jin S.C., Nelson-Williams C., Loring E., Allocco A., Marlier A., Banka S., Stuart H., Yale Center for Genome Analysis (2023). De novo mutations in the BMP signaling pathway in lambdoid craniosynostosis. Hum. Genet..

[26] Tooze R.S., Calpena E., Twigg S.R.F., D’Arco F. (2022). Genomics England Research C, Wakeling EL, Wilkie AOM: Craniosynostosis, inner ear, and renal anomalies in a child with complete loss of SPRY1 (sprouty homolog 1) function. J. Med. Genet..

[27] Koelling N., Bernkopf M., Calpena E., Maher G.J., Miller K.A., Ralph H.K., Goriely A., Wilkie A.O.M. (2019). Amplimap: A versatile tool to process and analyze targeted NGS data. Bioinformatics.

[28] Whiffin N., Minikel E., Walsh R., O’Donnell-Luria A.H., Karczewski K., Ing A.Y., Barton P.J.R., Funke B., Cook S.A., MacArthur D. (2017). Using high-resolution variant frequencies to empower clinical genome interpretation. Genet. Med..

[29] Farmer D.T., Mlcochova H., Zhou Y., Koelling N., Wang G., Ashley N., Bugacov H., Chen H.J., Parvez R., Tseng K.C. (2021). The developing mouse coronal suture at single-cell resolution. Nat. Commun..

[30] Tooze R.S., Miller K., Swagemakers S., Mcgowan S., Boute O., Collet C., Johnson D., Laffargue F., Leeuw N.D., Morton J. Pathogenic variants in the paired-related homeobox 1 (PRRX1) gene are associated with craniosynostosis [Conference Abstract]. Proceedings of the European Society of Human Genetics Conference.

[31] Durmaz C.D., Altiner S. (2021). MASP1-related 3MC syndrome in a patient from Turkey. Am. J. Med. Genet. A.

[32] Atik T., Koparir A., Bademci G., Foster J., Altunoglu U., Mutlu G.Y., Bowdin S., Elcioglu N., Tayfun G.A., Atik S.S. (2015). Novel MASP1 mutations are associated with an expanded phenotype in 3MC1 syndrome. Orphanet J. Rare Dis..

[33] Tolchin D., Yeager J.P., Prasad P., Dorrani N., Russi A.S., Martinez-Agosto J.A., Haseeb A., Angelozzi M., Santen G.W.E., Ruivenkamp C. (2020). De Novo SOX6 Variants Cause a Neurodevelopmental Syndrome Associated with ADHD, Craniosynostosis, and Osteochondromas. Am. J. Hum. Genet..

[34] Tagariello A., Heller R., Greven A., Kalscheuer V.M., Molter T., Rauch A., Kress W., Winterpacht A. (2006). Balanced translocation in a patient with craniosynostosis disrupts the SOX6 gene and an evolutionarily conserved non-transcribed region. J. Med. Genet..

[35] Gustafson J., Bjork M., van Ravenswaaij-Arts C.M.A., Cunningham M.L. (2022). Mechanism of Disease: Recessive ADAMTSL4 Mutations and Craniosynostosis with Ectopia Lentis. Case Rep. Genet..

[36] Gumus E. (2020). Extending the phenotype of Xia-Gibbs syndrome in a two-year-old patient with craniosynostosis with a novel de novo AHDC1 missense mutation. Eur. J. Med. Genet..

[37] Miller K.A., Twigg S.R., McGowan S.J., Phipps J.M., Fenwick A.L., Johnson D., Wall S.A., Noons P., Rees K.E., Tidey E.A. (2017). Diagnostic value of exome and whole genome sequencing in craniosynostosis. J. Med. Genet..

[38] Ritter A.L., McDougall C., Skraban C., Medne L., Bedoukian E.C., Asher S.B., Balciuniene J., Campbell C.D., Baker S.W., Denenberg E.H. (2018). Variable Clinical Manifestations of Xia-Gibbs syndrome: Findings of Consecutively Identified Cases at a Single Children’s Hospital. Am. J. Med. Genet. A.

[39] Danda S., Datar C., Kher A., Deshpande T., Thomas M.M., Oommen S.P. (2022). First reported cases with Xia-Gibbs syndrome from India harboring novel variants in AHDC1. Am. J. Med. Genet. A.

[40] Adès L.C., Sullivan K., Biggin A., Haan E.A., Brett M., Holman K.J., Dixon J., Robertson S., Holmes A.D., Rogers J. (2006). FBN1, TGFBR1, and the Marfan-craniosynostosis/mental retardation disorders revisited. Am. J. Med. Genet. A.

[41] Takenouchi T., Hida M., Sakamoto Y., Torii C., Kosaki R., Takahashi T., Kosaki K. (2013). Severe congenital lipodystrophy and a progeroid appearance: Mutation in the penultimate exon of FBN1 causing a recognizable phenotype. Am. J. Med. Genet. A.

[42] Hiraki Y., Moriuchi M., Okamoto N., Ishikawa N., Sugimoto Y., Eguchi K., Sakai H., Saitsu H., Mizuguchi T., Harada N. (2008). Craniosynostosis in a patient with a de novo 15q15-q22 deletion. Am. J. Med. Genet. A.

[43] Sentchordi-Montané L., Diaz-Gonzalez F., Cátedra-Vallés E.V., Heath K.E. (2021). Identification of the third FGF9 variant in a girl with multiple synostosis-comparison of the genotype:phenotype of FGF9 variants in humans and mice. Clin. Genet..

[44] Wu X.L., Gu M.M., Huang L., Liu X.S., Zhang H.X., Ding X.Y., Xu J.Q., Cui B., Wang L., Lu S.Y. (2009). Multiple synostoses syndrome is due to a missense mutation in exon 2 of FGF9 gene. Am. J. Hum. Genet..

[45] Rodriguez-Zabala M., Aza-Carmona M., Rivera-Pedroza C.I., Belinchon A., Guerrero-Zapata I., Barraza-Garcia J., Vallespin E., Lu M., Del Pozo A., Glucksman M.J. (2017). FGF9 mutation causes craniosynostosis along with multiple synostoses. Hum. Mutat..

[46] Murakami H., Okawa A., Yoshida H., Nishikawa S., Moriya H., Koseki H. (2002). Elbow knee synostosis (Eks): A new mutation on mouse Chromosome 14. Mamm. Genome.

[47] Bashir R.A., Dixit A., Goedhart C., Parboosingh J.S., Innes A.M., Ferreira P., Hasan S.U., Au P.B. (2017). Lin-Gettig syndrome: Craniosynostosis expands the spectrum of the KAT6B related disorders. Am. J. Med. Genet. A.

[48] Mignot C., Moutard M.L., Rastetter A., Boutaud L., Heide S., Billette T., Doummar D., Garel C., Afenjar A., Jacquette A. (2016). ARID1B mutations are the major genetic cause of corpus callosum anomalies in patients with intellectual disability. Brain.

[49] Goos J.A.C., Vogel W.K., Mlcochova H., Millard C.J., Esfandiari E., Selman W.H., Calpena E., Koelling N., Carpenter E.L., Swagemakers S.M.A. (2019). A de novo substitution in BCL11B leads to loss of interaction with transcriptional complexes and craniosynostosis. Hum. Mol. Genet..

[50] Zhao X., Wu B., Chen H., Zhang P., Qian Y., Peng X., Dong X., Wang Y., Li G., Dong C. (2022). Case report: A novel truncating variant of BCL11B associated with rare feature of craniosynostosis and global developmental delay. Front. Pediatr..

[51] Eto K., Machida O., Yanagishita T., Shimojima Yamamoto K., Chiba K., Aihara Y., Hasegawa Y., Nagata M., Ishihara Y., Miyashita Y. (2022). Novel BCL11B truncation variant in a patient with developmental delay, distinctive features, and early craniosynostosis. Hum. Genome Var..

[52] Bostwick B.L., McLean S., Posey J.E., Streff H.E., Gripp K.W., Blesson A., Powell-Hamilton N., Tusi J., Stevenson D.A., Farrelly E. (2017). Phenotypic and molecular characterisation of CDK13-related congenital heart defects, dysmorphic facial features and intellectual developmental disorders. Genome Med..

[53] Gregor A., Sadleir L.G., Asadollahi R., Azzarello-Burri S., Battaglia A., Ousager L.B., Boonsawat P., Bruel A.L., Buchert R., Calpena E. (2018). De Novo Variants in the F-Box Protein FBXO11 in 20 Individuals with a Variable Neurodevelopmental Disorder. Am. J. Hum. Genet..

[54] Schwerd T., Krause F., Twigg S.R.F., Aschenbrenner D., Chen Y.-H., Borgmeyer U., Müller M., Manrique S., Schumacher N., Wall S.A. (2020). A variant in IL6ST with a selective IL-11 signaling defect in human and mouse. Bone Res..

[55] Schwerd T., Twigg S.R.F., Aschenbrenner D., Manrique S., Miller K.A., Taylor I.B., Capitani M., McGowan S.J., Sweeney E., Weber A. (2017). A biallelic mutation in IL6ST encoding the GP130 co-receptor causes immunodeficiency and craniosynostosis. J. Exp. Med..

[56] Lipiński P., Różdżyńska-Świątkowska A., Iwanicka-Pronicka K., Perkowska B., Pokora P., Tylki-Szymańska A. (2022). Long-term outcome of patients with α-mannosidosis—A single center study. Mol. Genet. Metab. Rep..

[57] Strande N.T., Riggs E.R., Buchanan A.H., Ceyhan-Birsoy O., DiStefano M., Dwight S.S., Goldstein J., Ghosh R., Seifert B.A., Sneddon T.P. (2017). Evaluating the Clinical Validity of Gene-Disease Associations: An Evidence-Based Framework Developed by the Clinical Genome Resource. Am. J. Hum. Genet..

[58] Wright C.F., Fitzgerald T.W., Jones W.D., Clayton S., McRae J.F., van Kogelenberg M., King D.A., Ambridge K., Barrett D.M., Bayzetinova T. (2015). Genetic diagnosis of developmental disorders in the DDD study: A scalable analysis of genome-wide research data. Lancet.

[59] Holland P.W., Booth H.A., Bruford E.A. (2007). Classification and nomenclature of all human homeobox genes. BMC Biol..

[60] Wilk K., Yeh S.A., Mortensen L.J., Ghaffarigarakani S., Lombardo C.M., Bassir S.H., Aldawood Z.A., Lin C.P., Intini G. (2017). Postnatal Calvarial Skeletal Stem Cells Expressing PRX1 Reside Exclusively in the Calvarial Sutures and Are Required for Bone Regeneration. Stem Cell Rep..

[61] Opperman L.A. (2000). Cranial Sutures as Intramembranous Bone Growth Sites. Dev. Dyn..

[62] Levi B., Wan D.C., Wong V.W., Nelson E., Hyun J., Longaker M.T. (2012). Cranial suture biology: From pathways to patient care. J. Craniofac. Surg..

[63] Ornitz D.M., Marie P.J. (2015). Fibroblast growth factor signaling in skeletal development and disease. Genes Dev..

[64] Rice D.P., Aberg T., Chan Y., Tang Z., Kettunen P.J., Pakarinen L., Maxson R.E., Thesleff I. (2000). Integration of FGF and TWIST in calvarial bone and suture development. Development.

[65] Kim H.J., Rice D.P., Kettunen P.J., Thesleff I. (1998). FGF-, BMP- and Shh-mediated signalling pathways in the regulation of cranial suture morphogenesis and calvarial bone development. Development.

[66] Harada M., Murakami H., Okawa A., Okimoto N., Hiraoka S., Nakahara T., Akasaka R., Shiraishi Y., Futatsugi N., Mizutani-Koseki Y. (2009). FGF9 monomer-dimer equilibrium regulates extracellular matrix affinity and tissue diffusion. Nat. Genet..

[67] Harada M., Akita K. (2020). Mouse fibroblast growth factor 9 N143T mutation leads to wide chondrogenic condensation of long bones. Histochem. Cell Biol..

[68] Gabriel L.A., Wang L.W., Bader H., Ho J.C., Majors A.K., Hollyfield J.G., Traboulsi E.I., Apte S.S. (2012). ADAMTSL4, a secreted glycoprotein widely distributed in the eye, binds fibrillin-1 microfibrils and accelerates microfibril biogenesis. Investig. Ophthalmol. Vis. Sci..

[69] Bader H.L., Wang L.W., Ho J.C., Tran T., Holden P., Fitzgerald J., Atit R.P., Reinhardt D.P., Apte S.S. (2012). A disintegrin-like and metalloprotease domain containing thrombospondin type 1 motif-like 5 (ADAMTSL5) is a novel fibrillin-1-, fibrillin-2-, and heparin-binding member of the ADAMTS superfamily containing a netrin-like module. Matrix Biol..

[70] Tsutsui K., Manabe R., Yamada T., Nakano I., Oguri Y., Keene D.R., Sengle G., Sakai L.Y., Sekiguchi K. (2010). ADAMTSL-6 is a novel extracellular matrix protein that binds to fibrillin-1 and promotes fibrillin-1 fibril formation. J. Biol. Chem..

[71] Ding L., Cao J., Lin W., Chen H., Xiong X., Ao H., Yu M., Lin J., Cui Q. (2020). The Roles of Cyclin-Dependent Kinases in Cell-Cycle Progression and Therapeutic Strategies in Human Breast Cancer. Int. J. Mol. Sci..

[72] Kipreos E.T., Pagano M. (2000). The F-box protein family. Genome Biol..

[73] Silverman J.S., Skaar J.R., Pagano M. (2012). SCF ubiquitin ligases in the maintenance of genome stability. Trends Biochem. Sci..

[74] Zollino M., Lattante S., Orteschi D., Frangella S., Doronzio P.N., Contaldo I., Mercuri E., Marangi G. (2017). Syndromic Craniosynostosis Can Define New Candidate Genes for Suture Development or Result from the Non-specifc Effects of Pleiotropic Genes: Rasopathies and Chromatinopathies as Examples. Front. Neurosci..

[75] Bjornsson H.T. (2015). The Mendelian disorders of the epigenetic machinery. Genome Res..

[76] Matsuoka K., Park K-a Ito M., Ikeda K., Takeshita S. (2014). Osteoclast-Derived Complement Component 3a Stimulates Osteoblast Differentiation. J. Bone Miner. Res..

[77] Mödinger Y., Löffler B., Huber-Lang M., Ignatius A. (2018). Complement involvement in bone homeostasis and bone disorders. Semin. Immunol..

[78] Wang Z., Li J., Li K., Xu J. (2018). SOX6 is downregulated in osteosarcoma and suppresses the migration, invasion and epithelial-mesenchymal transition via TWIST1 regulation. Mol. Med. Rep..

[79] An C.I., Ichihashi Y., Peng J., Sinha N.R., Hagiwara N. (2016). Transcriptome Dynamics and Potential Roles of Sox6 in the Postnatal Heart. PLoS ONE.

[80] Kyrylkova K., Iwaniec U.T., Philbrick K.A., Leid M. (2016). BCL11B regulates sutural patency in the mouse craniofacial skeleton. Dev. Biol..

[81] Senaratne T.N., Quintero-Rivera F., Adam M.P., Everman D.B., Mirzaa G.M., Pagon R.A., Wallace S.E., Bean L.J.H., Gripp K.W., Amemiya A. (1993). NFIA-Related Disorder. GeneReviews(^®^).

