# Supplementary material for: Review of Recurrently Mutated Genes in Craniosynostosis Supports Expansion of Diagnostic Gene Panels
Source: Genes (Basel). 2023 Feb 28;14(3):615. doi: 10.3390/genes14030615 (PMC10048212; doi:10.3390/genes14030615)
Supplement: Supplementary file 1 [file genes-14-00615-s001.zip › Supplementary Information.pdf]

Supplementary Information

Additional information on genes proposed to reach diagnostic grade in craniosynostosis

ADAMTSL4

ADAMTSL4 is a member of the ADAMTS superfamily of 26 secreted molecules that function as proteases (ADAMTS) or regulatory proteins (ADAMTSL) within the extracellular matrix (ECM) [1]. There are seven ADAMTS-like proteins (ADAMTSL1 through to ADAMTSL6 and PAPLN) that all lack a catalytic zinc metalloproteinase domain alongside a propeptide, and disintegrin-like domain found in all ADAMTS proteases. Evidence suggests that several of the ADAMTSL proteins function as ECM-binding proteins and have been shown to bind fibrillin microfibrils at the cell-matrix interface [2-4]. In support, recessive variants affecting *ADAMTSL4* are a known cause of ectopia lentis, a connective tissue defect owing to abnormal fibrillin microfibrils [5]. A defect in connective tissue assembly is also consistent with the pathogenesis of Marfan syndrome, characterised by skeletal, ocular, and cardiovascular defects, caused by heterozygous missense and loss-of-function variants in fibrillin-1 (*FBN1*) [6, 7] (see below section on *FBN1*).

AHDC1

AHDC1 encodes AT-hook DNA binding motif containing 1, for which the precise function is unknown. The AT-hook binding motif is implicated in DNA or RNA binding [8] and in support, immunofluorescence analyses show widespread expression of the protein across the nucleus [9]. Heterozygous loss-of-function variants in *AHDC1* are associated with Xia-Gibbs syndrome, characterised by intellectual disability, delayed motor development, speech delay, hypotonia, and sleep apnoea [10]. The gene has one coding exon, meaning that stop-gain or frameshift variants resulting in a premature termination codon would be predicted to escape nonsense-mediated decay [11]. Functional evidence suggests that the presence of a truncated protein in patients with Xia-Gibbs syndrome results in nuclear protein aggregates [9]. Recently, a study identified AHDC1 as a transcriptional co-regulator of EWS-ETS fusion proteins in Ewing's sarcoma cells; knockdown of *AHDC1*

showed reduced protein levels of EWS-FLI, and their targets, and subsequent reduced cell growth in Ewing's sarcoma cell lines [12], suggesting a role for AHDC1 in the maintenance of cell proliferation.

ARID1B

AT-rich interactive domain-containing protein 1B (ARID1B) is a DNA-binding subunit of the Brahma-associated factor (BAF) chromatin remodelling complex. ARID1B functions to regulate differentiation of stem cells exiting the cell cycle in postmitotic neurones [13]. Variants in *ARID1B* are the most common cause of intellectual disability and are also associated with Coffin-Siris syndrome [14], with defining features including aplasia or hypoplasia of the distal phalanx, developmental delay, and distinctive craniofacial features. Craniosynostosis has been described in two previous cases of Coffin-Siris syndrome: one with a variant in *ARID1B* [14], and a second with a 2p25 deletion, also encompassing *SOX11* [15].

BCL11B

The BAF Chromatin Remodelling Complex Subunit (*BCL11B*) gene is a key transcription factor with multiple roles, including regulation of early development of the central nervous, immune, and cardiac systems [16]. Previously, knock-out studies of *Bcl11b* in mice demonstrated severe coronal craniosynostosis from embryonic day E16.5 with loss of *Bcl11b* resulting in ectopic expression of *Runx2* and *Fgfr2* and downregulation of *Twist1* [17], identifying aberrant osteogenic differentiation as the likely pathogenic mechanism [7]. Following these reports, a patient was identified with a heterozygous *de novo* variant (c.7C>A; p.(Arg3Ser)) affecting a critical residue involved in binding the RBBP4-MTA1 complex; a murine model of the p.(Arg3Ser) variant at post-natal day 0 also displayed coronal craniosynostosis and reduced anterior fontanelles [18]. Subsequently, six families have been reported with craniosynostosis and heterozygous missense or loss-of-function variants in *BCL11B*, establishing craniosynostosis as a frequent phenotype (~30%) of *BCL11B* variation.

CDK13

Cyclin-dependent kinase 13 (CDK13) is one of 20 ATP-dependent serine-threonine protein kinases that regulate cell-cycle progression and gene expression, which acts by controlling phosphorylation status and activity of splicing regulators [19]. Variants in *CDK13* have been described in patients with developmental delay, intellectual disability, facial dysmorphism, congenital heart defects and seizures [20, 21]. *De novo* heterozygous missense variants are clustered within the protein kinase domain, specifically affecting the ATP and magnesium binding sites; a recurrent variant affecting amino acid 842 has been identified [19]. Documented genotype-phenotype correlations suggest that milder phenotypes are associated with haploinsufficiency of *CDK13*, while more severe presentations are due to dominant-negative effects of missense variants [22].

FBN1

FBN1 encodes fibrillin-1 protein, a microfibril element. Fibrillin-1 is capable of activating transforming growth factor β (TGF-β) which plays an essential role in regulating several early developmental pathways [7, 23]. Specifically for cranial suture biogenesis, TGF-β is upregulated in osteoblasts establishing a marker for growth and differentiation [24]. Additionally, evidence suggests that TGF-β provides an instructive role for the underlying dura mater; signalling interactions have been reported between mesenchymal layers of the coronal suture expressing several TGF-β ligands [24, 25]. Variants in this pathway result in phenotypes associated with abnormal connective tissue assembly (see *ADAMTSL4*), with over 1000 variants reported to be associated with Marfan syndrome [26].

FBXO11

FBXO11 belongs to the F-Box family of proteins that contain a 50 amino acid F-box domain, mediating protein-protein interaction for ubiquitin-mediated proteolysis [27]. There are over 60 different members of the F-Box family which differ in their functional domains; FBXO10 and FBXO11 harbour carbohydrate-binding protein and sugar hydrolase (CASH) domains, which are essential for substrate

recognition [28]. FBXO11 constitutes one subunit of an E3-ubiqitin ligase complex and functions to recognise substrates for degradation [29]. The protein is widely distributed in the cell cytoplasm and nucleus [30] and is believed to be important for the maintenance of genome stability [29]. Variants in *FBXO11* have been implicated in various malignancies, including B-cell lymphoma [31], acute myeloid leukaemia [32] and hepatocellular carcinoma [33]. Recently, *de novo* missense and loss-of-function variants in *FBXO11* have been associated with neurodevelopmental disorders [30, 34]. Functional work suggests that variant substitution within a functional domain of FBXO11 (F-box, CASH or Zinc-finger UBR) does not disrupt the formation of the E3-ubiquitin ligase complex but instead causes abnormal subcellular localisation, forming cytoplasmic aggregates in 50-90% of cells, or reduced expression compared to the wildtype protein [30]. In a subset of missense substitutions affecting the nuclear localisation sequence, an inability to enter the nucleus was identified as the likely pathophysiological mechanism [30].

FGF9

The family of fibroblast growth factors (FGFs) and their receptors (FGFRs) are essential for bone development. In humans, there are 23 FGF ligands (encoded by FGF1-23) which largely exert their pleiotropic effects by binding and activating tyrosine kinase receptors, encoded by four genes (*FGFR1-4*) [35]. Heterozygous missense variants in *FGF9* (including p.(Arg62Gly) and p.(Arg190Thr)) are documented in humans to cause multiple synostoses syndrome 3, owing to a failure of FGF9 to form a homodimer and subsequently a reduced ability to bind its receptor, FGFR3 [36-38].

IL6ST

Members of the cytokine family (including interleukin (IL)-6, IL-11, IL-27, and leukaemia inhibitory factor (LIF)) are known to signal via the common GP130 (Glycoprotein 130) cytokine receptor, and the JAK/STAT signalling pathway. Loss of GP130 in mouse models results in lethality, with mice displaying multiple skeletal abnormalities because of a failure in osteoblast and osteoclast function [39, 40].

IL6ST encodes the GP130 protein and hence transduces IL11-mediated cytokine signalling. Perturbations in cytokine signalling have been shown to cause anomalies of osteoclast differentiation. For example, the absence of the localised resorption of jawbone, a prerequisite for secondary dentition, was shown to delay onset of dentition in homozygous mutants of *Il11ra*$^{-/-}$ [41], further corroborating a defect in osteoclast differentiation as the main pathological mechanism. In line with defects in other members of the cytokine family, it is believed that recessive variants in *IL6ST* would cause skeletal abnormalities (including craniosynostosis), alongside a wider immunodeficiency defect owing to the number of different ligands binding GP130 [42].

KAT6B

The lysine acetyltransferase 6 (KAT6) complex acetylates histone H3 and non-histone proteins to control chromatin structure and gene transcription [43]. KAT6 proteins have been shown to be essential for normal craniofacial development, with mice homozygous for complete loss of *Kat6b* displaying low body weight and abnormalities of the occipital and parietal bones, alongside irregular sutures. Notably, craniosynostosis was not observed suggesting a species difference [44]. Additionally, KAT6B is a known inducer of *RUNX2* expression, the master regulator of osteogenesis, with experimental evidence showing that overexpression of KAT6B promoted proliferation of primary chondrocytes and bone formation [45]. Last, KAT6B is an important regulator of stem cell maintenance [46]. Loss of *KAT6B* resulted in cells displaying a more compact chromatin organisation, since lysine modifications allow DNA accessibility, and subsequently an impaired ability to interact with OCT4 and NANOG transcription factors (essential factors in the maintenance of undifferentiated stem cells) [47]. As such, it is likely that craniosynostosis would occur owing to a primary failure to maintain a population of undifferentiated sutural stem cells, in favour of enhanced osteogenic differentiation driven by *RUNX2*.

MAN2B1

The mannosidase alpha class 2B member 1 (MAN2B1) gene encodes a lysosomal alpha-D-mannosidase which functions to degrade N-linked glycoproteins, maintaining cellular homeostasis. The absence of lysosomal alpha-D-mannosidase activity results in the accumulation of partially degraded oligosaccharides in lysosomes, resulting in alpha-mannosidosis with variable phenotypes. For example, recessive variants in *MAN2B1* were shown to cause intellectual disability, hearing impairment, motor function imbalance, and craniofacial and musculoskeletal anomalies [48, 49]. Missense and loss-of-function variants are distributed across the protein, but with a notable genotype-phenotype correlation; loss-of-function or substitutions affecting the active site (amino acid 196; nucleophile) were noted to cause protein destabilisation and lead to severe phenotypes (profound mental retardation, severe dysostosis multiplex, and reduced life expectancy), whereas variants affecting positions away from the active site caused milder phenotypes (moderate intellectual disability, hearing impairment, mild dysostosis and survival to adulthood) [48]. Additionally, mutations which caused incorrect protein folding were shown to localise to the endoplasmic reticulum, while missense variants allowed sufficient protein folding to localise to the lysosome and conferred residual alpha-D-mannosidase activity [48].

MASP1

MASP1 encodes the mannan-binding lectin serine protease-1 which functions in the lectin pathway of complement. The gene is comprised of 18 exons; alternative splicing of exons 9-18 generates three isoforms (MASP-1, MASP-3 and MAp44) that differ by the presence or absence of the serine protease domain (SPD) [50, 51]. MASP-1 and MASP-3, which contain the SPD encoded by exons 13-18 or exon 12, respectively, are enzymes involved in the C3 pathway of complement, while Map44 has no enzymatic function [50]. Recessive, missense and loss-of-function variants in *MASP1* are associated with 3MC syndrome (comprising four disorders: Michels syndrome, Malpuech syndrome, Mingarelli syndrome and Carnevale syndrome) [52]. 3MC syndrome is characterised by postnatal growth

retardation, facial dysmorphism, hearing loss, genital anomalies, and skeletal malformations, with craniosynostosis described in 9/30 (30%) individuals with variants in *MASP1* [53]. Alongside *MASP1*, variants in other genes within the complement pathway (*COLEC10* and *COLEC11*) also manifest in 3MC syndrome. Craniosynostosis is reported in 41% of individuals with recessive *COLEC11* variants [53]. Genetic ablation of *MASP1* using ATG and splice morpholinos in zebrafish resulted in craniofacial cartilage defects, which was phenocopied in the *COLEC11* mutants [54]. Additionally, an abnormal distribution of neural crest cells was identified across the midline of the hindbrain in mutants compared to controls [54], consistent with a defect in neural crest cell boundary formation and integrity [7].

NFIA/ NFIX

NFIA and *NFIX* encode two members of the nuclear factor 1 family of transcription factors, which play key roles in activating various embryonic differentiation pathways. Heterozygous truncating variants or intragenic deletion of *NFIA* are associated with brain abnormalities, intellectual disability, and occasionally, urinary tract defects [55, 56]. Studies of *Nfia* knockout in mice found that over 95% of neonates died within 2-weeks of birth, displaying an absence of the corpus callosum and signs of early hydrocephalus. Any surviving homozygotes lacked a corpus callosum, displayed a dome-shaped head, showed neurological defects, and had low fertility [57]. *Nfia* heterozygous littermates ($Nfia^{+/-}$) displayed an absence of the corpus callosum alongside renal abnormalities [58], supporting a key role for this gene during early vertebrate development. Similarly, absence of *Nfix* in mice resulted in hydrocephalus, partial agenesis of the corpus collosum, and delayed ossification [59]. A defect in cell differentiation of astroglia cells of the midline has been proposed as the likely pathophysiological mechanism behind *NFI*-related disorders [56, 60]. As such, it is possible that craniosynostosis is a secondary phenotype to primary disorders of the brain associated with *NFIA* and *NFIX* variants.

PRRX1

PRRX1 encodes the mammalian paired-related homeobox 1 transcription factor. The homeobox class contains a highly conserved 60 amino acid (aa) sequence known as the homeodomain, comprising an N-terminal arm and three alpha-helices which mediate DNA-binding. *PRRX1* consists of 5 coding exons; alternative splicing of exon 4 generates two distinct protein isoforms, *PRRX1a* (which contains an additional domain for transcriptional activation, known as the OAR domain [61]) and *PRRX1b*. Monoallelic and biallelic missense and loss-of-function variants in *PRRX1* are associated with a rare and lethal disorder, agnathia-otocephaly, characterised by the underdevelopment of the mandible [62-65]. However, recent evidence suggests that haploinsufficiency of *PRRX1* is more commonly associated with craniosynostosis [66].

SOX6

SOX6 is a member of the SRY-related HMG-box-containing (SOX) genes, which encode transcription factors controlling cell fate and differentiation. Variants in several of the SOX genes are associated with intellectual disability, amongst other features. For example, *SOX11* and *SOX4* haploinsufficiency cause a Coffin-Siris-like syndrome, characterised by intellectual disability, growth deficiency, microcephaly, and dysmorphic features [67]. Half of the 20 *SOX* genes have been associated with developmental defects, resulting in coining of the term *SOXopathies* to describe the phenotypic spectrum of variants in these genes which largely comprise intellectual disability, skeletal dysmorphism, and cardiovascular abnormalities [67]. *SOX6* consists of 16 exons for which alternative splicing generates 4 different isoforms. All isoforms encode two coiled-coil domains (CC1 and CC2) and an HMG domain; variants have been described across the length of the protein and do not cluster into a mutational hot spot [68]. The majority of described *SOX6* variants are predicted to abolish protein function, confirming *SOX6* haploinsufficiency in the aetiology of neurodevelopmental disorder, craniosynostosis, and osteochondromas [68]. Genome-wide association studies identify *SOX6* as a susceptibility locus for osteoporosis [69, 70]; knockdown of *SOX6* using siRNA reduced

chondrogenesis and osteogenesis, suggesting a defect in stem cell differentiation as the likely pathophysiological mechanism.

Supplementary Methods

Sequencing of a cohort of patients with genetically unsolved craniosynostosis

A cohort of 617 individuals without a genetic basis to their craniosynostosis were screened for variants in 42 genes (detailed in table S2). Primers were designed to screen all coding exons of the canonical transcript. Individual libraries for capture-based resequencing were prepared using the 'Twist Bioscience' kits for the enzymatic fragmentation and universal adapter system (protocol version 11 (September 2019) Rev1), or the 'xGenTM DNA Library Prep EZ Kit' and protocol (IDT, protocol version 1 (December 2021)). DNA was fragmented to ~200 bp, before adding adapters and indexing primers, and subsequently analyzed using broad-range Qubit and D1000 TapeStation reagents (average fragment size of 330 bp). The prepared libraries were pooled to a total of 6 μg of DNA and between 32-40 samples per hybridization capture reaction. The hybridization reactions were carried out at 65°C for 16 hours. After hybridization, the pooled libraries were washed and post-capture PCR was performed following manufacturer's protocol (IDT xGen hybridization capture of DNA libraries for NGS target enrichment, 7 PCR cycles were used), for a panel containing 2054 probes. The amplified capture reactions were washed with beads before quantification and validation using high-sensitivity (HS) qubit reagents and HS D1000 TapeStation, before next generation sequencing analysis using Miseq v3 (150-cycle, MRC WIMM Sequencing Facility) and data analysis using amplimap software [71]. Variants were filtered based on allele frequency ($\leq$ 0.000045 for dominant mode of inheritance (as per previously reported frameworks [72, 73]) or $\leq$ 0.01 for recessive variants) and CADD score ($\geq$ 20 or missing), before screening manually for likely pathogenicity.

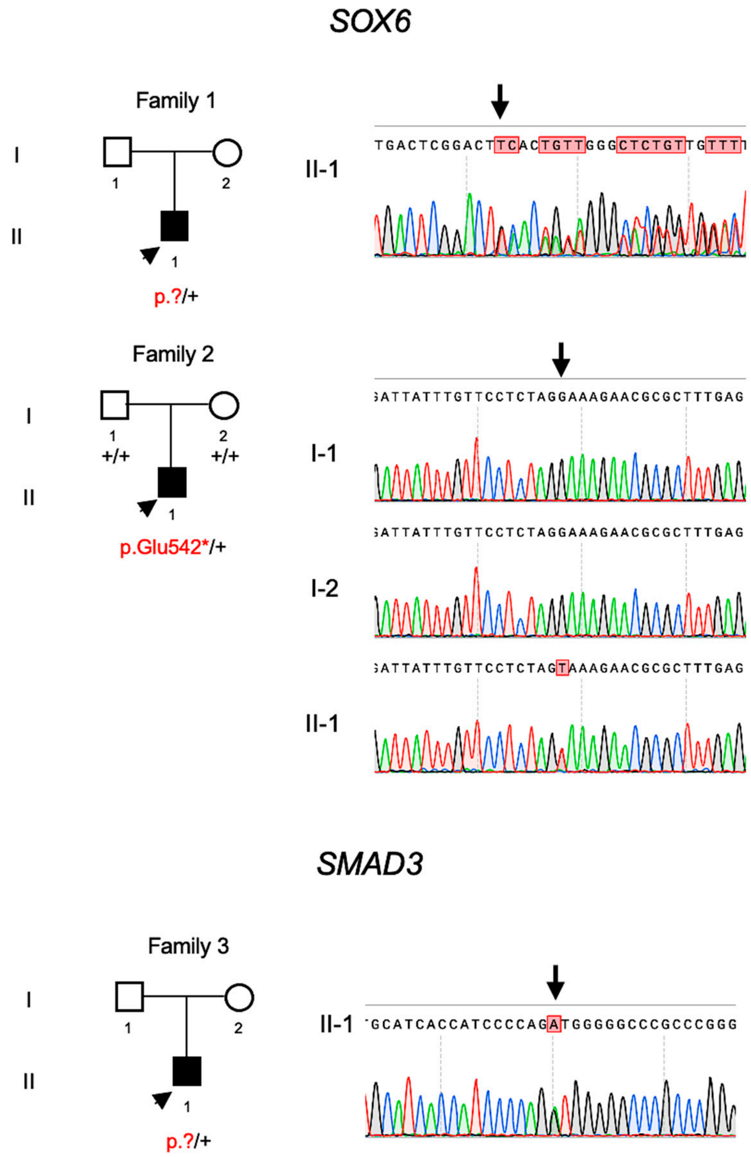

Figure S1 Pedigree figures and dideoxy-sequencing confirmation of *SOX6* (NM_033326.3) and *SMAD3* (NM_005902.4) variants. Top, Family 1 with an identified 23 bp deletion, c.436_445+13del; p.(?), in *SOX6* (primers: forward = 5'-GTCCTACTAAGCATCATATACTGTCCTCAG-3', reverse = 5'-GAGAAGGTAGATGTGGGATGTTG-3'); parental samples were not available to assess inheritance. The proband presented with multisuture craniosynostosis, low anterior hairline, synophrys, small mouth, and hydrocephalus with visual loss secondary to raised intracranial pressure. Middle, Family 2 with a *de novo* stop-gain, c.1624G>T; p.(Glu542*), in *SOX6* (primers: forward = 5'-GAGCAAATCCGACAGAGAGAAGATATGCATAG-3', reverse = 5'-CGTAGTTGAAGAGTGACATTTCCTTTGC-3'). The proband presented with multisuture craniosynostosis, severe allergic enteropathy associated with hyperphosphataemic rickets, and developmental delay. Below, Family 3 with an identified splicing variant in *SMAD3*: c.206+1G>A; p.(?) (primers: forward = 5'-GCTGGAAGAAGGGCGAGCAGAA-3', reverse = 5'-CCTCGGGCTCTGATCTTTGCAAATC-3'); parental samples were not available to assess inheritance. The proband presented with non-syndromic coronal synostosis. For all sequencing traces, the black arrow denotes the position of the nucleotide change; the red box highlights any residues that differ from the wildtype sequence.

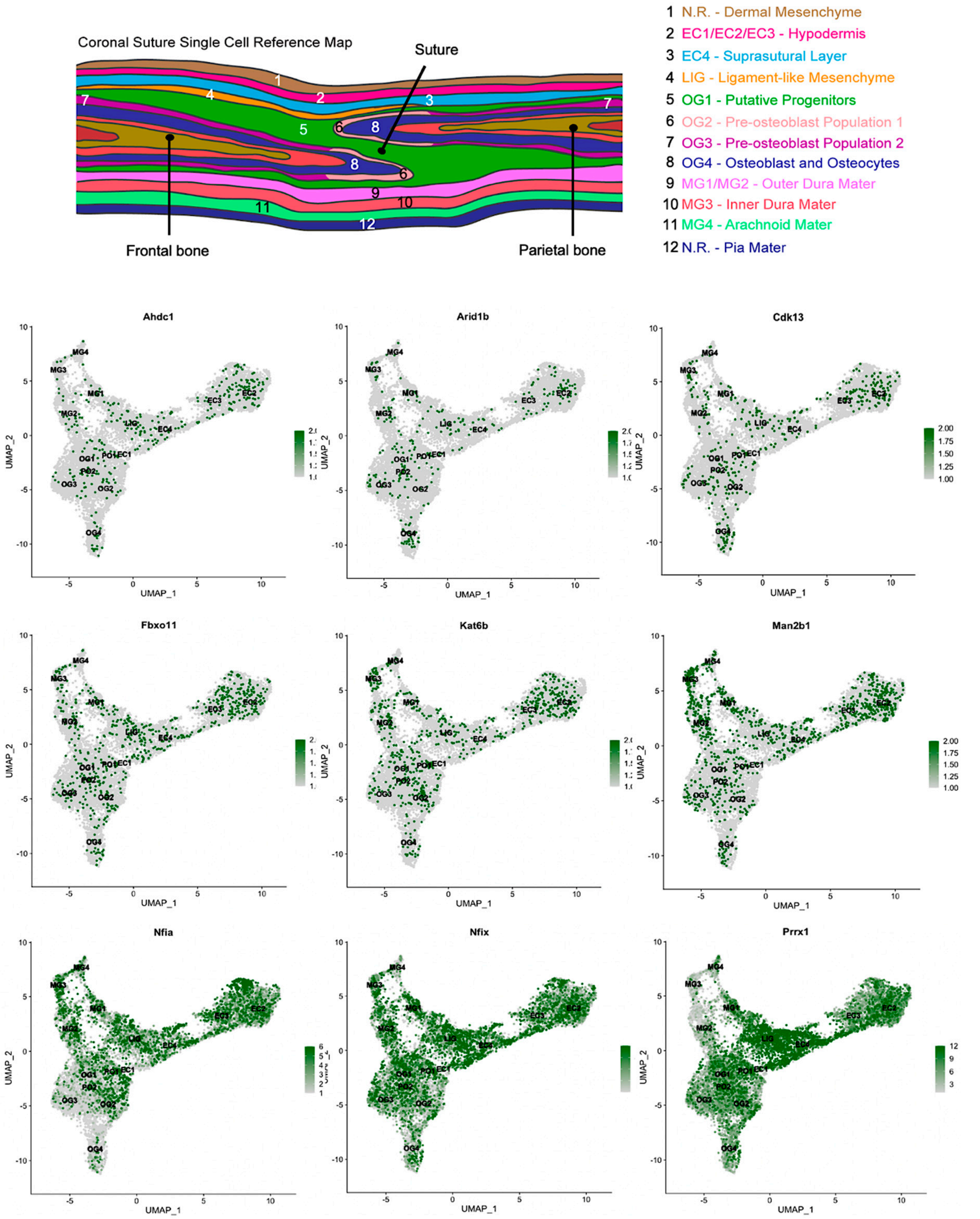

Figure S2 Genes displaying widespread expression across cell clusters. Top, a reference map displaying the locations of the cell populations of the mouse E15.5 – 17.5 coronal suture corresponding to cell clusters on the UMAP plots below. Below, feature plots showing generalised gene expression across the coronal suture for nine genes. Expression of *Nfia*, *Nfix* and *Prrx1* is notably higher than the other six genes. These data are available at FaceBase (https://www.facebase.org/) with the Record ID: 4-6J38. A Seurat object file (E15_17_composite_mesenchyme_bone_subset.rds) is available for the analysis of suture single cell data with relevant clustering shown. Individual gene expression patterns can be visualized using Seurat (https://satijalab.org/seurat/).

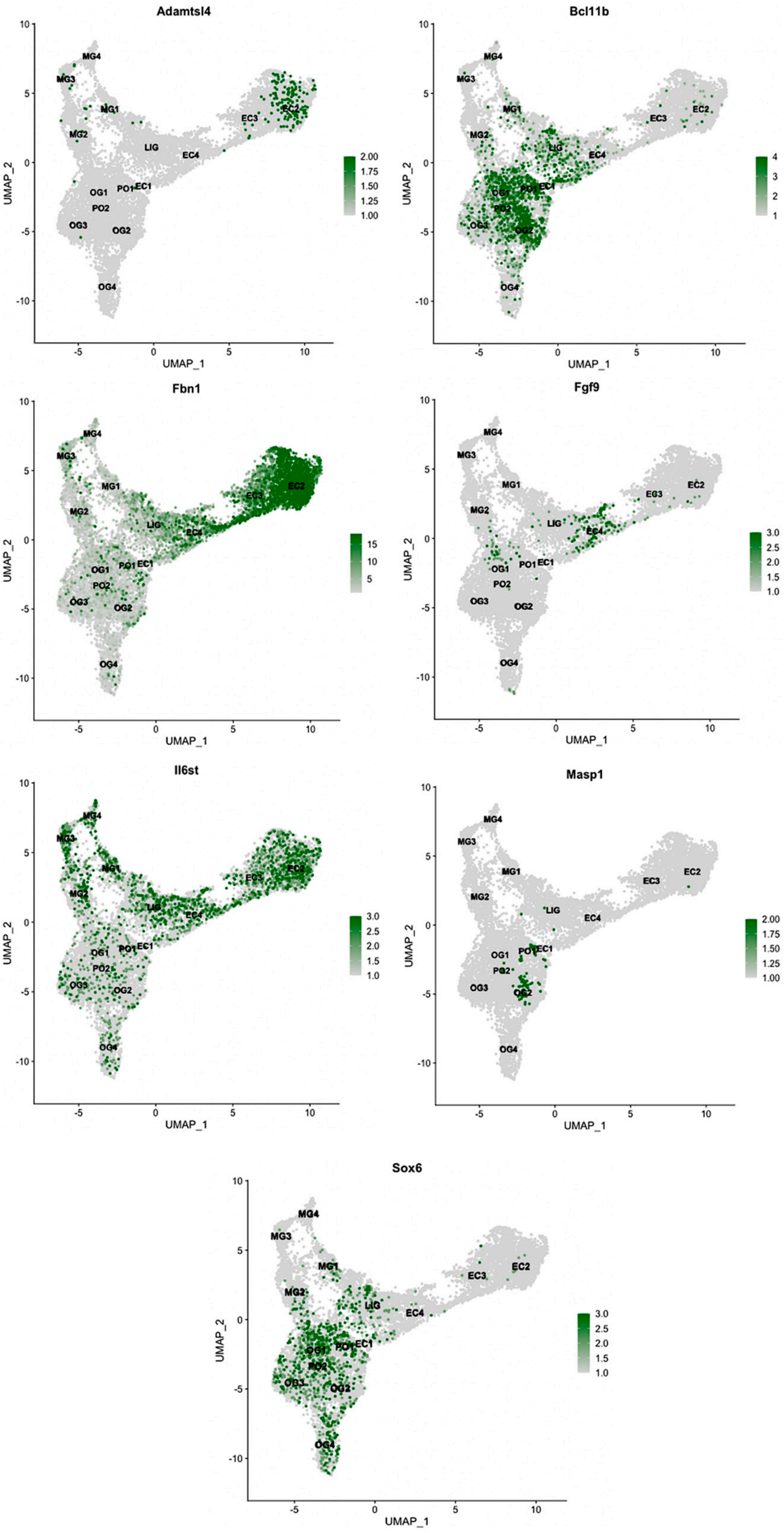

Figure S3 Coronal suture single cell transcriptomic map and specific expression patterns of seven genes. Feature plots of genes identified in this study with specific expression patterns in populations of cells in the mouse E15.5 – 17.5 coronal suture. Refer to Figure S2 for cluster definitions and spatial locations.

Table S1 Criteria for a diagnostic-grade Green gene on PanelApp

**PanelApp Criteria for a Diagnostic Grade 'Green' Gene for Rare Disease**
A. Plausible disease-causing variant (recurrent *de novo* variants which affect gene function or, if they are rare, they must be fully penetrant and never (or very rarely) seen in controls).<br>B. Within, or predicted to affect a functional region of a gene (interpretable function affecting an open reading frame in a protein-coding gene, miRNA stem or loop), or within, affecting or encompassing a cis-regulatory element which would affect the expression of a single gene.<br>C. Identified in three or more independent cases/families with the phenotype (the normalised rare disease category).
If the variant is identified in two unrelated cases or families:<br>A. Additional convincing bioinformatic of functional evidence to support variant pathogenicity (for example, evidence of an animal model which phenocopies the human presentation). Intermediate penetrance genes should not be included.<br>B. No evidence exists that contradicts the role of the gene in the specified phenotype

https://panelapp.genomicsengland.co.uk/media/files/PanelApp_User_Guide.pdf, accessed 12th December 2022

Table S2 Whole exome, genome, or panel-based analyses of patients with craniosynostosis between 2018 – 2022

Reference	Number of probands screened	Sequencing technology	Phenotypes included in the screen
Australia/ New Zealand (Lee et al., 2018) [74]	309	20-gene panel (*ACTB, ACTG1, ALX1, ALX3, ALX4, EFNA4, EFNB1, ERF, FGF10, FGFR1, FGFR2, FGFR3, FREM1, IL11RA, MID1, MSX2, POR, RECQL4, RUNX2, TCF12*)	Non-syndromic coronal synostosis (n = 100, 33%); Saethre-Chotzen syndrome (n = 24, 8%); single suture synostosis (n = 33, 11%); multi-suture craniosynostosis (n = 40, 13%); other syndromic craniosynostosis (n = 62, 20%); suture not specified (n = 25, 8%).<br><br>A subset of 233 individuals within this screen were previously tested for variants in exon 7 of *FGFR1*; exons 8, 10 and 11 of *FGFR2*; and exons 7 and 10 of *FGFR3*. If Saethre-Chotzen syndrome was suspected, exon 1 of *TWIST1* was also screened.
Seattle (Clarke et al., 2018) [75]	397	RNA-Sequencing. Patients were screened for variants in genes (n = 61) associated with syndromic forms of craniosynostosis.	Single suture craniosynostosis: sagittal (n = 201, 51%), metopic (n = 94, 24%), coronal (n = 81, 20%), lambdoid (21, 5%). Any patient with variants in *FGFR1, FGFR2, FGFR3, TWIST1, EFNB1*, and *MSX2*, or who displayed chromosomal rearrangements, were excluded from analysis.
Scandinavia (Topa et al., 2019) [76]	100	63-gene panel *(ACTB, ACTG1, ADAMTSL4, ALX3, ALX4, AXIN2, CCBE1, CD96, CDKN1C, CHST3, COLEC11, CYP26B1, EFNA4, EFNB1, ERF, FBN1, FGF9, FGFR1, FGFR2, FGFR3, FLNA, FREM1, GDF5, GLI3, GPC3, IFT122, IFT43, IGF1R, IHH, IL11RA, JAG1, KDM6A, KMT2D (MLL2), KPTN, KRAS, LRP2, MASP1, MCPH1, MED12, MEGF8, MSX1, MSX2, NOG, NOTCH2, NSD1, POR, PTCH1, PTPRD, RAB23, RECQL4, RUNX2, SH3BP2, SKI, SOX6, SPECC1L, TCF12, TCOF1, TGFBR1, TGFBR2, TWIST1, WDR19, WDR35*, ZEB2)	Syndromic craniosynostosis (78% of the cohort), predominately coronal synostosis.

Yale<br>(Timberlake et al., 2019) [77]	12	Whole exome	Synostosis (n = 4, 25%), metopic (n = 6, 50%), lambdoid (n = 1, 8%), multi-suture (n = 1, 8%). All patients were syndromic.
Japan<br>(Suzuki et al., 2020) [78]	51	Whole exome	51 patients recruited with trigonocephaly and their parents. Predominantly a screen of patients with neurodevelopmental disorders.
Korea<br>(Yoon et al., 2020) [79]	110	34-gene panel (unable to access complete gene list)	Syndromic (n = 40, 36%), non-syndromic (n = 70, 64%). Suture fusion: sagittal (n = 36, 33%), unicoronal (n = 22, 20%), bicoronal (n = 13, 12%), unilambdoid (n = 14, 13%), metopic (n = 9, 8%), multi-suture (n = 16, 15%).
China<br>(Wu et al., 2021) [80]	201	17-gene panel (*EFNB1, ERF, FGFR1, FGFR2, FGFR3, IFT43, MSX2, POR RAB23, RECQL4, SKI SMAD3, TCF12, TGFB2, TGFBR1, TGFBR2, TWIST1*)	Syndromic (n = 85, 42%), non-syndromic (n = 99, 49%), not classified (17, 8%).
Saudi Arabia<br>(Alghamdi et al., 2021) [81]	28	Whole exome	Syndromic (n = 6, 21%). Complex craniosynostosis (n = 14, 50%). Single suture fusion: metopic suture (n = 6, 21%), unilateral coronal (n = 4, 14%), sagittal synostosis (n = 4, 14%).
100kGP<br>(Hyder et al., 2021) [82]	114	Whole genome	Syndromic (n = 82, 72%) and non-syndromic (n = 32, 28%). Suture fusion: sagittal (n = 18, 16%), unicoronal (n = 17, 15%), metopic (n = 13, 11%); bicoronal synostosis (n = 11, 10%); uni- or bi-lateral lambdoid synostosis (n = 3, 3%); multi-suture (n = 41, 36%); suture not specified (n = 11, 10%).
Norway<br>(Tønne et al., 2021) [83]	381	72-gene panel (*ALPL, ALX3, ALX4, ASXL1, ATR, BMP4, CCBE1, CDC45, CEP120, COLEC11, CTSK, CYP26B1, DPH1, EFNA4, EFNB1, ERF, ESCO2, FAM20C, FAM58A, FBN1, FGFR1, FGFR2, FGFR3, FREM1, GLI3, GNAS, GPC3, GTF2E2, HNRNPK, HUWE1,*	Syndromic (n = 104, 27%), non-syndromic (n = 277, 73%).

		IDS, IDUA, IFT122, IFT140, IFT43, IHH, IL11RA, IMPAD1, IRX5, JAG1, KAT6A, KMT2D, KRAS, LMX1B, LRP5, MASP1, MEGF8, MSX2, MYH3, P4HB, PHEX, POR, RAB23, RECQL4, RSPRY1, RUNX2, SCARF2, SH3PXD2B, SKI, SMO, SON, SPECC1L, STAT3, TCF12, TGFBR1, TGFBR2, TMCO1, TWIST1, WDR19, WDR35, ZEB2, ZIC1)	
China (Chen et al., 2022) [84]	264	17-gene panel on all 264 individuals (*EFNB1, ERF, FGFR1, FGFR2, FGFR3, IFT43, MSX2, POR RAB23, RECQL4, SKI SMAD3, TCF12, TGFB2, TGFBR1, TGFBR2, TWIST1*), and whole-exome sequencing (n = 102, 39%)	Syndromic (n = 163, 62%) and non-syndromic (n = 101, 38%). Suture fusion: sagittal (n = 57, 22%), metopic (n = 5, 2%), uni- and bi-lateral coronal (n = 89, 34%), uni- and bi-lateral lambdoid (n = 15, 6%), multi-suture synostosis (n = 67, 25%), suture not specified (n = 31, 12%)
Norway (Tønne et al., 2022) [85]	10	Whole exome	All patients with syndromic craniosynostosis that were negative in the previous Tønne screen [83]
Yale (Timberlake et al., 2023) [86]	25	Whole exome	Sporadic lambdoid synostosis (n = 18, 72%), familial lambdoid synostosis (n = 7, 28%). Unilateral synostosis (n = 20, 80%), bilateral lambdoid synostosis and sagittal synostosis (n = 4, 16%), bicoronal and right lambdoid synostosis (n = 1, 4%)
Oxford (Tooze et al., 2022) [87]	617	42-gene panel (*ANKRD28, ALPL, ALX4, AXIN2, BCOR, DHRS3, DUSP6, EPHA4, ERF, FGF3, FGF4, FGF9, GDF6, GNAS, HSP90AA1, IL11, LMX1B, MSX2, NAA25, NELL1, NFIA, PDGFRA, PITX2, PRRX1, PTCH1, PTHLH, RNU12, SIX2, SLC12A2, SMAD2, SMAD3, SMURF1, SOX6, SOX11, SPRY1, SPTBN1, TFCP2, TGFBR1, TGFBR2, TFAP2B, ZIC1, ZNF462*)	Non-syndromic (n = 454, 74%) and syndromic (n = 163, 26%). Suture fusion: sagittal (n = 148, 24%), metopic (n = 130, 21%), uni- and bi-lateral coronal (n = 217, 35%), uni- and bi-lateral lambdoid (n = 15, 2%), multi-suture synostosis (n = 91, 15%), suture not specified (n = 16, 3%)

Table S3 PanelApp Amber and Red genes and updated evidence for their association with craniosynostosis.

Gene	Current Panel	Literature	Suggested Updated Status.
CHD5	Amber	• Craniosynostosis was identified in 3/7 patients with variants in *CHD5.* Variants included: patient 1 (c.577C>T; p.(Arg193Trp), inherited) displayed sagittal craniosynostosis, patient 4 (c.940G>T; p.(Glu314*); inherited) had metopic craniosynostosis, and patient 5 (c.1279G>A; p.(Glu427Lys), *de novo*) was diagnosed with trigonocephaly. Patient 1 has one affected cousin (neurodevelopmental disorder, speech delay, craniosynostosis). Both parents are unaffected, but the aunty (mother's sister) has speech delay/ neurodevelopmental disorder. Patient 4 inherited the variant from a syndromic father (dyslexia/borderline intellectual disability, but no craniosynostosis); their sibling has suspected craniosynostosis [88]. This paper is already reported in PanelApp. All variants are predicted to affect functional domains (CHDNT (Arg-193), PHD finger (Glu-427)).	Amber. Craniosynostosis is only confirmed in two families.
ESCO2	Amber	• Whole-exome sequencing identified two homozygous inactivating variants: a previously described c.1131+1G>A; p.(Arg228fs*17) transition in one patient with craniosynostosis and an unreported deletion, c.417del; p.(Lys139Asnfs*6), in a second patient with coronal synostosis [89]. The c.1131+1G>A variant has been previously reported as a pathogenic for Roberts and Baller-Gerold Syndromes.	Amber. No further evidence to contribute compared to what is already detailed on PanelApp.
FGF10	Amber	• Screening of the Australian and New Zealand cohort of 233 individuals with craniosynostosis identified two heterozygous variants associated with LADD syndrome: c.366del; p.(Val123Serfs*10) and c.433G>T; p.(Glu145*) [74].	Amber. No further evidence to contribute compared to what is already detailed on PanelApp.
MASP1	Amber	• A *MASP1* variant was identified in a patient with craniosynostosis: c.356G>A; p.(Arg119Gln). The variant was inherited from a clinically unaffected parent. The patient also harboured a variant in *MEGF8* [75].<br>• A review of 3MC syndrome suggests that *MASP1* variants manifest in craniosynostosis in 31% of patients [53].<br>• A review by Atik and colleagues suggests a prevalence of 27.2% [50].<br>• Two additional cases are already detailed on PanelApp.	Green. Multiple reports, predominantly in Durmaz et al [53].

NFIA	Amber	• Two *de novo* variants were detected in *cis* (c.124A>T; p.(Lys42*), and c.250C>T; p.(Arg84*)) in a patient with metopic CRS, hydrocephalus, thin corpus callosum, developmental delay, autism, macrocephaly, supernumerary teeth, reduced vision, long face, hypertelorism, short nose, long philtrum, micrognathia, low-set ears [85].<br>• A variant was identified in the Chinese cohort: c.2346_2352del; p.(Ser784Cysfs*59) [84].<br>• Within the Norwegian cohort a 1p32-p31 deletion (g.53675707_ 66644963del; 13Mb) has been associated with intrauterine growth restriction, developmental delay, preaxial polydactyly, inguinal hernia, short stature, corpus callosum agenesis, optic nerve hypoplasia, thoracic hypoplasia, hearing loss, microphthalmia, micrognathia, dysplastic ears [83].<br>• A 1p32-p31 deletion syndrome was confirmed in an individual within the Korean cohort displaying estropia, hypoplastic corpus callosum, chiari 1 malformation, hearing impairment, congenital tooth defects, mild mental retardation and craniosynostosis. The deletion was confirmed to be 7765 kb [79].	Green. This gene is already reviewed in PanelApp to suggest an update; currently the gene is still amber.
PJA1	Amber	• A recurrent missense variant in *PJA1* has been described in seven patients from five unrelated families with neurodevelopmental disorder and trigonocephaly. The hemizygous missense variant c.1126C>T; p.(Arg376Cys) is localised downstream from the nuclear localization signal (NLS) and upstream from the RING domain [78]. *Pja1* knock-in mice carrying p.(Arg365Cys) (equivalent to human p.(Arg376Cys)) showed a significant decrease in PJA1 protein, suggesting a loss-of-function effect [78]. The hemizygous variant is also present in 4 individuals in gnomAD (out of ~200,000) and identified in individuals with the same haplotype (suggesting a founder effect).<br>• Duplication of *EFNB1,* but also includes *PJA1*, has been described in a family with dysmorphic features, short stature, and mild developmental delay [90].	Amber. The recurrent missense variant is already detailed in PanelApp. The deletion case is likely attributable to deletion of *EFNB1* and therefore adds no further evidence.
PPP1CB	Amber	• A Brazilian boy was described with a p.(Pro49Arg) missense variant in *PPP1CB*. The individual presented with sparse and thin hair, similar to the typical ectodermal findings observed in Noonan syndrome-like disorders, alongside sagittal and coronal craniosynostosis [91].	Amber. Further cases required.
PRRX1	Amber	• Fourteen patients from 17 families with craniosynostosis were found to harbour rare/novel heterozygous variants in *PRRX1,* predicting a missense substitution or loss of function of the homeodomain [66]. Immunofluorescence analyses indicate an inability of the mutant protein to enter the nucleus as the pathogenic mechanism [unpublished].	Green. Recent evidence shows that 17 families have rare heterozygous variants in *PRRX1*.

		The majority of variants are inherited but within this cohort three are confirmed *de novo*.	
RNU12	Amber	• CDAGS syndrome is a rare congenital disorder characterized by craniosynostosis, delayed closure of the fontanelles, cranial defects, clavicular hypoplasia, anal and genitourinary malformations, and skin manifestations. Whole exome sequencing identified a recurrent rare variant (NC_000022.10: g.43011402C>T) in five patients with CDAGS syndrome from four distinct families. This variant affects a highly conserved nucleotide within the precursor U12 snRNA 3' extension. All patients harboured a second variant on the other allele which disrupted secondary structure. Craniosynostosis was noted in three of the families (family 1: bilateral coronal synostosis, family 3: coronal synostosis, and family 4: brachycephaly) [92].	Amber. Identified in three unrelated families. However, this is not a protein coding gene and an Ensembl ID is required before promotion to Green. No further cases to contribute.
SEC24D	Amber	• A patient was described with short stature, craniofacial abnormalities including ocular proptosis, marked frontal bossing, midface hypoplasia, and micrognathia, consistent with a diagnosis of Cole-Carpenter syndrome. He had low-bone mineral density and basilar impression. Whole exome sequencing analysis identified biallelic variants in *SEC24D* (encoding, p.(Arg484*) and p.(Arg313His)) in the patient [93].<br>• A child with Cole-Carpenter syndrome was identified with compound heterozygous variants in *SEC24D* – a nonsense variant (c.613C>T; p.(Gln205*)) and a missense variant (c.3044C>T; p.(Ser1015Phe)) [94].<br>• Two families were reported with autosomal recessive compound heterozygous variants in *SEC24D*: in family 1, c.2723G>A; p.(Cys908Tyr) and c.2842T>C; p.(Ser948Pro); in family 2, c.938G>A; p.(Arg313His), and c.875C>T; p.(Pro292Leu). Widened sagittal suture is reported for one individual [95].	Amber. Craniosynostosis is not a clear feature.
SHOC2	Amber	• A child with Noonan syndrome, characterised by short stature, developmental delay, and severe craniosynostosis involving right coronal, bilateral lambdoid, and sagittal sutures has been described with a *de novo* mutation in exon 1 of *SHOC2* (c.4A>G; p.(Ser2Gly)) [96].	Amber. Single case study, no further evidence to contribute to PanelApp.
SMAD3	Amber	• A review of genes/variants associated with Loeys-Dietz syndrome (LDS) indicates that *SMAD3* variants are associated with craniosynostosis, but no reference is given [97].<br>• A report of four families with LDS and *SMAD3* variants suggests that 3/9 individuals from three different Italian families presented with dolichocephaly among other phenotypes.	Amber. The only *bona fide* variant previously reported is a homozygous variant in a patient with

		Four of these families reported *SMAD3* variants, although it is unclear which of these individuals had craniosynostosis and whether it has been confirmed by CT scan: c.1247C>T; p.(Ser416Phe), c.1009+1G>A; p.(Arg292_Gly337del), c.803G>A; p.(Arg268His), c.862_871+8del; p.(Arg288Glufs*50) [98].<br>• A report of a biallelic splicing variant in *SMAD3* (c.532+2T>A; p.(?)) in a patient with LDS – dolichocephaly is mentioned but not clear that craniosynostosis is radiologically confirmed [99].<br>• One additional patient identified with a heterozygous splicing variant in *SMAD3* [this study] – parental samples were not available for testing.	LDS. We contributed one additional patient from the resequencing analysis, but more cases are required to update PanelApp status.
SOX6	Amber	• There are two individuals with loss of function variants identified in a resequencing analysis of 617 individuals: c.436_445+13del; p.(?), and c.1624G>T; p.(Glu542*) [this study].<br>• Sagittal craniosynostosis with scaphocephaly was observed in two unrelated affected individuals with *de novo SOX6* variants (c.242C>G; p.(Ser81*) and c.277C>T; p.(Arg93*)), and oxycephaly with synostosis of the sagittal, metopic, and coronal sutures was observed in a third unrelated individual with a deletion of exons 5 – 7 [68, 82].<br>• In the Chinese cohort, one *de novo* variant was identified in an individual with non-syndromic uni-lambdoid synostosis: c.1243C>T; p.(Gln415*) [84].<br>• An additional patient was described with a balanced translocation affecting *SOX6*. He presented with brachycephaly, proptosis, midfacial hypoplasia, and low set ears [100].	Green. There are 7 independent families with loss of function variants in *SOX6* and craniosynostosis.
ABCC9	Red	• No further evidence.	Red.
ADAMTSL4	Red	• One patient with right coronal synostosis and bilateral ectopia lentis has been shown to harbour a homozygous deletion in exon 6 of *ADAMTSL4* (c.767_786del), encoding a premature termination codon, p.(Gln256Profs*38). The proband's mother, father and one sibling are heterozygous carriers of the variant [101].<br>• Two reported cases of craniosynostosis with ectopia lentis, each harbouring recessive variants in *ADAMTSL4*. The first patient presented with bilateral coronal craniosynostosis, retrusion of the frontal bones, moderate retrusion of the surpraorbital rims, anteroposterior enlargement of the anterior fontanelle with moderate supraorbital rim retrusion, and mild proptosis associated with a heterozygous 20 base pair deletion (c.767_786del; p.(Gln256Profs*38)) and a splice site frameshift deletion (c.2177+3_2177+6delGAGT; p.(?)) (confirmed to be in *trans* by dideoxy-sequencing). The second patient was diagnosed with sagittal craniosynostosis at five months of age by CT	Green. Multiple cases reported in the literature of homozygous/ compound heterozygous variants.

		scan and was treated with a cranioplasty at six months of age. A homozygous 20 bp deletion in *ADAMTSL4* (c.767_786del, p.(Gln256Profs*38)) was identified in the patient and his sister [102].<br>• A further 12 cases have been previously reported of craniosynostosis and recessive variants in *ADAMTSL4* [102].	
AHDC1	Red	• Whole exome sequencing identified a heterozygous missense variant c.4370A>G; p.(Asp1457Gly) in a child with developmental delay, lack of speech, seizures, structural brain anomalies, craniosynostosis, laryngomalacia and facial dysmorphism [103].<br>• A whole exome and genome sequencing analysis in a cohort of patients with undiagnosed craniosynostosis identified one individual with bicoronal and metopic craniosynostosis and a *de novo* c.2373_2374delTG; p.(Cys791fs*57) variant in *AHDC1* [104].<br>• A patient with bicoronal craniosynostosis was found by whole exome sequencing to harbour a heterozygous variant in *AHDC1* (c.2473C>T; p.(Gln825*)) [105].<br>• Two individuals with Xia-Gibbs syndrome were reported with d*e novo* variants in *AHDC1:* c.3185_3186del; p.(Thr1062Serfs*63) and c.2772del; p.(Arg925Glufs*7) [83].<br>• Two variants were detected in independent families (one with dolichocephaly and the other with brachycephaly, although it is not clear if craniosynostosis was radiologically confirmed) encoding frameshifting variants: c.1206delA; p.(Arg403Alafs*49) and c.1758delA; p.(Lys586Asnfs*37) [106].	Green. More than three cases reported in the literature, although unclear if some of these have radiologically confirmed craniosynostosis.
ALX1	Red	• Analysis of the 100kGP craniosynostosis cohort identified a d*e novo* variant in *ALX1:* c.541C>A; p.(Gln181Lys) [82].	Red. No further evidence.
ALX3	Red	• No further evidence.	Red.
ATR	Red	• An RNA-sequencing study identified several heterozygous rare variants in *ATR* [75]:<br>o c.347G>C; p.(Cys116Ser) – 2 alleles in gnomAD v.2.1.1, no homozygotes, paternally inherited.<br>o c.326G>A; p.(Arg109Gln) – 3 alleles in gnomAD v.2.1.1, no homozygotes.<br>o c.5427A>T; p.(Leu1809Phe) – 16 heterozygotes but no homozygotes in gnomAD v.2.1.1.<br>o c.5476G>A; p.(Val1826Met) – paternally inherited, absent from gnomAD v.2.1.1.<br>o c.7375C>T; p.(Arg2459Cys) – paternally inherited, 2 alleles in gnomAD v.2.1.1.	Red. Reported variants likely heterozygous and *ATR* is an autosomal recessive disease gene.

AXIN2	Red	• A heterozygous variant in *AXIN2* was identified in a pair of twins and their father: c.1181G>A; p.(Arg394His). The variant has an allele frequency of 7.69e-4 – too common to be considered likely pathogenic [107].<br>• A child presenting with sagittal craniosynostosis was found to harbour a *de novo* loss-of-function variant in exon 4 of *AXIN2* (c.1045_1046delCT; p.(Leu349fs*24)), which is absent from gnomAD. Alongside craniosynostosis, the patient displayed frontal bossing, high anterior hair line, depressed nasal bridge, bilateral epicanthus, and low set ears [108].<br>• Animal models are available showing craniosynostosis [109].	Amber. Supporting animal model and an identified loss-of-function variant. Further cases are required to promote PanelApp status.
BBS9	Red	• A patient with sagittal synostosis was described with a variant in *BBS9*: (c.1663C>T; p.(Arg587Gln)). The variant allele frequency is too high to be considered causal (0.0001512 – gnomAD v2.1.1) [110].<br>• A novel c.2209C>G; p.(Leu737Val) variant was described in an individual with non-syndromic right coronal craniosynostosis which was paternally inherited [111].<br>• An association was made with non-syndromic sagittal craniosynostosis and a 120-kb region downstream of *BMP2* flanked by rs1884302 and rs6140226 and within a 167-kb region of *BBS9* between rs10262453 and rs17724206 [112].	Red. Currently only GWAS to support. Other variants are too common or inherited.
BMP4	Red	• A 21-month-old girl was reported with an interstitial deletion of the long arm of chromosome 14, del(14)(q22.1q23.2). She presented with bilateral anophthalmia, absent left external auditory canal, facial asymmetry, micro-retrognathia, hypotonia, psychomotor retardation and lambdoid craniosynostosis, a very small sella turcica and cervical vertebral anomalies [113].	Red. Animal models show its involvement in craniofacial development. One deletion reported which includes multiple genes.
CCBE1	Red	• No further evidence.	Red.
CD96	Red	• No further evidence.	Red.
CEP120	Red	• Ciliopathy gene – no further evidence.	Red.
CHST3	Red	• One report of a case with sagittal craniosynostosis [114].	Red. More cases needed.

COLEC10	Red	• A novel homozygous frameshift deletion variant (c.128_129delCA; p.(Thr43AsnfsTer9)) was identified within the *COLEC10* gene in a 7-year-old affected girl with craniosynostosis, dolichocephaly, blepharoptosis, clinodactyly of the 5th finger, high myopia, long face, micrognathia, patent ductus arteriosus, downslanted palpebral fissures, telecanthus, and epicanthus inversus [115].<br>• Four patients with 3MC syndrome have been described with variants in *COLEC10* but the presence of craniosynostosis is not clear [116].	Red. Only the Iranian case where craniosynostosis is confirmed.
CRTAP	Red	• A child with Cole-Carpenter syndrome was described with a homozygous c.118G>T; p.(Glu40*) variant in exon 1 of the *CRTAP* gene. She was born to consanguineous parents and went on to develop mild thoraco-lumbar scoliosis and sutural craniosynostosis (coronal and lambdoid synostosis) [117].	Red. Further cases required.
DHRS3	Red	• Unpublished cases are already reported on PanelApp.	Red. No further cases to add to the evidence already reported on PanelApp.
EDNRB	Red	• No further evidence.	Red.
EFNA4	Red	• Three out of 81 patients with non-syndromic coronal synostosis were described but the variants were all too common to be considered pathogenic [118].<br>• A heterozygous variant was described in the Australia/New Zealand craniosynostosis cohort: c.211G>A; p.(Glu71Lys). This variant has an allele frequency of 2.79e-5 in v2.1.1 (7 alleles/ ~250,000) [74].<br>• A female with unilateral, postaxial polydactyly, and bilateral fifth fingernail duplication was found on next-generation sequencing to harbour a novel, likely pathogenic, variant in intron 3 of the *TBX3* gene (c.804+1G>A; p.(?)). This variant was inherited from the proband's affected father. However, the father had an additional clinical finding of congenital sagittal craniosynostosis and whole genome sequencing analysis detected a variant in the *EFNA4* gene (c.178C>T; p.(His60Tyr)). This variant has an allele frequency of 1.29e-3 which is too high for causal contribution [119].<br>• A further variant was identified in the RNA-sequencing cohort of patients with single suture craniosynostosis: c.550C>T; p.(Leu184Phe) [75].	Red. Reported variants are all in gnomAD at relatively high frequency – only exception is p.(Lys95Glu).

		• Three missense variants (total of 101 individuals in the screen) were identified in the *EFNA4* gene in children with metopic and sagittal synostosis: c.178C>T; p.(His60Tyr), c.283A>G; p.(Lys95Glu) (absent from gnomAD v2.1.1), c.349C>A; p.(Pro117Thr) (337 alleles in gnomAD)). All variants were present in at least one non-affected family member. One child with metopic synostosis expressed concomitant variants in *ALX4*: c.304C>T; p.(Pro102Ser) and c.104G>C; (p.Arg35Thr), and *EFNA4*: c.283A>G; p.(Lys95Glu)) [120].	
FBN1	Red	• Two variants reported in the Clarke paper but both detailed in gnomAD v2.1.1: c.1169C>T; p.(Ser390Phe) with an allele frequency of 2.40e-5, and c.8149G>A; p.(Glu2717Lys) with an allele frequency of 1.35e-4 [75].<br>• Two recurrent novel heterozygous *FBN1* mutations were found in two patients with Marfan syndrome (MFS) and abnormal cranial dura: a *de novo* c.3302G>A; p.(Tyr1101Cys) and a *de novo* c.3217G > A; p.(Glu1073Lys) variant [23].<br>• A *de novo* c.8226+5G>A; p.(?) splicing variant was identified in one individual with sagittal and metopic synostosis [104].<br>• Mutation analysis identified a c.8175_8182del8bp; p.(Arg2726Glufs*9) variant in exon 64 of the *FBN1* gene (pLI score of 1) in an individual with overgrowth and craniosynostosis [96].<br>• Interstitial deletions involving the chromosomal band 15q15 are described in five prior cases and a further case was identified by Hiraki and colleagues in an individual with coronal, metopic and sagittal synostosis, resulting in the deletion of *FBN1* [121].<br>• A further patient with Marfan syndrome and a familial variant in *FBN1* was identified: c.4096G>A; p.(Glu1366Lys) (not reported in gnomAD) [79].	Green. Five likely pathogenic *de novo* variants reported in independent families and one deletion including *FBN1*.
FGF3	Red	• A duplication involving bands 11q11 and 11q12 was reported in a patient with multi-suture craniosynostosis, congenital heart defects and developmental delay. Their mother is a carrier of a mosaic duplication: 46,XY,dup(11)(q11-->q13.3)(29)/46,XY(6). An overlapping region of less than 1.2 Mb was identified and included the duplication of genes *FGF3* and *FGF4* in both individuals [122].	Red. More cases required.
FGF4	Red	• Duplication identified involving *FGF3* and *FGF4* (as detailed above) [122].	Red. More cases required.

FGF9	Red	• *Fgf9* elbow-knee-synostosis mouse model develops craniosynostosis and features of multiple synostoses syndrome [123].<br>• A female was identified with a heterozygous *FGF9* variant: c.427A>T; p.(Asn143Tyr). This variant affects the same amino acid as the well characterized spontaneous Eks mouse variant [124].<br>• The first *FGF9* variant, c.296G>A; p.(Ser99Asn), was reported in a large multigenerational Chinese family (12 patients) with multiple synostoses syndrome 3. Functional studies show that p.(Ser99Asn) compromises chondrocyte proliferation and differentiation, leading to increased osteogenic differentiation and matrix mineralisation of bone marrow-derived mesenchymal stem cells [125].<br>• A father and son with multiple synostoses syndrome and craniosynostosis (the father displayed coronal synostosis, while his son showed premature closure of the sagittal suture) were reported to harbour a heterozygous variant in *FGF9*: c.184A>G; p.(Arg62Gly) [36].	Green. Variants reported in three independent families and a mouse model
FLNB	Red	• No further evidence.	Red.
FREM1	Red	• In the Norwegian cohort two 9p deletions were identified in patients with craniosynostosis, developmental delay, and reduced vision: g. 204193_ 18073357del (17.8Mb) and g.13638428_ 17121764del (3.5Mb) [83].<br>• Two out of 28 patients with craniosynostosis in the Saudi Arabia cohort harboured *FREM1* variants: (1) a heterozygous *de novo* variant in an individual with multi-suture synostosis: c.916_936dup; p.(Glu306_Leu312dup), (2) and two variants in *cis* in a family with metopic synostosis: c.4023C>G; p.(Cys1341Trp), and c.4564G>A; p.(Val1522Met) [81].<br>• One report showed no association with heterozygous *FREM1* deletions and trigonocephaly in an independent cohort [126].	Amber. Two additional copy number variants to add to the original paper describing five patients with metopic synostosis and *FREM1* deletions, although both large deletions encompass multiple genes [127].
GINS2	Red	• Exome sequencing was performed to investigate the genotype of an individual presenting with prenatal and postnatal growth restriction, a craniofacial gestalt of Meier-Gorlin syndrome and coronal craniosynostosis. A novel homozygous missense variant, c.341G>T; p.(Arg114Leu), in *GINS2* was identified – both non-consanguineous healthy parents carried this variant [128].	Red. Single case study with additional functional work; further cases are required to promote status.
GPC3	Red	• A patient with Simpson-Golabi-Behmel syndrome, presenting with left coronal craniosynostosis, penoscrotal hypospadias, and a large prostatic utricle was found to	Amber. Further cases required.

		harbour a frameshifting variant which was *de novo* in their mother. Additionally, the patient was shown to have a *de novo* 6p24.3p24.2 duplication and a paternally inherited 15q26.1 duplication detected by chromosomal microarray. The *de novo* duplication on chromosome 6 includes *TFAP2*, which is responsible for branchio-oculo-facial syndrome when deleted or mutated [129].<br>• One report of a patient with a variant in *GPC3* and metopic synostosis [130].<br>• A deletion including *GPC3* was identified in the 100kGP cohort of patients with craniosynostosis [82].	
IFT140	Red	• One male patient with distinctive clinical features of Sensenbrenner syndrome (dolichocephaly, shortening of long bones and early onset renal failure, ectodermal anomalies and small teeth was found to harbour a tandem duplication variant p.(Tyr1152_Thr1394dup) on one allele and a novel missense variant, p.(Leu109Pro), on the second allele [131].<br>• An individual with trigonocephaly and additional ciliopathy-related clinical features was found to harbour compound heterozygous variants in the *IFT140* gene: a substitution at the splice donor site of exon 24 (c.723+1G>T; p.(?)) and a 17 bp deletion, impacting the first coding exon (c.-11_6del; p.(?)). These variants were confirmed by dideoxy-sequencing [132].<br>• A patient was reported with brachydactyly (it is not clear if craniosynostosis was radiologically confirmed) and Sensenbrenner syndrome. Whole exome sequencing as part of the DDD study evidenced compound heterozygosity for *IFT140*: c.634G>A; p.(Gly212Arg) and c.2278C>T; p.(Arg760Ter) [133].	Amber. Three patients with compound heterozygous/ homozygous variants, but it is not clear if these were radiologically confirmed.
IFT43	Red	• A homozygous c.1A>G; p.(Met1Val) variant affecting the *IFT43* gene was identified in an individual with anaemia and renal dysfunction, short stature, short limbs, brachydactylia, tooth agenesis, and retinal dystrophy, high-degree myopia, and chronic renal failure; it is not clear if this patient has craniosynostosis [134].<br>• Sequence analysis of candidate genes *TTC8* and *IFT43* in two affected siblings with Sensenbrenner syndrome revealed a homozygous variant in the translation initiation codon in exon 1 of *IFT43* (c.1A>G; p.(?)); only the younger sibling displayed craniosynostosis [135].	Amber. Upgrade as per comments already detailed on PanelApp.
IGF1R	Red	• Compound heterozygous variants were identified in an individual with delay in speech acquisition, hyperkinesia, and trigonocephaly: c.914A>T; p.(Gln305Leu) and c.3583G>A; p.(Val1195Ile) [78].	Red. Heterozygous variants are too common to be likely pathogenic.

		• In the Clarke cohort, two heterozygous variants were identified: c.3737G>A; p.(Arg1246His) with 7 alleles in gnomAD v.2.1.1, and c.4058G>A; p.(Arg1353His) with 70 alleles in gnomAD (out of ~250,000) v.2.1.1 [75].<br>• Resequencing of the coding regions, splice junction sites, and 5' and 3' untranslated regions of 27 candidate genes in 186 cases of isolated non-syndromic single suture synostosis revealed five sequence variants in *IGF1R* [136]:<br>o p.(Arg406His): 10/~280,000 alleles, gnomAD v.2.1.1<br>o p.(Arg595His): 178/~280,000 alleles, gnomAD v.2.1.1<br>o p.(Asn857Ser): 579/~280,000 alleles, gnomAD v.2.1.1<br>o p.(Pro190Ser): 215/~280,000 alleles, gnomAD v.2.1.1<br>o p.(Met446Val): 310/~280,000 alleles, gnomAD v.2.1.1<br>• An array comparative genomic hybridization revealed a 4.71 Mb duplication from 15q26.2 to 15q26.3 encompassing the *IGF1R* gene. The pregnancy was subsequently terminated, and a 1062-g (>99(th) centile) malformed foetus was delivered at 24 weeks of gestation with craniofacial dysmorphism, craniosynostosis, and overgrowth [84].	
IMPAD1	Red	• No further evidence to add compared to what is already detailed in PanelApp.	Red.
IRX5	Red	• A heterozygous missense variant was identified in the Clarke cohort: c.1025C>T; p.(Thr342Ile) [75].	Amber. Further cases reported in PanelApp, but one without confirmed craniosynostosis.
KANSL1	Red	• An additional 10 patients with *KANSL1* haploinsufficiency syndrome were screened – 9 of these individuals had a 17q21.31 deletion and one harboured a *de novo* intragenic variant in *KANSL1* (c.1652+2T>C; p.(Leu552Phefs*14)). One patient with a chromosome deletion in this cohort had sagittal craniosynostosis. Overall, craniosynostosis affected two out of 42 patients in this cohort (5%; including the paper published in 2015), both in association with 17q21.31 deletions [137, 138].	Amber. Two cases of craniosynostosis in Zollino report associated with 17q21.21 deletions (which includes *KANSL1*).
KAT6B	Red	• A *de novo* variant was identified in a study of Norwegian patients with craniosynostosis: c.3769_3772del; p.(Lys1258Glyfs*13) [83].<br>• Exome sequencing identified a *de novo* variant in *KAT6B*, c.4572dupT; p.(Thr1525Tyrfs*16) in a patient with a phenotype suggestive of Lin-Gettig syndrome and sagittal synostosis. A second patient with sagittal synostosis was also found to harbour a *de novo* variant in *KAT6B*: c.4205_4206delCT; p.(Ser1402Cysfs*5) [139].	Green. Three described patients with *de novo* loss of function variants. Craniosynostosis is a less frequently associated phenotype of Lin-Gettig syndrome.

KDM6A	Red	• No further evidence.	Red.
LMX1B	Red	• No further evidence. One known case (p.Leu203Pro) that is unpublished.	Red.
LRP5	Red	• One heterozygous variant reported in the Clarke cohort: c.3404G>A; p.(Arg1135His). This variant is listed in ClinVar as likely benign and there are 102/~250,000 alleles in gnomAD [75].<br>• An additional heterozygous variant was reported within the Clarke cohort: c.4423C>T; p.(Arg1475Trp) (5 alleles in gnomAD v.2.1.1). However, this individual also harbours a potentially damaging *FAM20C* variant (p.Ser410Thr) [75].<br>• An extended four-generation family with 13 affected individuals (7 men and 6 women) in which an autosomal dominant type of osteosclerosis segregated were found to have a missense variant in LRP5: c.640G>A; p.(Ala214Thr). Craniosynostosis was reported in four affected family members (two males and two females) [140].	Red. Additional reports of variants in *LRP5* are either too common in gnomAD, or not the sole variant identified.
NFIX	Red	• In a patient with lambdoid and bicoronal craniosynostosis, a *de novo* variant encoding p.(Arg121Cys) was identified in *NFIX* [86].<br>• A child with metopic craniosynostosis was shown to harbour a variant within the DNA-binding domain of *NFIX* (p.(Arg116Trp)) [86].<br>• A *de novo* microdeletion within *NFIX* ((c.(818+1_819-1)_(1078+1_1079-1)), deleting exons 6–7 and resulting in a predicted p.(Ser273Argfs*63) frameshift variant, was identified via microarray in a child with syndromic sagittal and coronal craniosynostosis [86].<br>• A child with syndromic sagittal craniosynostosis was shown to have a *de novo* missense variant within the nuclear localisation sequence of *NFIX* (p.(Met48Lys)) [83].	Green. Four families with variants in *NFIX* – all absent from gnomAD. Only variant not affecting a functional domain is p.(Met48Lys) which is reported in ClinVar as likely pathogenic for Malan overgrowth syndrome.
NOG	Red	• No further evidence.	Red.
OSTM1	Red	• No further evidence. One case listed on PanelApp.	Red.
PAX3	Red	• No further evidence.	Red.
PTPRD	Red	• Microarray chromosomal analysis revealed the presence of a homozygous deletion involving the *PTPRD* gene, located on chromosome 9p22.3. Reverse transcriptase PCR (RT-PCR) along the length of the gene failed to amplify the patient's cDNA in fibroblasts, indicating the presence of two null *PTPRD* alleles [141].	Red. No further evidence in addition to what is already detailed in PanelApp.
RSPRY1	Red	• Whole exome sequencing revealed a novel homozygous, c.377delT; p.(Ile126fs*), frameshift variant in exon 2 in one family. Dideoxy-sequencing revealed a homozygous	Amber. Three families identified with variants in *RSPRY1* and

		splice site variant c.516+2T>A; p.(?) in a second family. In family 1, all but one of the 4 individuals with the variant were reported with craniosynostosis [142].<br>• Segregation of a 1 bp homozygous duplication in *RSPRY1* was identified in a family with four affected individuals with craniosynostosis: c.1279dupA; p.(Thr427Asnfs*10) [143].	craniosynostosis, however the phenotype is not fully penetrant in one family of the Simsek-Kiper paper.
SCARF2	Red	• An RNA-sequencing analysis on a cohort of patients with single suture craniosynostosis identified four variants in *SCARF2* [75]:<br>o c.1638G>T; p.(Trp546Cys): no parental samples available, allele frequency = 4.60e-5 gnomAD v.2.1.1<br>o c.1688T>G; p.(Val563Gly): *de novo* and absent from gnomAD<br>o c.1999A>C; p.(Lys667Gln): maternally inherited, absent from gnomAD<br>o c.2593dupG; p.(Ala865Serfs*184): paternally inherited, absent from gnomAD<br>• A homozygous variant was reported as pathogenic for Van den Ende-Gupta syndrome: c.190T>C; p.(Cys64Arg) [144].	Amber. The significance of the heterozygous variants is not clear as only one is *de novo*. Further cases required.
SCN4A	Red	• Two brothers with lower facial weakness, highly arched palate, scaphocephaly due to synostosis of the sagittal and metopic sutures, axial hypotonia, proximal muscle weakness, and mild scoliosis were found to be compound heterozygous for c.3425G>A; p.(Arg1142Gln) and c.1123T>C; p.(Cys375Arg) variants in *SCN4A* on exome sequencing. Functional analysis showed that the p.(Cys375Arg) variant conferred complete loss of function and p.(Arg1142Gln) caused partial loss-of-function of the $Na_v1.4$ channel [145].	Red. No further evidence.
SH3PXD2B	Red	• Two heterozygous variants were identified in the Clarke cohort: c.1265T>C; p.(Ile433Thr) and c.2276C>G; p.(Pro759Arg) [75].<br>• Three siblings were shown to harbour a novel homozygous variant caused by the deletion of exon 13 of the *SH3PXD2B* gene; two of the three siblings have non-scaphocephalic sagittal synostosis associated with raised intracranial pressure [146].	Red. Only one family with a novel homozygous variant; further cases required.
SLC3A2	Red	• No further evidence.	Red.
SOX10	Red	• No further evidence.	Red.
TCOF1	Red	• A patient was reported with a novel *de novo* nonsense variant c.2731C>T, encoding p.(Arg911*) and classical features of Treacher Collins syndrome; they were additionally documented with craniosynostosis, choanal atresia, and oesophageal regurgitation [147].<br>• Targeted gene panel sequencing in a cohort of sixteen patients from 11 consecutive families with Treacher Collins Syndrome Type 1 showed the presence of three novel	Red. Further cases required.

		pathogenic variants in the *TCOF1* gene – c.2145_2148dupAAAG; p.(Ser717Lysfs*42), c.4370delA; p.(Lys1457Argfs*118), and c.83G>C; p.(Arg28Pro) (a polymorphism). It is not clear that all patients had craniosynostosis [148].	
TICRR	Red	• No further evidence. One unpublished case reported on PanelApp.	Red.
TWIST2	Red	• No further evidence.	Red.
WDR19	Red	• Two infants with Sensenbrenner syndrome were reported with variants in *WDR19*. Genetic testing identified compound heterozygous variants in *WDR19* in both patients (Patient 1: c.953delA, c.3533G>A, Patient 2: c.2645+1G>T, c.3533G>A). The report notes dolichocephaly, but it is unclear if craniosynostosis was radiologically confirmed [149].<br>• Scaphocephaly, hyperlaxity, severe growth retardation (height under −3 SDS and weight under the 1st centile), nail dysplasia, and psychomotor delay were confirmed in an individual with compound heterozygous variants in *WDR19*: c.373_375dup; p.(Asn125dup), inherited maternally, and c.2269C>T; p.(Gln757*), inherited paternally [150].	Red. One confirmed case, one family with dolichocephaly but not radiologically confirmed. More cases required.

Table S4 Genes not currently listed in PanelApp but with one or more variants described in a patient with craniosynostosis.

Gene	Literature	Suggested PanelApp Status
ABBC9	• An individual was described with Cantu syndrome (autosomal dominant overgrowth syndrome with facial dysmorphism, congenital hypertrichosis, and cardiomegaly) and bicoronal synostosis. His father also had Cantu syndrome but did not display craniosynostosis [151].	Red. Further cases required.
ACVRL1	• A *de novo* variant was identified in a cohort of patients with lambdoid synostosis (encoding, p.(Val228Ile)); ExAC frequency reported 1.65 x 10^{-5} [86].	Red. Further cases required.
ACVR2A	• A *de novo* variant was identified in a cohort of patients with lambdoid synostosis (encoding, p.(Thr63Ala)) [86].	Red. Further cases required.
ANKH	• One patient described in the Chinese cohort with syndromic bicoronal and sagittal synostosis: c.1129_1132delinsC; p.(Phe377del). This variant is reported as pathogenic for craniometaphyseal dysplasia in ClinVar and is absent from gnomAD v.2.1.1 [84].	Red. Further cases required.
ARAP3	• An individual with metopic synostosis was described with a *de novo* variant in *ARAP3*: IVS6+1delGT; p.(?) [152].	Red. Further cases required.
ARID1B	• Analysis of the UK 100kGP identified a frameshift variant in *ARID1B* in a patient with intellectual disability and sagittal synostosis: c.3594delinsCCCCCA; p.(Gly1199Profs*14) [82].<br>• A further frameshifting variant was described in an individual within the Chinese cohort with sagittal craniosynostosis c.2346_2352del; p.(Ser784Cysfs*59) [84].<br>• An additional patient was described with trigonocephaly and motor developmental delay with a variant in *ARID1B*: c.2277delC; p.(Pro760fs) [78].<br>• A *de novo* variant affecting *ARID1B* (c.1468_1472delTGGGC; p.(Trp490Glyfs*43)) was identified in an individual with craniosynostosis out of a cohort of neurodevelopmental disorder patients [14].	Green. Four independent families with loss-of-function variants in *ARID1B* and craniosynostosis.

ASXL3	• A *de novo* c.3033dup; p.(Leu1012Serfs*23) was identified in a patient with metopic synostosis within the Norwegian cohort [83].<br>• A six-year-old with microcephaly, autism, global developmental delay, and metopic craniosynostosis was found on exome sequencing to harbour a heterozygous two base pair *de novo* deletion, c.1897_1898delCA; p.(Gln633Valfs*13) in *ASXL3 [153].*<br>• A heterozygous *de novo* single nucleotide variant (c.3039+1G>A; p.(?)) in the invariant "GT" splice donor site of exon 11 was identified in an individual with a prominent forehead, thick eyebrows, long lashes, exotropia, depressed nasal ridge, thin upper lip vermillion, hirsutism, microcephaly, bilateral camptodactyly of third, fourth and fifth fingers, deep palmar creases, and small hands and feet. Craniosynostosis is not confirmed [154].	Amber. Two cases of loss-of-function variants in *ASXL3;* only one has radiologically confirmed craniosynostosis.
AXIN1	• One individual was described with sagittal synostosis and a *de novo* heterozygous variant in *AXIN1*, encoding p.(Glu322Gly) [152].	Red. Further cases required.
BCL11B	• Mosaic frameshift from the 100kGP data was identified [not published] – c.781_808dup, encoding a 28 bp insertion.<br>• Three other cases are known [unpublished]: c.2438_2459del; (p.Val813Alafsı24); 14qdel GRch37 arr 14q32.2(96,723,247-100,938,073)x1; Deletion arr[hg19]14q32.2(99,052,763-100,591,634)x1.<br>• A *de novo* substitution was described in *BCL11B* (c.7C>A; p.(Arg3Ser)) and functionally characterised in a mouse model. The variant caused loss of interaction with transcriptional complexes and craniosynostosis [18].<br>• A patient was described with a *de novo* frameshift variant in *BCL11B*, identified by whole-exome sequencing: c.2346_2361del; p.(Gly783Alafs*24) [155].<br>• An additional *de novo* loss of function variant has been described in a patient with developmental delay and craniosynostosis: c.2439_2452dup; p.(His818Argfs*31) [156].	Green. 7 families with variants in *BCL11B* and confirmed craniosynostosis.
BRWD3	• A *de novo* stop-gain was identified in an individual with craniosynostosis within the 100kGP cohort: c.4012C>T; p.(Gln1338*) [82].	Red. Further cases required.
CACNA1E	• A heterozygous splice variant was identified in a cohort of patients with trigonocephaly: c.3674+5A>G; p.(?) [78].	Red. Further cases required.
CDK8	• Metopic synostosis was described in one individual out of 12 with variants in *CDK8*. The individual harboured a *de novo* c.88G>A; p.(Gly30Ser) variant [157].	Red. Further cases required.
CDK13	• A *de novo* missense variant was identified within the UK 100kGP cohort of patients with craniosynostosis: c.2563G>C; p.(Asp855His) [82].	Green. Four independent cases identified.

	• A further *de novo* variant was identified in an individual within the Norwegian cohort: c.2524A>G; p.(Asn842Asp) [83].<br>• Two patients were described with craniosynostosis in a cohort of patients with congenital heart defects, dysmorphic facial features, and intellectual disability [19].	
CHD3	• One individual identified with Snijders Blok Campeau syndrome (neurodevelopmental disorder, macrocephaly and impaired speech and language) to have sagittal synostosis: c.3482A>G; p.(His1161Arg) [158].	Red. Further cases required.
COL11A1	• A *de novo* loss of function variant (c.2852+5G>A; p.(?)) was identified in an individual with craniosynostosis and a hearing impairment from the UK 100kGP [82].	Red. Further cases required.
CTNNA1	• A de novo insertion was identified in a screen of patients with syndromic craniosynostosis: encoding, p.(Val374_375ins) [77].	Red. Further cases required.
DDX3X	• A novel *de novo* missense mutation was identified in the *DDX3X* gene (c.625C>G) by whole exome sequencing in a child with craniofacial dysmorphisms: brachycephaly and a flattened triangular-asymmetrical face characterized by micrognathia, mild hypertelorism, wide and prominent nose, short philtrum, thin lips and macroglossia [159].<br>• Exome-sequencing identified three distinct *de novo* heterozygous variants in *DDX3X*: c.1511G>A; p.(Gly504Glu) (Patient 1), c.1436_1439delinsTCTC; p.(Asp479Arg480delinsValSer) (Patient 2), and c.641_643delTCA; p.(Ile214del) (Patient 3). The patients showed severe intellectual disability/developmental disorders, microcephaly, and dysmorphic features. Plagiocephaly was observed in Patient 1 and Patient 2 was diagnosed with sensorineural hearing loss and trigonocephaly. However, craniosynostosis was only radiologically confirmed in Patient 2 [160].<br>• A *de novo* variant in *DDX3X* was identified in an additional patient with trigonocephaly, delay in speech acquisition and motor developmental delay: c.1616-2A>G; p.(?) [78].	Amber. Five cases reported with variants in *DDX3X* and likely craniosynostosis – only radiologically confirmed in two cases. Gene identified in DDD study [161].
DPF2	• Variants in *DPF2* are associated with Coffin Siris syndrome. Two patients with sagittal synostosis were found to harbour a *de novo* c.1037A>G; p.(Asp346Gly) variant. A third patient with suspected metopic synostosis, owing to trigonocephaly, was identified with a c.1099+1G>A; p.(Asp340Glufs*12) frameshifting variant. All patients displayed phenotypic features of Coffin Siris syndrome [162].	Amber. Further cases required as radiographic images were not obtained for the individual with trigonocephaly.
DPH1	• A patient was described with short stature, sagittal craniosynostosis and dysmorphic features including scaphocephaly, sparse hair, multiple dental anomalies, epicanthal folds	Amber. Further cases required as no functional data.

	and hypoplastic toenails to harbour a homozygous missense variant in *DPH1*: c.17T>A; p.(Met6Lys) (born to consanguineous parents).<br>• A family with two affected siblings were described and one was confirmed to have metopic synostosis. They harboured a recessive c.335A>G; p.(Tyr112Cys) variant [163].	
DVL3	• An individual was described with sagittal synostosis and a *de novo* variant in *DVL3*: encoding, p.(Gly327fs*) [152].	Red. Further cases required.
EHMT1	• A de novo splicing variant was identified in *EHMT1* within the Norwegian cohort: c.2018+1G>C; p.(?) [83].	Red. Further cases required.
EIF5A	• An intronic variant was identified in *EIF5A* within the Chinese cohort in a patient with metopic synostosis: c.271-1G>C; p.(?) [84].	Red. Further cases required.
EXTL3	• A recessive variant in *EXTL3* was identified in a patient with metopic craniosynostosis, intellectual disability, short stature, microcephaly, hip dysplasia, kyphosis, delayed skeletal age and immunodeficiency: c.2392G>A; p.(Val798Met). The variant is absent from gnomAD and affects the glycosyl transferase family 64 domain [85].	Red. Further cases required.
FBXO11	• A *de novo* insertion was identified within the 100kGP cohort of patients with craniosynostosis: c.2731_2732insGACA; p.(Thr911Argfs*5) [82].<br>• Two patients were described with craniosynostosis and variants in *FBXO11*: c.2518T>C, p.(Ser840Pro) in an individual with sagittal synostosis, and hg19: chr2: g.48060020C>G, c.1042-1G>C; p.(?) [30].	Green. Three independent cases identified.
FOXP1	• Whole exome sequencing identified a *de novo* splicing mutation in *FOXP1* in a patient with syndromic intellectual disability and trigonocephaly. The variant (c.1428+1 G>A) results in skipping of exon 16, a frameshift and a premature stop codon (p.(Ala450GLyfs*13)) [164].	Red. Further cases required.
FOXP2	• A familial variant in *FOXP2* was identified in individuals with developmental delay, hypermetropia, orofacial dyspraxia and sagittal craniosynostosis: c.484del; p.(Gln162fs). The variant is absent from gnomAD [85].	Red. Variable presentation of craniosynostosis. Variants in this gene are normally associated with language disorders.
FOXO1	• A de *novo* novel variant was identified within the Timberlake cohort of patients with lambdoid synostosis, encoding p.(Arg180Trp) [86].	Red. Further cases required.

FTO	• A homozygous variant in *FTO* (c.956G>A; p.(Arg322Gln)) was described in one individual with multiple malformation syndrome, which included craniosynostosis. Craniosynostosis is not a consistent feature of variants in *FTO* [165].	Red. Further cases required.
FUZ	• A pair of monozygotic twins were described with craniosynostosis and a novel variant in *FUZ* (c.851G>C; p.(Arg284Pro)) [166].	Red. Further cases required.
GLI2	• A *de novo GLI2* variant was identified in an individual with syndromic craniosynostosis, encoding p.(Ala551Thr) [77].	Red. Further cases required.
GPC4	• A variant in *GPC4* was identified in an individual with syndromic craniosynostosis; the variant, encoding p.(Val152fs), arose *de novo* in the mother [77].	Red. Further cases required.
GLIS3	• One patient was described with a variant in *GLIS3* and sagittal craniosynostosis requiring surgical intervention. The patient harboured a homozygous deletion of exons 9 – 11; consanguinity was not confirmed but suspected [167].	Red. Further cases required.
H3F3A/B	• In a cohort of 33 patients with *H3F3A* variants and 13 patients with *H3F3B* variants, one study identified 14/46 (30%) individuals with "craniosynostosis or abnormal head shape". It is not clear if these were radiologically confirmed and what proportion of this subset of patients had synostosis compared to dysmorphic features [168].	Red. Further cases required.
HDAC4	• A patient was described with two copy number variants: g.233110452_ 243028452 and del g.210300_ 8664358dup. The second variant was maternally inherited [83].	Red. Further cases required.
HIST1H1E	• A patient with syndromic unilambdoid synostosis was found to harbour a frameshifting variant in *HIST1H1E*: c.433_434insC; p.(Thr146Hisfs*50). The variant is reported as pathogenic for Rahman syndrome in ClinVar and absent from gnomAD [84].	Red. Further cases required.
IFRD1	• A *de novo* variant was identified in a cohort of patients with lambdoid synostosis (p.(Gly6fs*)) [86].	Red. Further cases required.
IFT140	• One recessive variant was identified in the 100kGP cohort: c.21011G>C; p.(Ala701Pro) [82].	Red. Further cases required.
IL6ST	• A homozygous non-synonymous variant in *IL6ST* (p.(Arg281Gln)) was described in a patient with craniosynostosis and retained deciduous teeth. Findings were supported using a mouse model with the missense variant which resulted in lower litter sizes, facial synostosis, and teeth abnormalities. The model phenocopies aspects of *IL11RA* deficiency in humans and mice [169].	Green. Two cases of recessive variants in *IL6ST* and craniosynostosis and an animal model which phenocopies the human.

	• A patient with a homozygous variant in *IL6ST* presented with recurrent infections, eczema, bronchiectasis, high IgE, eosinophilia, defective B cell memory, and an impaired acute-phase response, as well as skeletal abnormalities including craniosynostosis. They were shown to harbour a p.(Asn404Tyr) missense substitution [42].	
KMT5B	• A *de novo* loss of function variant was identified within the 100kGP craniosynostosis cohort: c.557T>A; p.(Leu186*) [82].	Red. Further cases required.
KPTN	• Four families were described with variants in *KPTN* and suspected craniosynostosis. Sagittal synostosis was confirmed in one individual from a family with 3 affected individuals. All families harboured a variant encoding p.(Ser259*); this was homozygous in four individuals and in *trans* with another heterozygous variant (p.(Met241_Gln246dup)) in 5 individuals [170].	Amber. Further cases required with confirmed craniosynostosis.
MACF1	• An individual was described with sagittal synostosis and a novel splicing variant in *MACF1*: IVS89+1G>A; p.(?) [152].	Red. Further cases required.
MAN2B1	• A Norwegian study of patients with craniosynostosis identified a homozygous missense variant in *MAN2B1*: c.1055T>C; p.(Leu352Pro) [83].<br>• Compound heterozygous variant were identified through screening 114 families with craniosynostosis within the UK 100kGP: c.1830+1G>C; p.(?) and c.2248C>T; p.(Arg750Trp) [82].<br>• One patient out of 12 with recessive variants in *MAN2B1* was described with craniosynostosis: c.2245C>T; p.(Arg749Trp), and c.2355G>A; p.(Thr785*) [49].	Green. Three independent families with recessive variants in *MAN2B1* and craniosynostosis. However, not all individuals with recessive variants in *MAN2B1* develop CRS.
MED13L	• Two siblings exhibited an intragenic deletion of exons 3-14 resulting in *MED13L* haploinsufficiency syndrome (intellectual disability, developmental delay, heart defects and dysmorphic features). The deletion was inherited from their mother who showed low frequency mosaicism. The older sibling also presented with craniosynostosis [171].	Red. Further cases required.
MMP21	• Compound heterozygous variants were identified in an individual within the 100kGP with heterotaxy and craniosynostosis: c.671_684del; p.(Val224Glyfs*29) and c.775C>G; p.(His259Asp) [82].	Red. Further cases required.
NAA25	• One individual was described with sagittal synostosis to harbour a *de novo* frameshifting variant in *NAA25*: p.(Phe359fs*) [104].	Red. Further cases required.

NTRK2	• A heterozygous stop-gain variant was identified in an individual with unicoronal synostosis, language delay, hyperphagic obesity, and aggression (c.1330G>T; p.Gly444*). It was suspected that the variant arose *de novo* but the father's sample was not available for testing [104].	Red. Further cases required.
OGT	• A *de novo* variant in *OGT* was identified within the 100kGP cohort: c.539A>G; p.(Tyr180Cys) [82].<br>• An analysis of a cohort of patients with trigonocephaly identified a splicing variant in *OGT*: c.1947+5A>C. The patient displayed a delay in speech acquisition, hyperkinesia, sleep disorders and trigonocephaly [78].	Amber. Further cases required.
OSTM1	• An individual was reported with osteopetrosis, craniosynostosis, and Chiari malformation type 1 and two novel homozygous variants in *OSTEM1*. The first was a missense variant c.265T>A, encoding p.(Val122Asp), which was considered neutral. The second variant was a synonymous change (c.108C>T; p.(=)) but was predicted to create a new donor splice site and disrupt mRNA processing [172].	Red. Further cases required.
PITX2	• Two cases of *de novo* missense variants are known but not reported [unpublished].	Red. Further cases required.
POLR2A	• A *de novo* insertion was identified in the Norwegian sequencing study: c.4329_4330delinsAA; p.(Ala1444Thr). The variant is absent from gnomAD (v.2.1.1) but is not predicted to affect a functional domain. The patient displayed metopic synostosis, impaired motor skills, hypospadias, hypermobile joints, hyperactive behaviour, and tics [85].	Red. Further cases required.
PSMC2	• An individual was described with metopic synostosis and a *de novo* variant in *PSMC2,* encoding p.(Arg297Gly) [152].	Red. Further cases required.
PSMC5	• An individual was described with metopic synostosis and a *de novo* variant in *PSMC5,* encoding p.(Arg317Trp) [152].	Red. Further cases required.
PSMD12	• A heterozygous variant was identified in the UK 100kGP (parents were not available for testing): c.1284G>A; p.(Trp428*) [82].	Red. Further cases required.
PTH2R	• A boy presenting with sagittal and metopic synostosis was found to harbour a complex paracentric inversion involving 2q14.3 and 2q3. An intronic break of the *PTH2R* gene was detected by whole genome sequencing and fluorescence in situ hybridisation [173].	Red. Further cases required.

PUF60	• A patient presenting with Verheij syndrome, characterised by craniofacial dysmorphism, multiple congenital anomalies and variable neurodevelopmental delay, was identified with a heterozygous variant in the splicing factor *PUF60*: c.436C>T; p.(Arg146Cys). They displayed fusion of the coronal and sagittal suture [174].	Red. Further cases required.
RAF1	• A girl with short stature, obstructive hypertrophic cardiomyopathy, multiple facial lentigines, high and wide forehead, downslanting palpebral fissures, low-set ears, short neck, and pectus excavatum, was shown on sequencing to have a heterozygous c.788T>G; p.(Val263Gly) variant. Although the variant was not detected in the healthy father, the mother's DNA sample was not available for study. To date, only three *RAF1* pathogenic variants have been associated with a similar phenotype: p.(Ser257Leu), p.(Ser259Leu), and p.(Leu613Val), all of them activating substitutions that increase signal flow through the RAS/MAPK pathway [175].	Red. Further cases required, not confirmed if the p.(Val263Gly) variant was *de novo* or inherited.
RASAL2	• An individual was described with sagittal synostosis and a *de novo* variant in *RASAL2,* encoding p.(Arg571Pro) [152].	Red. Further cases required.
SCN8A	• A heterozygous duplication involving *SCN8A* was identified in an individual with hearing impairment, hypermobility, intellectual disability, ventricular septal defect and craniosynostosis (c.3924dup; p.(Arg1309Thrfs*3) [82].	Red. Further cases required.
SH3BP4	• A patient was described with a recessive variant in *SH3BP4* in the Norwegian craniosynostosis cohort: c.128C>A; p.(Pro43His). The variant has a CADD score of 33 and a gnomAD allele frequency of 9.6×10^{-5} (no homozygotes are reported in gnomAD). The patient presented with a Chiari I malformation, exophthalmos, eating difficulties as an infant, microcephaly, recurrent infections, dysmorphic features, Kabuki-like syndrome, and pan-synostosis. This patient also harbours two variants in *KMT2D*: c.11599C>A; p.(Gln3867Lys) (CADD = 22) and c.7182C>A; p.(Ser2394Arg) (CADD = 20); gnomAD frequency 1.2×10^{-5} and absent, respectively [85].	Red. No further supporting evidence.
SIX2	• A family with a dominantly inherited craniofacial phenotype (frontal bossing with high hairline, ptosis, hypertelorism, broad nasal tip, large anterior fontanelle, cranial base anomalies, and sagittal synostosis (only confirmed in one individual)) were identified on chromosomal microarray to harbour a heterozygous 108.3 kilobase deletion of chromosome 2p21 (disrupting *SIX2* and the surround non-coding DNA) [176].	Red. Further cases required.

SMAD2	• A *de novo* variant in *SMAD2* was identified in an individual within the craniosynostosis cohort of the 100kGP: c.1223T>C; p.(Leu408Pro). The variant was absent from gnomAD and was predicted to affect a residue within a functional domain [82].	Red. Further cases required.
SMARCD2	• An individual was described with metopic synostosis and a *de novo* variant in *SMARCD2*: p.(Arg73*) [152].	Red. Further cases required.
SMC1A	• An X-linked dominant variant (c.3581A>G; p.(Tyr1194Cys)) was identified in an individual with Cornelia de Lange syndrome (characterised by dysmorphic facial features, growth, and developmental delay and syndromic craniosynostosis). Their mother was mosaic for the variant [177].	Red. Further cases required.
SMURF1	• An individual was described with metopic synostosis and a *de novo* variant in *SMURF1*, encoding p.(Arg468Trp) [152].	Red. Further cases required.
SP7	• A *de novo* missense variant (c.926 C>G; p.(Ser309Trp)) in *SP7* was identified in a patient with craniosynostosis, cranial hyperostosis, and long bone fragility. Mice with the corresponding variant also show a complex skeletal phenotype distinct from that of *Sp7*-null mice [178].	Red. Further cases required.
SPRY1	• Single report of a homozygous loss of function variant (c.80T>A; p.(Leu27*)) in a patient with sagittal craniosynostosis, alongside hearing and kidney anomalies. Functional studies show complete absence of the protein and support variant pathogenicity [87].<br>• An individual was described with a heterozygous variant in *SPRY1*: p.(Gln6fs) [152], but evidence suggests that heterozygous loss-of-function variants are not pathogenic (see above reference).	Amber. First human description but with supporting animal models. Further cases required.
SPRY4	• An individual was described with a heterozygous *de novo* variant in *SPRY4*, encoding p.(Glu160*) [152]. This gene has a low pLI (0) suggesting tolerance to loss-of-function variation.	Red. Further cases required.
SRCAP	• One individual was described with a stop-gain variant in *SRCAP* in the Norwegian cohort: c.7303C>T; p.(Arg2435*) [83].	Red. Further cases required.
SUV420H1	• An individual was described with metopic synostosis and a novel *de novo* frameshift variant in *SUV420H1,* encoding p.(Thr97fs) [152].	Red. Further cases required.

TMEM251	• Whole-exome sequencing identified two homozygous variants c.133C>T; p.(Arg45Trp) (absent from gnomAD) and c.215dupA; p.(Tyr72Ter) (2 alleles in gnomAD, annotated as pathogenic in ClinVar), in two families. Immunofluorescence and confocal studies show that the p.(Arg45Trp) mutant TMEM251 protein was targeted less efficiently to the Golgi complex compared to wildtype protein [179].	Red. Further cases required.
TWIST2	• A *de novo* variant was identified within the Timberlake exome sequencing cohort of patients with lambdoid synostosis, encoding p.(Arg64His) [77].	Red. Further cases required.
ZBTB20	• A *de novo* missense variant was identified in an individual from the 100kGP with craniosynostosis, congenital heart disease, intellectual disability, and spinal anomalies. The variant (c.1948A>C) was predicted to encode p.(Asn650His) [82].	Red. Further cases required.
ZCCHC11	• An individual was described with metopic synostosis and a novel *de novo* frameshift variant in *ZCCHC11*: p.(Glu1275fs) [152].	Red. Further cases required.

Table S5 Coordinates of the probes used in the target capture analysis of *SOX6* and *SMAD3*

Gene	Chromosome	Start (GRCh38)	End
SOX6	chr11	15972720	15972840
	chr11	15972780	15972900
	chr11	15972840	15972960
	chr11	15972900	15973020
	chr11	15972960	15973080
	chr11	15973020	15973140
	chr11	15973080	15973200
	chr11	15986131	15986251
	chr11	15986191	15986311
	chr11	15986251	15986371
	chr11	15986311	15986431
	chr11	15986371	15986491
	chr11	15988933	15989053
	chr11	15988993	15989113
	chr11	15989053	15989173
	chr11	15989113	15989233
	chr11	16014875	16014995
	chr11	16014935	16015055
	chr11	16014995	16015115
	chr11	16046427	16046547
	chr11	16046487	16046607
	chr11	16046547	16046667
	chr11	16046607	16046727
	chr11	16046667	16046787
	chr11	16049666	16049786
	chr11	16049726	16049846
	chr11	16049786	16049906
	chr11	16049846	16049966
	chr11	16049906	16050026
	chr11	16055676	16055796
	chr11	16055736	16055856
	chr11	16055796	16055916
	chr11	16055856	16055976
	chr11	16095907	16096027
	chr11	16095967	16096087
	chr11	16096027	16096147
	chr11	16096087	16096207
	chr11	16097528	16097648
	chr11	16097588	16097708
	chr11	16097648	16097768
	chr11	16111712	16111832
	chr11	16111772	16111892
	chr11	16111832	16111952
	chr11	16111892	16112012
	chr11	16183799	16183919
	chr11	16183859	16183979
	chr11	16183919	16184039

	chr11	16186718	16186838
	chr11	16186778	16186898
	chr11	16186838	16186958
	chr11	16186898	16187018
	chr11	16234506	16234626
	chr11	16234566	16234686
	chr11	16234626	16234746
	chr11	16318369	16318489
	chr11	16318429	16318549
	chr11	16318489	16318609
	chr11	16318549	16318669
	chr11	16318609	16318729
	chr11	16340949	16341069
	chr11	16341009	16341129
	chr11	16341069	16341189
	chr11	16341129	16341249
	chr11	16341189	16341309
SMAD3	chr15	67066077	67066197
	chr15	67066137	67066257
	chr15	67066197	67066317
	chr15	67066257	67066377
	chr15	67066317	67066437
	chr15	67164811	67164931
	chr15	67164871	67164991
	chr15	67164931	67165051
	chr15	67164991	67165111
	chr15	67165051	67165171
	chr15	67165168	67165288
	chr15	67165228	67165348
	chr15	67165288	67165408
	chr15	67165348	67165468
	chr15	67166696	67166816
	chr15	67166756	67166876
	chr15	67166816	67166936
	chr15	67170489	67170609
	chr15	67170549	67170669
	chr15	67181166	67181286
	chr15	67181226	67181346
	chr15	67181286	67181406
	chr15	67181346	67181466
	chr15	67181406	67181526
	chr15	67184645	67184765
	chr15	67184705	67184825
	chr15	67184765	67184885
	chr15	67184825	67184945
	chr15	67187286	67187406
	chr15	67187346	67187466
	chr15	67187406	67187526
	chr15	67187466	67187586
	chr15	67190324	67190444

	chr15	67190384	67190504
	chr15	67190444	67190564
	chr15	67190504	67190624

References

1. Mead TJ, Apte SS: **ADAMTS proteins in human disorders**. *Matrix Biol* 2018, **71-72**:225-239.
2. Gabriel LA, Wang LW, Bader H, Ho JC, Majors AK, Hollyfield JG, Traboulsi EI, Apte SS: **ADAMTSL4, a secreted glycoprotein widely distributed in the eye, binds fibrillin-1 microfibrils and accelerates microfibril biogenesis**. *Invest Ophthalmol Vis Sci* 2012, **53**(1):461-469.
3. Bader HL, Wang LW, Ho JC, Tran T, Holden P, Fitzgerald J, Atit RP, Reinhardt DP, Apte SS: **A disintegrin-like and metalloprotease domain containing thrombospondin type 1 motif-like 5 (ADAMTSL5) is a novel fibrillin-1-, fibrillin-2-, and heparin-binding member of the ADAMTS superfamily containing a netrin-like module**. *Matrix Biol* 2012, **31**(7-8):398-411.
4. Tsutsui K, Manabe R, Yamada T, Nakano I, Oguri Y, Keene DR, Sengle G, Sakai LY, Sekiguchi K: **ADAMTSL-6 is a novel extracellular matrix protein that binds to fibrillin-1 and promotes fibrillin-1 fibril formation**. *J Biol Chem* 2010, **285**(7):4870-4882.
5. Ahram D, Sato TS, Kohilan A, Tayeh M, Chen S, Leal S, Al-Salem M, El-Shanti H: **A homozygous mutation in ADAMTSL4 causes autosomal-recessive isolated ectopia lentis**. *Am J Hum Genet* 2009, **84**(2):274-278.
6. Ramirez F: **Fibrillin mutations in Marfan syndrome and related phenotypes**. *Curr Opin Genet Dev* 1996, **6**(3):309-315.
7. Twigg SRF, Wilkie AOM: **A Genetic-Pathophysiological Framework for Craniosynostosis**. *The American Journal of Human Genetics* 2015, **97**:359-377.
8. Aravind L, Landsman D: **AT-hook motifs identified in a wide variety of DNA-binding proteins**. *Nucleic Acids Res* 1998, **26**(19):4413-4421.
9. Khayat MM, Li H, Chander V, Hu J, Hansen AW, Li S, Traynelis J, Shen H, Weissenberger G, Stossi F *et al*: **Phenotypic and protein localization heterogeneity associated with AHDC1 pathogenic protein-truncating alleles in Xia-Gibbs syndrome**. *Hum Mutat* 2021, **42**(5):577-591.
10. Xia F, Bainbridge MN, Tan TY, Wangler MF, Scheuerle AE, Zackai EH, Harr MH, Sutton VR, Nalam RL, Zhu W *et al*: **De novo truncating mutations in AHDC1 in individuals with syndromic expressive language delay, hypotonia, and sleep apnea**. *Am J Hum Genet* 2014, **94**(5):784-789.
11. Lindeboom RGH, Vermeulen M, Lehner B, Supek F: **The impact of nonsense-mediated mRNA decay on genetic disease, gene editing and cancer immunotherapy**. *Nat Genet* 2019, **51**(11):1645-1651.
12. Kitagawa T, Kobayashi D, Baron B, Okita H, Miyamoto T, Takai R, Paudel D, Ohta T, Asaoka Y, Tokunaga M *et al*: **AT-hook DNA-binding motif-containing protein one knockdown downregulates EWS-FLI1 transcriptional activity in Ewing's sarcoma cells**. *PLoS One* 2022, **17**(10):e0269077.
13. Sim JC, White SM, Lockhart PJ: **ARID1B-mediated disorders: Mutations and possible mechanisms**. *Intractable Rare Dis Res* 2015, **4**(1):17-23.
14. Mignot C, Moutard ML, Rastetter A, Boutaud L, Heide S, Billette T, Doummar D, Garel C, Afenjar A, Jacquette A *et al*: **ARID1B mutations are the major genetic cause of corpus callosum anomalies in patients with intellectual disability**. *Brain* 2016, **139**(11):e64.
15. Hempel A, Pagnamenta AT, Blyth M, Mansour S, McConnell V, Kou I, Ikegawa S, Tsurusaki Y, Matsumoto N, Lo-Castro A *et al*: **Deletions and de novo mutations of SOX11 are associated with a neurodevelopmental disorder with features of Coffin-Siris syndrome**. *J Med Genet* 2016, **53**(3):152-162.
16. Lennon MJ, Jones SP, Lovelace MD, Guillemin GJ, Brew BJ: **Bcl11b-A Critical Neurodevelopmental Transcription Factor-Roles in Health and Disease**. *Front Cell Neurosci* 2017, **11**:89.
17. Kyrylkova K, Iwaniec UT, Philbrick KA, Leid M: **BCL11B regulates sutural patency in the mouse craniofacial skeleton**. *Dev Biol* 2016, **415**(2):251-260.

18. Goos JAC, Vogel WK, Mlcochova H, Millard CJ, Esfandiari E, Selman WH, Calpena E, Koelling N, Carpenter EL, Swagemakers SMA *et al*: **A de novo substitution in BCL11B leads to loss of interaction with transcriptional complexes and craniosynostosis**. *Hum Mol Genet* 2019, **28**(15):2501-2513.
19. Bostwick BL, McLean S, Posey JE, Streff HE, Gripp KW, Blesson A, Powell-Hamilton N, Tusi J, Stevenson DA, Farrelly E *et al*: **Phenotypic and molecular characterisation of CDK13-related congenital heart defects, dysmorphic facial features and intellectual developmental disorders**. *Genome Medicine* 2017, **9**(1):73.
20. Rouxel F, Relator R, Kerkhof J, McConkey H, Levy M, Dias P, Barat-Houari M, Bednarek N, Boute O, Chatron N *et al*: **CDK13-related disorder: Report of a series of 18 previously unpublished individuals and description of an epigenetic signature**. *Genet Med* 2022, **24**(5):1096-1107.
21. Hamilton MJ, Caswell RC, Canham N, Cole T, Firth HV, Foulds N, Heimdal K, Hobson E, Houge G, Joss S *et al*: **Heterozygous mutations affecting the protein kinase domain of CDK13 cause a syndromic form of developmental delay and intellectual disability**. *J Med Genet* 2018, **55**(1):28-38.
22. Hamilton MJ, Suri M: **CDK13-related disorder**. *Adv Genet* 2019, **103**:163-182.
23. Adès LC, Sullivan K, Biggin A, Haan EA, Brett M, Holman KJ, Dixon J, Robertson S, Holmes AD, Rogers J *et al*: **FBN1, TGFBR1, and the Marfan-craniosynostosis/mental retardation disorders revisited**. *Am J Med Genet A* 2006, **140**(10):1047-1058.
24. Levi B, Wan DC, Wong VW, Nelson E, Hyun J, Longaker MT: **Cranial suture biology: from pathways to patient care**. *J Craniofac Surg* 2012, **23**(1):13-19.
25. Farmer DT, Mlcochova H, Zhou Y, Koelling N, Wang G, Ashley N, Bugacov H, Chen HJ, Parvez R, Tseng KC *et al*: **The developing mouse coronal suture at single-cell resolution**. *Nat Commun* 2021, **12**(1):4797.
26. Sakai LY, Keene DR, Renard M, De Backer J: **FBN1: The disease-causing gene for Marfan syndrome and other genetic disorders**. *Gene* 2016, **591**(1):279-291.
27. Kipreos ET, Pagano M: **The F-box protein family**. *Genome Biology* 2000, **1**(5):reviews3002.3001.
28. Jin J, Cardozo T, Lovering RC, Elledge SJ, Pagano M, Harper JW: **Systematic analysis and nomenclature of mammalian F-box proteins**. *Genes Dev* 2004, **18**(21):2573-2580.
29. Silverman JS, Skaar JR, Pagano M: **SCF ubiquitin ligases in the maintenance of genome stability**. *Trends Biochem Sci* 2012, **37**(2):66-73.
30. Gregor A, Sadleir LG, Asadollahi R, Azzarello-Burri S, Battaglia A, Ousager LB, Boonsawat P, Bruel AL, Buchert R, Calpena E *et al*: **De Novo Variants in the F-Box Protein FBXO11 in 20 Individuals with a Variable Neurodevelopmental Disorder**. *Am J Hum Genet* 2018, **103**(2):305-316.
31. Duan S, Cermak L, Pagan JK, Rossi M, Martinengo C, di Celle PF, Chapuy B, Shipp M, Chiarle R, Pagano M: **FBXO11 targets BCL6 for degradation and is inactivated in diffuse large B-cell lymphomas**. *Nature* 2012, **481**(7379):90-93.
32. Schieber M, Marinaccio C, Bolanos LC, Haffey WD, Greis KD, Starczynowski DT, Crispino JD: **FBXO11 is a candidate tumor suppressor in the leukemic transformation of myelodysplastic syndrome**. *Blood Cancer J* 2020, **10**(10):98.
33. Shao L, Zhang X, Yao Q: **The F-box protein FBXO11 restrains hepatocellular carcinoma stemness via promotion of ubiquitin-mediated degradation of Snail**. *FEBS Open Bio* 2020, **10**(9):1810-1820.
34. Jansen S, van der Werf IM, Innes AM, Afenjar A, Agrawal PB, Anderson IJ, Atwal PS, van Binsbergen E, van den Boogaard MJ, Castiglia L *et al*: **De novo variants in FBXO11 cause a syndromic form of intellectual disability with behavioral problems and dysmorphisms**. *Eur J Hum Genet* 2019, **27**(5):738-746.

35. Xie Y, Su N, Yang J, Tan Q, Huang S, Jin M, Ni Z, Zhang B, Zhang D, Luo F *et al*: **FGF/FGFR signaling in health and disease**. *Signal Transduct Target Ther* 2020, **5**(1):181.
36. Rodriguez-Zabala M, Aza-Carmona M, Rivera-Pedroza CI, Belinchon A, Guerrero-Zapata I, Barraza-Garcia J, Vallespin E, Lu M, Del Pozo A, Glucksman MJ *et al*: **FGF9 mutation causes craniosynostosis along with multiple synostoses**. *Hum Mutat* 2017, **38**(11):1471-1476.
37. Tang L, Wu X, Zhang H, Lu S, Wu M, Shen C, Chen X, Wang Y, Wang W, Shen Y *et al*: **A point mutation in Fgf9 impedes joint interzone formation leading to multiple synostoses syndrome**. *Hum Mol Genet* 2017, **26**(7):1280-1293.
38. Dobson SM, Kiss C, Borschneck D, Heath KE, Gross A, Glucksman MJ, Guerin A: **Novel FGF9 variant contributes to multiple synostoses syndrome 3**. *Am J Med Genet A* 2022, **188**(7):2162-2167.
39. Yoshida K, Taga T, Saito M, Suematsu S, Kumanogoh A, Tanaka T, Fujiwara H, Hirata M, Yamagami T, Nakahata T *et al*: **Targeted disruption of gpl30, a common signal transducer for the interleukin 6 family of cytokines, leads to myocardial and hematological disorders**. *Proc Natl Acad Sci U S A* 1996, **93**(1):407–411.
40. Shin HI, Divieti P, Sims NA, Kobayashi T, Miao D, Karaplis AC, Baron R, Bringhurst R, Kronenberg HM: **Gp130-mediated signaling is necessary for normal osteoblastic function in vivo and in vitro**. *Endocrinology* 2004, **145**(3):1376-1385.
41. Nieminen P, Morgan NV, Fenwick AL, Parmanen S, Veistinen L, Mikkola ML, van der Spek PJ, Giraud A, Judd L, Arte S *et al*: **Inactivation of IL11 signaling causes craniosynostosis, delayed tooth eruption, and supernumerary teeth**. *Am J Hum Genet* 2011, **89**(1):67-81.
42. Schwerd T, Twigg SRF, Aschenbrenner D, Manrique S, Miller KA, Taylor IB, Capitani M, McGowan SJ, Sweeney E, Weber A *et al*: **A biallelic mutation in IL6ST encoding the GP130 co-receptor causes immunodeficiency and craniosynostosis**. *J Exp Med* 2017, **214**(9):2547-2562.
43. Wiesel-Motiuk N, Assaraf YG: **The key roles of the lysine acetyltransferases KAT6A and KAT6B in physiology and pathology**. *Drug Resist Updat* 2020, **53**:100729.
44. Thomas T, Voss AK, Chowdhury K, Gruss P: **Querkopf, a MYST family histone acetyltransferase, is required for normal cerebral cortex development**. *Development* 2000, **127**:2537-2548.
45. Wu Y, Zhang H, Tang M, Guo C, Deng A, Li J, Wang Y, Xiao L, Yang G: **High methylation of lysine acetyltransferase 6B is associated with the Cobb angle in patients with congenital scoliosis**. *J Transl Med* 2020, **18**(1):210.
46. Huang F, Abmayr SM, Workman JL: **Regulation of KAT6 Acetyltransferases and Their Roles in Cell Cycle Progression, Stem Cell Maintenance, and Human Disease**. *Mol Cell Biol* 2016, **36**(14):1900-1907.
47. Cosentino MS, Oses C, Vázquez Echegaray C, Solari C, Waisman A, Álvarez Y, Petrone MV, Francia M, Schultz M, Sevlever G *et al*: **Kat6b Modulates Oct4 and Nanog Binding to Chromatin in Embryonic Stem Cells and Is Required for Efficient Neural Differentiation**. *Journal of Molecular Biology* 2019, **431**(6):1148-1159.
48. Borgwardt L, Stensland HM, Olsen KJ, Wibrand F, Klenow HB, Beck M, Amraoui Y, Arash L, Fogh J, Nilssen Ø *et al*: **Alpha-mannosidosis: correlation between phenotype, genotype and mutant MAN2B1 subcellular localisation**. *Orphanet J Rare Dis* 2015, **10**:70.
49. Lipiński P, Różdżyńska-Świątkowska A, Iwanicka-Pronicka K, Perkowska B, Pokora P, Tylki-Szymańska A: **Long-term outcome of patients with alpha-mannosidosis - A single center study**. *Mol Genet Metab Rep* 2022, **30**:100826.
50. Atik T, Koparir A, Bademci G, Foster J, 2nd, Altunoglu U, Mutlu GY, Bowdin S, Elcioglu N, Tayfun GA, Atik SS *et al*: **Novel MASP1 mutations are associated with an expanded phenotype in 3MC1 syndrome**. *Orphanet J Rare Dis* 2015, **10**:128.
51. Gajek G, Świerzko AS, Cedzyński M: **Association of Polymorphisms of MASP1/3, COLEC10, and COLEC11 Genes with 3MC Syndrome**. *Int J Mol Sci* 2020, **21**(15).

52. Urquhart J, Roberts R, de Silva D, Shalev S, Chervinsky E, Nampoothiri S, Sznajer Y, Revencu N, Gunasekera R, Suri M *et al*: **Exploring the genetic basis of 3MC syndrome: Findings in 12 further families**. *American Journal of Medical Genetics Part A* 2016, **170**(5):1216-1224.
53. Durmaz CD, Altiner S: **MASP1-related 3MC syndrome in a patient from Turkey**. *Am J Med Genet A* 2021, **185**(7):2267-2270.
54. Rooryck C, Diaz-Font A, Osborn DP, Chabchoub E, Hernandez-Hernandez V, Shamseldin H, Kenny J, Waters A, Jenkins D, Kaissi AA *et al*: **Mutations in lectin complement pathway genes COLEC11 and MASP1 cause 3MC syndrome**. *Nat Genet* 2011, **43**(3):197-203.
55. Senaratne TN, Quintero-Rivera F: **NFIA-Related Disorder**. In: *GeneReviews(®).* Edited by Adam MP, Everman DB, Mirzaa GM, Pagon RA, Wallace SE, Bean LJH, Gripp KW, Amemiya A. Seattle (WA): University of Washington, Seattle; 1993.
56. Uehara T, Sanuki R, Ogura Y, Yokoyama A, Yoshida T, Futagawa H, Yoshihashi H, Yamada M, Suzuki H, Takenouchi T *et al*: **Recurrent NFIA K125E substitution represents a loss-of-function allele: Sensitive in vitro and in vivo assays for nontruncating alleles**. *Am J Med Genet A* 2021, **185**(7):2084-2093.
57. das Neves L, Duchala CS, Tolentino-Silva F, Haxhiu MA, Colmenares C, Macklin WB, Campbell CE, Butz KG, Gronostajski RM: **Disruption of the murine nuclear factor I-A gene (Nfia) results in perinatal lethality, hydrocephalus, and agenesis of the corpus callosum**. *Proc Natl Acad Sci U S A* 1999, **96**(21):11946-11951.
58. Lu W, Quintero-Rivera F, Fan Y, Alkuraya FS, Donovan DJ, Xi Q, Turbe-Doan A, Li QG, Campbell CG, Shanske AL *et al*: **NFIA haploinsufficiency is associated with a CNS malformation syndrome and urinary tract defects**. *PLoS Genet* 2007, **3**(5):e80.
59. Driller K, Pagenstecher A, Uhl M, Omran H, Berlis A, Gründer A, Sippel AE: **Nuclear factor I X deficiency causes brain malformation and severe skeletal defects**. *Mol Cell Biol* 2007, **27**(10):3855-3867.
60. Ogura Y, Uehara T, Ujibe K, Yoshihashi H, Yamada M, Suzuki H, Takenouchi T, Kosaki K, Hirata H: **The p.Thr395Met missense variant of NFIA found in a patient with intellectual disability is a defective variant**. *Am J Med Genet A* 2022, **188**(4):1184-1192.
61. Jones FS, Meech R, Edelman DB, Oakey RJ, Jones PL: **Prx1 Controls Vascular Smooth Muscle Cell Proliferation and Tenascin-C Expression and Is Upregulated With Prx2 in Pulmonary Vascular Disease**. *Circulation Research* 2001, **89**(2):131-138.
62. Donnelly M, Todd E, Wheeler M, Winn VD, Kamnasaran D: **Prenatal diagnosis and identification of heterozygous frameshift mutation in PRRX1 in an infant with agnathia-otocephaly**. *Prenat Diagn* 2012, **32**(9):903-905.
63. Sergi C, Kamnasaran D: **PRRX1 is mutated in a fetus with agnathia-otocephaly**. *Clin Genet* 2011, **79**(3):293-295.
64. Dasouki M, Andrews B, Parimi P, Kamnasaran D: **Recurrent agnathia-otocephaly caused by DNA replication slippage in PRRX1**. *Am J Med Genet A* 2013, **161A**(4):803-808.
65. Celik T, Simsek PO, Sozen T, Ozyuncu O, Utine GE, Talim B, Yigit S, Boduroglu K, Kamnasaran D: **PRRX1 is mutated in an otocephalic newborn infant conceived by consanguineous parents**. *Clin Genet* 2012, **81**(3):294-297.
66. Tooze RS, Miller K, Swagemakers S, Mcgowan S, Boute O, Collet C, Johnson D, Laffargue F, Leeuw ND, Morton J *et al*: **Pathogenic variants in the paired-related homeobox 1 (PRRX1) gene are associated with craniosynostosis**. In: *European Society of Human Genetics Conference; Vienna, Austria*. 2022.
67. Angelozzi M, Lefebvre V: **SOXopathies: Growing Family of Developmental Disorders Due to SOX Mutations**. *Trends in Genetics* 2019, **35**(9):658-671.
68. Tolchin D, Yeager JP, Prasad P, Dorrani N, Russi AS, Martinez-Agosto JA, Haseeb A, Angelozzi M, Santen GWE, Ruivenkamp C *et al*: **De Novo SOX6 Variants Cause a Neurodevelopmental Syndrome Associated with ADHD, Craniosynostosis, and Osteochondromas**. *Am J Hum Genet* 2020, **106**(6):830-845.

69. Yang TL, Guo Y, Liu YJ, Shen H, Liu YZ, Lei SF, Li J, Tian Q, Deng HW: **Genetic variants in the SOX6 gene are associated with bone mineral density in both Caucasian and Chinese populations**. *Osteoporos Int* 2012, **23**(2):781-787.
70. Rivadeneira F, Styrkarsdottir U, Estrada K, Halldorsson BV, Hsu YH, Richards JB, Zillikens MC, Kavvoura FK, Amin N, Aulchenko YS *et al*: **Twenty bone-mineral-density loci identified by large-scale meta-analysis of genome-wide association studies**. *Nat Genet* 2009, **41**(11):1199-1206.
71. Koelling N, Bernkopf M, Calpena E, Maher GJ, Miller KA, Ralph HK, Goriely A, Wilkie AOM: **Amplimap: a versatile tool to process and analyze targeted NGS data**. *Bioinformatics* 2019, **35**(24):5349-5350.
72. Calpena E, Cuellar A, Bala K, Swagemakers SMA, Koelling N, McGowan SJ, Phipps JM, Balasubramanian M, Cunningham ML, Douzgou S *et al*: **SMAD6 variants in craniosynostosis: genotype and phenotype evaluation**. *Genet Med* 2020, **22**(9):1498-1506.
73. Whiffin N, Minikel E, Walsh R, O'Donnell-Luria AH, Karczewski K, Ing AY, Barton PJR, Funke B, Cook SA, MacArthur D *et al*: **Using high-resolution variant frequencies to empower clinical genome interpretation**. *Genet Med* 2017, **19**(10):1151-1158.
74. Lee E, Le T, Zhu Y, Elakis G, Turner A, Lo W, Venselaar H, Verrenkamp CA, Snow N, Mowat D *et al*: **A craniosynostosis massively parallel sequencing panel study in 309 Australian and New Zealand patients: findings and recommendations**. *Genet Med* 2018, **20**(9):1061-1068.
75. Clarke CM, Fok VT, Gustafson JA, Smyth MD, Timms AE, Frazar CD, Smith JD, Birgfeld CB, Lee A, Ellenbogen RG *et al*: **Single suture craniosynostosis: Identification of rare variants in genes associated with syndromic forms**. *Am J Med Genet A* 2018, **176**(2):290-300.
76. Topa A, Rohlin A, Andersson MK, Fehr A, Lovmar L, Stenman G, Kolby L: **NGS targeted screening of 100 Scandinavian patients with coronal synostosis**. *Am J Med Genet A* 2020, **182**(2):348-356.
77. Timberlake AT, Jin SC, Nelson-Williams C, Wu R, Furey CG, Islam B, Haider S, Loring E, Galm A, Yale Center for Genome Analysis *et al*: **Mutations in TFAP2B and previously unimplicated genes of the BMP, Wnt, and Hedgehog pathways in syndromic craniosynostosis**. *Proc Natl Acad Sci U S A* 2019, **116**(30):15116-15121.
78. Suzuki T, Suzuki T, Raveau M, Miyake N, Sudo G, Tsurusaki Y, Watanabe T, Sugaya Y, Tatsukawa T, Mazaki E *et al*: **A recurrent PJA1 variant in trigonocephaly and neurodevelopmental disorders**. *Ann Clin Transl Neurol* 2020, **7**(7):1117-1131.
79. Yoon JG, Hahn HM, Choi S, Kim SJ, Aum S, Yu JW, Park EK, Shim KW, Lee MG, Kim YO: **Molecular Diagnosis of Craniosynostosis Using Targeted Next-Generation Sequencing**. *Neurosurgery* 2020, **87**(2):294-302.
80. Wu Y, Peng M, Chen J, Suo J, Zou S, Xu Y, Wilkie AOM, Zou W, Mu X, Wang S: **A custom-designed panel sequencing study in 201 Chinese patients with craniosynostosis revealed novel variants and distinct mutation spectra**. *J Genet Genomics* 2021, **48**(2):167-171.
81. Alghamdi M, Alhumsi TR, Altweijri I, Alkhamis WH, Barasain O, Cardona-Londono KJ, Ramakrishnan R, Guzman-Vega FJ, Arold ST, Ali G *et al*: **Clinical and Genetic Characterization of Craniosynostosis in Saudi Arabia**. *Front Pediatr* 2021, **9**:582816.
82. Hyder Z, Calpena E, Pei Y, Tooze RS, Brittain H, Twigg SRF, Cilliers D, Morton JEV, McCann E, Weber A *et al*: **Evaluating the performance of a clinical genome sequencing program for diagnosis of rare genetic disease, seen through the lens of craniosynostosis**. *Genet Med* 2021, **23**(12):2360-2368.
83. Tønne E, Due-Tønnessen BJ, Mero IL, Wiig US, Kulseth MA, Vigeland MD, Sheng Y, von der Lippe C, Tveten K, Meling TR *et al*: **Benefits of clinical criteria and high-throughput sequencing for diagnosing children with syndromic craniosynostosis**. *Eur J Hum Genet* 2021, **29**(6):920-929.

84. Chen J, Zhang P, Peng M, Liu B, Wang X, Du S, Lu Y, Mu X, Lu Y, Wang S *et al*: **An additional whole-exome sequencing study in 102 panel-undiagnosed patients: A retrospective study in a Chinese craniosynostosis cohort**. *Front Genet* 2022, **13**:967688.
85. Tønne E, Due-Tønnessen BJ, Vigeland MD, Amundsen SS, Ribarska T, Asten PM, Sheng Y, Helseth E, Gilfillan GD, Mero IL *et al*: **Whole-exome sequencing in syndromic craniosynostosis increases diagnostic yield and identifies candidate genes in osteogenic signaling pathways**. *Am J Med Genet A* 2022, **188**(5):1464-1475.
86. Timberlake AT, Kiziltug E, Jin SC, Nelson-Williams C, Loring E, Yale Center for Genome Analysis, Allocco A, Marlier A, Banka S, Stuart H *et al*: **De novo mutations in the BMP signaling pathway in lambdoid craniosynostosis**. *Hum Genet* 2023, **142**(1):21-32.
87. Tooze RS, Calpena E, Twigg SRF, D'Arco F, Genomics England Research C, Wakeling EL, Wilkie AOM: **Craniosynostosis, inner ear, and renal anomalies in a child with complete loss of SPRY1 (sprouty homolog 1) function**. *J Med Genet.* Epub ahead of print: 21 December 2022, doi: 10.1136/jmg-2022-108946.
88. Parenti I, Lehalle D, Nava C, Torti E, Leitão E, Person R, Mizuguchi T, Matsumoto N, Kato M, Nakamura K *et al*: **Missense and truncating variants in CHD5 in a dominant neurodevelopmental disorder with intellectual disability, behavioral disturbances, and epilepsy**. *Hum Genet* 2021, **140**(7):1109-1120.
89. Colombo EA, Mutlu-Albayrak H, Shafeghati Y, Balasar M, Piard J, Gentilini D, Di Blasio AM, Gervasini C, Van Maldergem L, Larizza L: **Phenotypic Overlap of Roberts and Baller-Gerold Syndromes in Two Patients With Craniosynostosis, Limb Reductions, and ESCO2 Mutations**. *Front Pediatr* 2019, **7**:210.
90. Babbs C, Stewart HS, Williams LJ, Connell L, Goriely A, Twigg SR, Smith K, Lester T, Wilkie AO: **Duplication of the EFNB1 gene in familial hypertelorism: imbalance in ephrin-B1 expression and abnormal phenotypes in humans and mice**. *Hum Mutat* 2011, **32**(8):930-938.
91. Bertola D, Yamamoto G, Buscarilli M, Jorge A, Passos-Bueno MR, Kim C: **The recurrent PPP1CB mutation p.Pro49Arg in an additional Noonan-like syndrome individual: Broadening the clinical phenotype**. *Am J Med Genet A* 2017, **173**(3):824-828.
92. Xing C, Kanchwala M, Rios JJ, Hyatt T, Wang RC, Tran A, Dougherty I, Tovar-Garza A, Purnadi C, Kumar MG *et al*: **Biallelic variants in RNU12 cause CDAGS syndrome**. *Hum Mutat* 2021, **42**(8):1042-1052.
93. Takeyari S, Kubota T, Miyata K, Yamamoto K, Nakayama H, Yamamoto K, Ohata Y, Kitaoka T, Yanagi K, Kaname T *et al*: **Japanese patient with Cole-carpenter syndrome with compound heterozygous variants of SEC24D**. *Am J Med Genet A* 2018, **176**(12):2882-2886.
94. Garbes L, Kim K, Rieß A, Hoyer-Kuhn H, Beleggia F, Bevot A, Kim MJ, Huh YH, Kweon HS, Savarirayan R *et al*: **Mutations in SEC24D, encoding a component of the COPII machinery, cause a syndromic form of osteogenesis imperfecta**. *Am J Hum Genet* 2015, **96**(3):432-439.
95. Zhang H, Yue H, Wang C, Gu J, He J, Fu W, Hu W, Zhang Z: **Novel mutations in the SEC24D gene in Chinese families with autosomal recessive osteogenesis imperfecta**. *Osteoporos Int* 2017, **28**(4):1473-1480.
96. Takenouchi T, Hida M, Sakamoto Y, Torii C, Kosaki R, Takahashi T, Kosaki K: **Severe congenital lipodystrophy and a progeroid appearance: Mutation in the penultimate exon of FBN1 causing a recognizable phenotype**. *Am J Med Genet A* 2013, **161A**(12):3057-3062.
97. Schepers D, Tortora G, Morisaki H, MacCarrick G, Lindsay M, Liang D, Mehta SG, Hague J, Verhagen J, van de Laar I *et al*: **A mutation update on the LDS-associated genes TGFB2/3 and SMAD2/3**. *Hum Mutat* 2018, **39**(5):621-634.
98. Camerota L, Ritelli M, Wischmeijer A, Majore S, Cinquina V, Fortugno P, Monetta R, Gigante L, Hospital MSSGTVU, Sangiuolo FC *et al*: **Genotypic Categorization of Loeys-Dietz Syndrome Based on 24 Novel Families and Literature Data**. *Genes* 2019, **10**(10):764.

99. Baskin SM, Morris SA, Vara A, Hecht JT, Farach LS: **The first reported case of Loeys-Dietz syndrome in a patient with biallelic SMAD3 variants**. *Am J Med Genet A* 2020, **182**(11):2755-2760.
100. Tagariello A, Heller R, Greven A, Kalscheuer VM, Molter T, Rauch A, Kress W, Winterpacht A: **Balanced translocation in a patient with craniosynostosis disrupts the SOX6 gene and an evolutionarily conserved non-transcribed region**. *J Med Genet* 2006, **43**(6):534-540.
101. Chandra A, Aragon-Martin JA, Sharif S, Parulekar M, Child A, Arno G: **Craniosynostosis with ectopia lentis and a homozygous 20-base deletion in ADAMTSL4**. *Ophthalmic Genet* 2013, **34**(1-2):78-82.
102. Gustafson J, Bjork M, van Ravenswaaij-Arts CMA, Cunningham ML: **Mechanism of Disease: Recessive ADAMTSL4 Mutations and Craniosynostosis with Ectopia Lentis**. *Case Rep Genet* 2022, **2022**:3239260.
103. Gumus E: **Extending the phenotype of Xia-Gibbs syndrome in a two-year-old patient with craniosynostosis with a novel de novo AHDC1 missense mutation**. *Eur J Med Genet* 2020, **63**(1):103637.
104. Miller KA, Twigg SR, McGowan SJ, Phipps JM, Fenwick AL, Johnson D, Wall SA, Noons P, Rees KE, Tidey EA *et al*: **Diagnostic value of exome and whole genome sequencing in craniosynostosis**. *J Med Genet* 2017, **54**(4):260-268.
105. Ritter AL, McDougall C, Skraban C, Medne L, Bedoukian EC, Asher SB, Balciuniene J, Campbell CD, Baker SW, Denenberg EH *et al*: **Variable Clinical Manifestations of Xia-Gibbs syndrome: Findings of Consecutively Identified Cases at a Single Children's Hospital**. *Am J Med Genet A* 2018, **176**(9):1890-1896.
106. Danda S, Datar C, Kher A, Deshpande T, Thomas MM, Oommen SP: **First reported cases with Xia-Gibbs syndrome from India harboring novel variants in AHDC1**. *Am J Med Genet A* 2022, **188**(8):2501-2504.
107. Xu J, Yan Q, Song C, Liang J, Zhao L, Zhang X, Weng Z, Xu C, Liu Q, Xu S *et al*: **An Axin2 mutation and perinatal risk factors contribute to sagittal craniosynostosis: evidence from a Chinese female monochorionic diamniotic twin family**. *Hereditas* 2021, **158**(1):20.
108. Yilmaz E, Mihci E, Guzel Nur B, Alper OM: **A novel AXIN2 gene mutation in sagittal synostosis**. *Am J Med Genet A* 2018, **176**(9):1976-1980.
109. Lee KKL, Stanier P, Pauws E: **Mouse Models of Syndromic Craniosynostosis**. *Mol Syndromol* 2019, **10**(1-2):58-73.
110. Justice CM, Musolf AM, Cuellar A, Lattanzi W, Simeonov E, Kaneva R, Paschall J, Cunningham M, Wilkie AOM, Wilson AF *et al*: **Targeted Sequencing of Candidate Regions Associated with Sagittal and Metopic Nonsyndromic Craniosynostosis**. *Genes (Basel)* 2022, **13**(5).
111. Sewda A, White SR, Erazo M, Hao K, García-Fructuoso G, Fernández-Rodriguez I, Heuzé Y, Richtsmeier JT, Romitti PA, Reva B *et al*: **Nonsyndromic craniosynostosis: novel coding variants**. *Pediatric Research* 2019, **85**(4):463-468.
112. Justice CM, Yagnik G, Kim Y, Peter I, Jabs EW, Erazo M, Ye X, Ainehsazan E, Shi L, Cunningham ML *et al*: **A genome-wide association study identifies susceptibility loci for nonsyndromic sagittal craniosynostosis near BMP2 and within BBS9**. *Nat Genet* 2012, **44**(12):1360-1364.
113. Lemyre E, Lemieux N, Décarie JC, Lambert M: **Del(14)(q22.1q23.2) in a patient with anophthalmia and pituitary hypoplasia**. *Am J Med Genet* 1998, **77**(2):162-165.
114. Searle C, Jewell R, Kraft J, Stoebe P, Chumas P, Titheradge H, Kraus A, Gencik M, Hobson E: **Craniosynostosis: a previously unreported association with CHST3-related skeletal dysplasia (autosomal recessive Larsen syndrome)**. *Clin Dysmorphol* 2014, **23**(1):12-15.
115. Mohammadi P, Salehi Siavashani E, Mohammadi MF, Bahramy A, Almadani N, Garshasbi M: **Whole-exome sequencing identified first homozygous frameshift variant in the COLEC10 gene in an Iranian patient causing 3MC syndrome type 3**. *Mol Genet Genomic Med* 2021, **9**(11):e1834.

116. Munye MM, Diaz-Font A, Ocaka L, Henriksen ML, Lees M, Brady A, Jenkins D, Morton J, Hansen SW, Bacchelli C *et al*: **COLEC10 is mutated in 3MC patients and regulates early craniofacial development**. *PLOS Genetics* 2017, **13**(3):e1006679.
117. Balasubramanian M, Pollitt RC, Chandler KE, Mughal MZ, Parker MJ, Dalton A, Arundel P, Offiah AC, Bishop NJ: **CRTAP mutation in a patient with Cole-Carpenter syndrome**. *Am J Med Genet A* 2015, **167a**(3):587-591.
118. Merrill AE, Bochukova EG, Brugger SM, Ishii M, Pilz DT, Wall SA, Lyons KM, Wilkie AO, Maxson RE, Jr.: **Cell mixing at a neural crest-mesoderm boundary and deficient ephrin-Eph signaling in the pathogenesis of craniosynostosis**. *Hum Mol Genet* 2006, **15**(8):1319-1328.
119. Tung ML, Chandra B, Kotlarek J, Melo M, Phillippi E, Justice CM, Musolf A, Boyadijev SA, Romitti PA, Darbro B *et al*: **TBX3 and EFNA4 Variant in a Family with Ulnar-Mammary Syndrome and Sagittal Craniosynostosis**. *Genes (Basel)* 2022, **13**(9).
120. Fonteles CS, Finnell RH, Lei Y, Zurita-Jimenez ME, Monteiro AJ, George TM, Harshbarger RJ: **De novo ALX4 variant detected in child with non-syndromic craniosynostosis**. *Braz J Med Biol Res* 2021, **54**(11):e11396.
121. Hiraki Y, Moriuchi M, Okamoto N, Ishikawa N, Sugimoto Y, Eguchi K, Sakai H, Saitsu H, Mizuguchi T, Harada N *et al*: **Craniosynostosis in a patient with a de novo 15q15-q22 deletion**. *Am J Med Genet A* 2008, **146A**(11):1462-1465.
122. Jehee FS, Bertola DR, Yelavarthi KK, Krepischi-Santos AC, Kim C, Vianna-Morgante AM, Vermeesch JR, Passos-Bueno MR: **An 11q11-q13.3 duplication, including FGF3 and FGF4 genes, in a patient with syndromic multiple craniosynostoses**. *Am J Med Genet A* 2007, **143A**(16):1912-1918.
123. Murakami H, Okawa A, Yoshida H, Nishikawa S, Moriya H, Koseki H: **Elbow knee synostosis (Eks): a new mutation on mouse Chromosome 14**. *Mamm Genome* 2002, **13**(7):341-344.
124. Sentchordi-Montané L, Diaz-Gonzalez F, Cátedra-Vallés EV, Heath KE: **Identification of the third FGF9 variant in a girl with multiple synostosis-comparison of the genotype:phenotype of FGF9 variants in humans and mice**. *Clin Genet* 2021, **99**(2):309-312.
125. Wu XL, Gu MM, Huang L, Liu XS, Zhang HX, Ding XY, Xu JQ, Cui B, Wang L, Lu SY *et al*: **Multiple synostoses syndrome is due to a missense mutation in exon 2 of FGF9 gene**. *Am J Hum Genet* 2009, **85**(1):53-63.
126. Dawson AJ, Hovanes K, Liu J, Marles S, Greenberg C, Mhanni A, Chudley A, Frosk P, Sahoo T, Schanze D *et al*: **Heterozygous intragenic deletions of FREM1 are not associated with trigonocephaly**. *Clin Dysmorphol* 2021, **30**(2):83-88.
127. Vissers LE, Cox TC, Maga AM, Short KM, Wiradjaja F, Janssen IM, Jehee F, Bertola D, Liu J, Yagnik G *et al*: **Heterozygous mutations of FREM1 are associated with an increased risk of isolated metopic craniosynostosis in humans and mice**. *PLoS Genet* 2011, **7**(9):e1002278.
128. Nabais Sá MJ, Miller KA, McQuaid M, Koelling N, Wilkie AOM, Wurtele H, de Brouwer APM, Oliveira J: **Biallelic GINS2 variant p.(Arg114Leu) causes Meier-Gorlin syndrome with craniosynostosis**. *Journal of Medical Genetics* 2022, **59**(8):776-780.
129. Villarreal DD, Villarreal H, Paez AM, Peppas D, Lynch J, Roeder E, Powers GC: **A patient with a unique frameshift mutation in GPC3, causing Simpson-Golabi-Behmel syndrome, presenting with craniosynostosis, penoscrotal hypospadias, and a large prostatic utricle**. *Am J Med Genet A* 2013, **161a**(12):3121-3125.
130. Demir N, Peker E, Ece I, Kaba S, Doğan M, Tuncer O: **A male newborn with Simpson-Golabi-Behmel syndrome, presenting with metopic synostosis, anal atresia, and total anomalous pulmonary venous return**. *Genet Couns* 2014, **25**(4):439-443.
131. Walczak-Sztulpa J, Posmyk R, Bukowska-Olech EM, Wawrocka A, Jamsheer A, Oud MM, Schmidts M, Arts HH, Latos-Bielenska A, Wasilewska A: **Compound heterozygous IFT140 variants in two Polish families with Sensenbrenner syndrome and early onset end-stage renal disease**. *Orphanet J Rare Dis* 2020, **15**(1):36.

132. Pena-Padilla C, Marshall CR, Walker S, Scherer SW, Tavares-Macias G, Razo-Jimenez G, Bobadilla-Morales L, Acosta-Fernandez E, Corona-Rivera A, Mendoza-Londono R *et al*: **Compound heterozygous mutations in the IFT140 gene cause Opitz trigonocephaly C syndrome in a patient with typical features of a ciliopathy**. *Clin Genet* 2017, **91**(4):640-646.
133. Bayat A, Kerr B, Douzgou S: **The evolving craniofacial phenotype of a patient with Sensenbrenner syndrome caused by IFT140 compound heterozygous mutations**. *Clin Dysmorphol* 2017, **26**(4):247-251.
134. Jia S, Yang J, He T, Li W, Luo X, Huang Y, Li C: **Clinical features and mutational analysis of a case with Sensenbrenner syndrome**. *Zhonghua Yi Xue Yi Chuan Xue Za Zhi* 2018, **35**(3):426-428.
135. Arts HH, Bongers EMHF, Mans DA, van Beersum SEC, Oud MM, Bolat E, Spruijt L, Cornelissen EAM, Schuurs-Hoeijmakers JHM, de Leeuw N *et al*: **C14ORF179 encoding IFT43 is mutated in Sensenbrenner syndrome**. *Journal of Medical Genetics* 2011, **48**(6):390-395.
136. Cunningham ML, Horst JA, Rieder MJ, Hing AV, Stanaway IB, Park SS, Samudrala R, Speltz ML: **IGF1R variants associated with isolated single suture craniosynostosis**. *Am J Med Genet A* 2011, **155A**(1):91-97.
137. Zollino M, Lattante S, Orteschi D, Frangella S, Doronzio PN, Contaldo I, Mercuri E, Marangi G: **Syndromic Craniosynostosis Can Define New Candidate Genes for Suture Development or Result from the Non-specifc Effects of Pleiotropic Genes: Rasopathies and Chromatinopathies as Examples**. *Front Neurosci* 2017, **11**:587.
138. Zollino M, Marangi G, Ponzi E, Orteschi D, Ricciardi S, Lattante S, Murdolo M, Battaglia D, Contaldo I, Mercuri E *et al*: **Intragenic KANSL1 mutations and chromosome 17q21.31 deletions: broadening the clinical spectrum and genotype–phenotype correlations in a large cohort of patients**. *Journal of Medical Genetics* 2015, **52**(12):804-814.
139. Bashir RA, Dixit A, Goedhart C, Parboosingh JS, Innes AM, Ferreira P, Hasan SU, Au PB: **Lin-Gettig syndrome: Craniosynostosis expands the spectrum of the KAT6B related disorders**. *Am J Med Genet A* 2017, **173**(10):2596-2604.
140. Kwee ML, Balemans W, Cleiren E, Gille JJ, Van Der Blij F, Sepers JM, Van Hul W: **An autosomal dominant high bone mass phenotype in association with craniosynostosis in an extended family is caused by an LRP5 missense mutation**. *J Bone Miner Res* 2005, **20**(7):1254-1260.
141. Choucair N, Mignon-Ravix C, Cacciagli P, Abou Ghoch J, Fawaz A, Megarbane A, Villard L, Chouery E: **Evidence that homozygous PTPRD gene microdeletion causes trigonocephaly, hearing loss, and intellectual disability**. *Mol Cytogenet* 2015, **8**:39.
142. Simsek-Kiper PO, Taskiran EZ, Kosukcu C, Urel-Demir G, Akgun-Dogan O, Yilmaz G, Utine GE, Nishimura G, Boduroglu K, Alikasifoglu M: **Further delineation of spondyloepimetaphyseal dysplasia Faden-Alkuraya type: A RSPRY1-associated spondylo-epi-metaphyseal dysplasia with cono-brachydactyly and craniosynostosis**. *American Journal of Medical Genetics Part A* 2018, **176**(9):2009-2016.
143. Faden M, AlZahrani F, Mendoza-Londono R, Dupuis L, Hartley T, Kannu P, Raiman JA, Howard A, Qin W, Tetreault M *et al*: **Identification of a Recognizable Progressive Skeletal Dysplasia Caused by RSPRY1 Mutations**. *Am J Hum Genet* 2015, **97**(4):608-615.
144. Maddirevula S, Alsahli S, Alhabeeb L, Patel N, Alzahrani F, Shamseldin HE, Anazi S, Ewida N, Alsaif HS, Mohamed JY *et al*: **Expanding the phenome and variome of skeletal dysplasia**. *Genet Med* 2018, **20**(12):1609-1616.
145. Gonorazky HD, Marshall CR, Al-Murshed M, Hazrati LN, Thor MG, Hanna MG, Männikkö R, Ray PN, Yoon G: **Congenital myopathy with "corona" fibres, selective muscle atrophy, and craniosynostosis associated with novel recessive mutations in SCN4A**. *Neuromuscul Disord* 2017, **27**(6):574-580.

146. Bendon CL, Fenwick AL, Hurst JA, Nürnberg G, Nürnberg P, Wall SA, Wilkie AO, Johnson D: **Frank-ter Haar syndrome associated with sagittal craniosynostosis and raised intracranial pressure**. *BMC Medical Genetics* 2012, **13**:104-112.
147. Horiuchi K, Ariga T, Fujioka H, Kawashima K, Yamamoto Y, Igawa H, Sakiyama Y, Sugihara T: **Treacher Collins syndrome with craniosynostosis, choanal atresia, and esophageal regurgitation caused by a novel nonsense mutation in TCOF1**. *American Journal of Medical Genetics Part A* 2004, **128A**(2):173-175.
148. Bukowska-Olech E, Materna-Kiryluk A, Walczak-Sztulpa J, Popiel D, Badura-Stronka M, Koczyk G, Dawidziuk A, Jamsheer A: **Targeted Next-Generation Sequencing in the Diagnosis of Facial Dysostoses**. *Front Genet* 2020, **11**:580477.
149. Yoshikawa T, Kamei K, Nagata H, Saida K, Sato M, Ogura M, Ito S, Miyazaki O, Urushihara M, Kondo S *et al*: **Diversity of renal phenotypes in patients with WDR19 mutations: Two case reports**. *Nephrology (Carlton)* 2017, **22**(7):566-571.
150. Quinaux T, Custodi V, Putoux A, Bacchetta J, Rossi M, Di Rocco F: **Sensenbrenner syndrome: a further challenge in evaluating sagittal synostosis and a need for a multidisciplinary approach**. *Child's Nervous System* 2021, **37**(5):1695-1701.
151. Hiraki Y, Miyatake S, Hayashidani M, Nishimura Y, Matsuura H, Kamada M, Kawagoe T, Yunoki K, Okamoto N, Yofune H *et al*: **Aortic aneurysm and craniosynostosis in a family with Cantu syndrome**. *American Journal of Medical Genetics Part A* 2014, **164**(1):231-236.
152. Timberlake AT, Furey CG, Choi J, Nelson-Williams C, Yale Center for Genome Analysis, Loring E, Galm A, Kahle KT, Steinbacher DM, Larysz D *et al*: **De novo mutations in inhibitors of Wnt, BMP, and Ras/ERK signaling pathways in non-syndromic midline craniosynostosis**. *Proc Natl Acad Sci U S A* 2017, **114**(35):E7341-E7347.
153. Dinwiddie DL, Soden SE, Saunders CJ, Miller NA, Farrow EG, Smith LD, Kingsmore SF: **De novo frameshift mutation in ASXL3 in a patient with global developmental delay, microcephaly, and craniofacial anomalies**. *BMC Med Genomics* 2013, **6**:32.
154. Hori I, Miya F, Ohashi K, Negishi Y, Hattori A, Ando N, Okamoto N, Kato M, Tsunoda T, Yamasaki M *et al*: **Novel splicing mutation in the ASXL3 gene causing Bainbridge–Ropers syndrome**. *American Journal of Medical Genetics Part A* 2016, **170**(7):1863-1867.
155. Zhao X, Wu B, Chen H, Zhang P, Qian Y, Peng X, Dong X, Wang Y, Li G, Dong C *et al*: **Case report: A novel truncating variant of BCL11B associated with rare feature of craniosynostosis and global developmental delay**. *Front Pediatr* 2022, **10**:982361.
156. Eto K, Machida O, Yanagishita T, Shimojima Yamamoto K, Chiba K, Aihara Y, Hasegawa Y, Nagata M, Ishihara Y, Miyashita Y *et al*: **Novel BCL11B truncation variant in a patient with developmental delay, distinctive features, and early craniosynostosis**. *Human Genome Variation* 2022, **9**(1):43.
157. Calpena E, Hervieu A, Kaserer T, M.A S, Swagemakers, A.C J, Goos, Popoola O, Ortiz-Ruiz MJ, Barbaro-Dieber T *et al*: **De Novo Missense Substitutions in the Gene Encoding CDK8, a Regulator of the Mediator Complex, Cause a Syndromic Developmental Disorder**. *The American Journal of Human Genetics* 2019, **104**:709-720.
158. Snijders Blok L, Rousseau J, Twist J, Ehresmann S, Takaku M, Venselaar H, Rodan LH, Nowak CB, Douglas J, Swoboda KJ *et al*: **CHD3 helicase domain mutations cause a neurodevelopmental syndrome with macrocephaly and impaired speech and language**. *Nat Commun* 2018, **9**(1):4619.
159. Moresco G, Costanza J, Santaniello C, Rondinone O, Grilli F, Prada E, Orcesi S, Coro I, Pichiecchio A, Marchisio P *et al*: **A novel de novo DDX3X missense variant in a female with brachycephaly and intellectual disability: a case report**. *Ital J Pediatr* 2021, **47**(1):81.
160. Scala M, Torella A, Severino M, Morana G, Castello R, Accogli A, Verrico A, Vari MS, Cappuccio G, Pinelli M *et al*: **Three de novo DDX3X variants associated with distinctive brain developmental abnormalities and brain tumor in intellectually disabled females**. *European Journal of Human Genetics* 2019, **27**(8):1254-1259.

161. Deciphering Developmental Disorders S: **Prevalence and architecture of de novo mutations in developmental disorders**. *Nature* 2017, **542**(7642):433-438.
162. Vasileiou G, Vergarajauregui S, Endele S, Popp B, Büttner C, Ekici AB, Gerard M, Bramswig NC, Albrecht B, Clayton-Smith J *et al*: **Mutations in the BAF-Complex Subunit DPF2 Are Associated with Coffin-Siris Syndrome**. *Am J Hum Genet* 2018, **102**(3):468-479.
163. Urreizti R, Mayer K, Evrony GD, Said E, Castilla-Vallmanya L, Cody NAL, Plasencia G, Gelb BD, Grinberg D, Brinkmann U *et al*: **DPH1 syndrome: two novel variants and structural and functional analyses of seven missense variants identified in syndromic patients**. *Eur J Hum Genet* 2020, **28**(1):64-75.
164. Urreizti R, Damanti S, Esteve C, Franco-Valls H, Castilla-Vallmanya L, Tonda R, Cormand B, Vilageliu L, Opitz JM, Neri G *et al*: **A De Novo FOXP1 Truncating Mutation in a Patient Originally Diagnosed as C Syndrome**. *Sci Rep* 2018, **8**(1):694.
165. Rohena L, Lawson M, Guzman E, Ganapathi M, Cho MT, Haverfield E, Anyane-Yeboa K: **FTO variant associated with malformation syndrome**. *American Journal of Medical Genetics Part A* 2016, **170**(4):1023-1028.
166. Barrell WB, Adel Al-Lami H, Goos JAC, Swagemakers SMA, van Dooren M, Torban E, van der Spek PJ, Mathijssen IMJ, Liu KJ: **Identification of a novel variant of the ciliopathic gene FUZZY associated with craniosynostosis**. *European Journal of Human Genetics* 2022, **30**(3):282-290.
167. Dimitri P, Habeb AM, Gurbuz F, Millward A, Wallis S, Moussa K, Akcay T, Taha D, Hogue J, Slavotinek A *et al*: **Expanding the Clinical Spectrum Associated With GLIS3 Mutations**. *J Clin Endocrinol Metab* 2015, **100**(10):E1362-1369.
168. Bryant L, Li D, Cox SG, Marchione D, Joiner EF, Wilson K, Janssen K, Lee P, March ME, Nair D *et al*: **Histone H3.3 beyond cancer: Germline mutations in Histone 3 Family 3A and 3B cause a previously unidentified neurodegenerative disorder in 46 patients**. *Science Advances* 2020, **6**(49):eabc9207.
169. Schwerd T, Krause F, Twigg SRF, Aschenbrenner D, Chen Y-H, Borgmeyer U, Müller M, Manrique S, Schumacher N, Wall SA *et al*: **A variant in IL6ST with a selective IL-11 signaling defect in human and mouse**. *Bone Research* 2020, **8**(1):24.
170. Baple EL, Maroofian R, Chioza BA, Izadi M, Cross HE, Al-Turki S, Barwick K, Skrzypiec A, Pawlak R, Wagner K *et al*: **Mutations in KPTN cause macrocephaly, neurodevelopmental delay, and seizures**. *Am J Hum Genet* 2014, **94**(1):87-94.
171. Yamamoto T, Shimojima K, Ondo Y, Shimakawa S, Okamoto N: **MED13L haploinsufficiency syndrome: A de novo frameshift and recurrent intragenic deletions due to parental mosaicism**. *Am J Med Genet A* 2017, **173**(5):1264-1269.
172. Mahmoud Adel AH, Abdullah AA, Eissa F: **Infantile osteopetrosis, craniosynostosis, and Chiari malformation type I with novel OSTEM1 mutation**. *J Pediatr Neurosci* 2013, **8**(1):34-37.
173. Kim J, Won HH, Kim Y, Choi JR, Yu N, Lee KA: **Breakpoint mapping by whole genome sequencing identifies PTH2R gene disruption in a patient with midline craniosynostosis and a de novo balanced chromosomal rearrangement**. *J Med Genet* 2015, **52**(10):706-709.
174. Fennell AP, Baxter AE, Berkovic SF, Ellaway CJ, Forwood C, Hildebrand MS, Kumble S, McKeown C, Mowat D, Poke G *et al*: **The diverse pleiotropic effects of spliceosomal protein PUF60: A case series of Verheij syndrome**. *Am J Med Genet A* 2022, **188**(12):3432-3447.
175. Rodríguez F, Ponce D, Berward FJ, Lopetegui B, Cassorla F, Aracena M: **RAF1 variant in a patient with Noonan syndrome with multiple lentigines and craniosynostosis**. *Am J Med Genet A* 2019, **179**(8):1598-1602.
176. Hufnagel RB, Zimmerman SL, Krueger LA, Bender PL, Ahmed ZM, Saal HM: **A new frontonasal dysplasia syndrome associated with deletion of the SIX2 gene**. *Am J Med Genet A* 2016, **170A**(2):487-491.

177. Xu Y, Sun S, Li N, Yu T, Wang X, Wang J, Bao N: **Identification and analysis of the genetic causes in nine unrelated probands with syndromic craniosynostosis**. *Gene* 2018, **641**:144-150.
178. Lui JC, Raimann A, Hojo H, Dong L, Roschger P, Kikani B, Wintergerst U, Fratzl-Zelman N, Jee YH, Haeusler G *et al*: **A neomorphic variant in SP7 alters sequence specificity and causes a high-turnover bone disorder**. *Nat Commun* 2022, **13**(1):700.
179. Ain NU, Muhammad N, Dianatpour M, Baroncelli M, Iqbal M, Fard MAF, Bukhari I, Ahmed S, Hajipour M, Tabatabaie Z *et al*: **Biallelic TMEM251 variants in patients with severe skeletal dysplasia and extreme short stature**. *Hum Mutat* 2021, **42**(1):89-101.
